# Spontaneous dewetting transitions of droplets during icing & melting cycle

**DOI:** 10.1038/s41467-022-28036-x

**Published:** 2022-01-19

**Authors:** Lizhong Wang, Ze Tian, Guochen Jiang, Xiao Luo, Changhao Chen, Xinyu Hu, Hongjun Zhang, Minlin Zhong

**Affiliations:** Laser Materials Processing Research Center, Key Laboratory for Advanced Materials Processing Technology (Ministry of Education), Joint Research Center for Advanced Materials & Anti-icing of Tsinghua University (SMSE)-AVIC SARI, School of Materials Science and Engineering, Tsinghua University, 100084 Beijing, P. R. China

**Keywords:** Surfaces, interfaces and thin films, Surface patterning, Mechanical engineering, Wetting

## Abstract

Anti-icing superhydrophobic surfaces have been a key research topic due to their potential application value in aviation, telecommunication, energy, etc. However, superhydrophobicity is easily lost during icing & melting cycles, where the water-repellent Cassie-Baxter state turns to the sticky Wenzel state. The reversible transition during icing & melting cycle without external assistance is challenging but vital for reliable anti-icing superhydrophobic performance, such a topic has rarely been reported. Here we demonstrate a spontaneous Wenzel to Cassie-Baxter dewetting transition during icing & melting cycle on well-designed superhydrophobic surfaces. Bubbles in ice droplets rapidly impact the micro-nano valleys under Marangoni force, prompting the continuous recovery of air pockets during melting processes. We establish models to confirm the bubbles movement broadens the dewetting conditions greatly and present three criteria for the dewetting transitions. This research deepens the understanding of wettability theory and extends the design of anti-icing superhydrophobic surfaces.

## Abstract (web summary)

Despite promising for anti-icing applications, structured superhydrophobic surfaces usually lose their hydrophobicity after a few icing/melting cycles. Here, authors investigate specific structured surfaces and air bubbles on frozen ice droplets to propose three criteria to enable dewetting transitions.

## Introduction

For a long time, icing problems, widely existing in nature, have been a challenge that needs to be solved urgently in many fields such as aviation, telecommunication networks, and power transmission, etc^1–4^. Due to the excellent water-repellency with a contact angle >150° and a sliding angle <10°, superhydrophobic surfaces are considered promising candidates for anti-icing and icephobic applications^5,6^. Generally, the air pockets trapped in the micro-nano valleys of superhydrophobic surfaces can minimum the solid–liquid contact area effectively and support droplets to form the Cassie-Baxter (CB) state, where droplets roll freely at a low tilted angle^7–9^. However, in harsh environmental conditions such as low-temperature and high-humidity environments^10,11^, the trapped air pockets underneath droplets are inevitably impaled, resulting in the transition from the water-repellent CB state to the high-adhesion Wenzel state (C2W)^12^. In Wenzel state, the supercooled droplet is filled in micro-nanostructure, greatly promoting heterogeneous ice nucleation and enhancing ice adhesion strength on the superhydrophobic surfaces^13^. Tavakoli et al. reported that water droplets rapidly transited to the Wenzel state during the cooling process and indicated that the inverse transition could not occur even though the temperature rose to room temperature^14^. Once water droplets transit to the Wenzel state and cannot recover back to the CB state, superhydrophobic surfaces will be sticky and lose water-repellency, failing to retain icephobicity^15–17^. Therefore, realizing the transition from the Wenzel state to the CB state (W2C) is vital for the theoretical research and engineering applications of anti-icing superhydrophobic surfaces.

Generally, one of the CB state and the Wenzel state is metastable, and a large energy barrier exists to make the spontaneous transition to the other impossible^18–25^. To achieve the W2C transition, many researchers have adopted various methods, such as magnetic forces^18^, high-temperature driving forces^19–21^, ultrahigh slenderness ratio^22,23^, and mechanical vibrations^24,25^. However, these external assistances are enormously challenging to apply in practice due to the constraints of actual working conditions and unsatisfied continuous energy input. Yan et al.^26^ reported the condensed droplet jumping by optimizing the micro-groove design of a superhydrophobic surface to avoid the pinned Wenzel state facilely. Nevertheless, this droplet jumping phenomenon existed only when the tiny condensed droplets formed during the condensation process^27–29^. Lou et al.^30^ discovered a spontaneous recovery of tiny droplets from the suspended penetration wetting state (SP) to the CB state on the superhydrophobic surfaces with high micropillars. However, the droplets were not squeezed to the Wenzel state, and the transition from the SP state to the CB state was limited to the nL-scale droplet, larger area fraction, and high micropillars, which leads to difficulties in practical applications. Recently, Yang et al.^31^ noticed that the sticky ice droplets on the grassland-like superhydrophobic surfaces detached under gravity during the melting process. This work offers a clue for the possible dewetting transition during the icing & melting cycle. If the droplets after melting are removed from the micro-nanostructure easily, it will effectively avoid the hazards of re-icing^32^, which is especially worthwhile in aviation applications involving multiple icing & melting cycles^33–35^. To achieve the easy removal of melted droplets, droplets are requested to highly recover to the CB state from the inevitable Wenzel state induced in low-temperature and high-humidity environments. However, the recovery of the CB state and the self-removal ability after droplet melting have not yet been realized, the spontaneous dewetting transitions are rarely reported, and the fundamental researches for icing and melting phenomena on superhydrophobic surfaces are still obscure. Therefore, it is of great significance to achieve a spontaneous W2C transition during the icing & melting cycle and investigate the icing and melting processes of droplets on superhydrophobic surfaces.

In this work, we fabricated four superhydrophobic surfaces with different micro-nanostructures and performed icing & melting tests to observe the transition phenomena. We demonstrated, on the precisely micro-nanostructured highly stable superhydrophobic surface via ultrafast laser ablation, a spontaneous and excellent W2C transition during the icing & melting cycle. The droplets on the surface almost completely transited to the original CB state (97.8% of contact diameter and 98.5% of contact angle) and still maintained a low sliding angle of 3.7° after the icing & melting cycle. This phenomenon did not occur on the other highly stable surfaces. On the well-structured superhydrophobic surface, massive bubbles formed and were frozen in the ice droplets during the icing process. During the melting process, we demonstrated these bubbles rapidly impacted the micro-nano valleys underneath the droplets to prompt the continuous recovery of air pockets and thus the CB state. We established a quasi-static thermal current model to explain the transition mechanism and clarified that the Marangoni force induced by temperature gradient drove bubbles impact to promote the W2C transition. By analyzing the system energy changes during wetting and dewetting processes, we provided the surface design zone for dewetting transitions and demonstrated the bubbles impact greatly broadened the zone, which contradicts the impossibility of the spontaneous transition between two states when one of them is metastable. This dewetting phenomenon only happened on the surfaces with superior superhydrophobicity, superior icing delay property, and appropriate micro-nanostructure sizes as three criteria. Furthermore, we performed multiple icing & melting cycles on our designed surfaces. The contact angles and sliding angles of droplets could still reach 155.6° ± 0.7° and 5.9° ± 0.4° after five cycles, demonstrating the remarkable robustness of the dewetting transition. We believe these discoveries and understanding are impactful for the development of anti-icing superhydrophobic surfaces in theory and applications.

## Results

### Fabrication and characterization of four types of surfaces

**Fig. 1:**
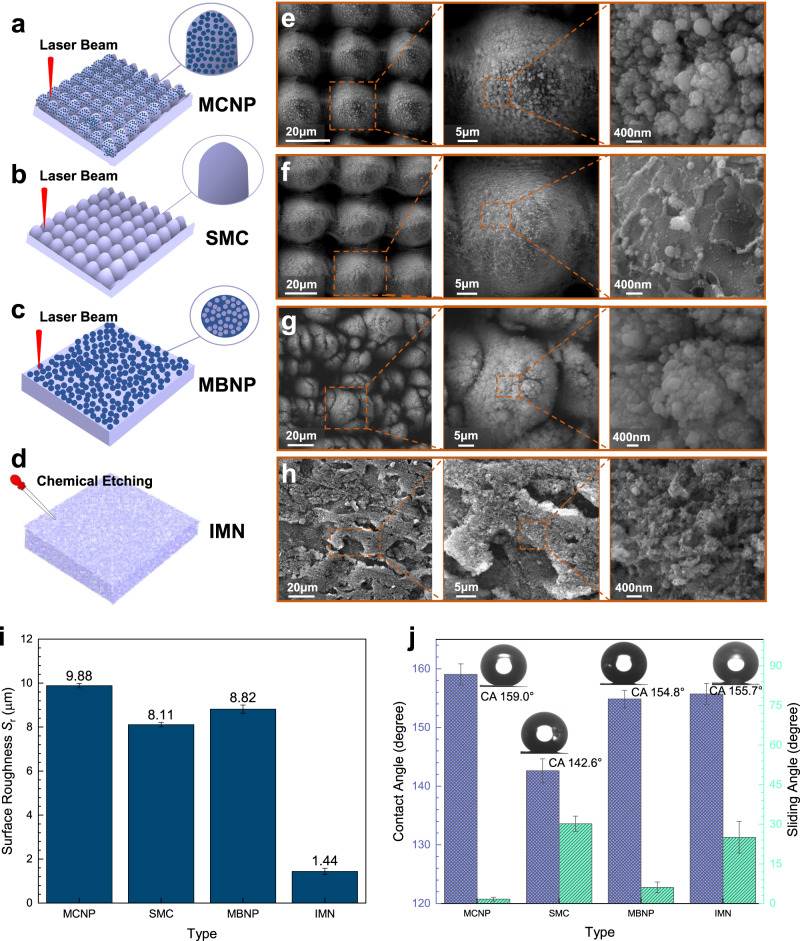
Schematic illustration of fabrication strategies and the topologies of four types of micro-nanostructured surfaces. **a** Double-scale periodical microcones with dense nanoparticles (denoted as MCNP) by laser. **b** Single-scale periodical microcones (SMC) by laser. **c** Double-scale random microbumps with dense nanoparticles (MBNP) by laser. **d** Irregular micro-nanostructure by chemical etching (IMN). SEM images with different magnifications **e** of MCNP, **f** of SMC, **g** of MBNP, and **h** of IMN. Scale bars are marked. **i** Surface roughness of four surfaces. **j** Contact angles (CA) and sliding angles of four surfaces at room temperature (15 °C and a humidity of 20%). Contact angles of four surfaces are marked. Data are mean ± s.d. from at least three independent measurements.

Four micro-nanostructured surfaces were fabricated by ultrafast laser ablation and chemical etching (Fig. 1a–d). The first surface featured double-scale periodical microcones with dense nanoparticles (denoted as MCNP) was produced via one-step slow laser scanning (Fig. 1a). The height *H* and the periodicity Λ of the microcones were fixed at 45 μm and 35 μm, respectively. Nanoparticles with a diameter range of 100–400 nm are attached to the microcones (Fig. 1e). The second surface featured single-scale regular and periodical microcones (denoted as SMC, Fig. 1b) was formed by combining slow laser scanning with fast laser scanning. In the fast-scanning process, most of the nanoparticles on microcones were removed^36^ under the premise of the constant microcones height and periodicity (Fig. 1f and Supplementary Figs. 1 and 2). The third surface composed of double-scale random microbumps with dense nanoparticles (denoted as MBNP, Fig. 1c) was obtained by reducing the laser scanning pitch to be smaller than the laser spot diameter. The diameters of microbumps and nanoparticles range 5–20 μm and 50–100 nm, respectively (Fig. 1g). The fourth surface was produced by immersing aluminum alloy sheets into a boiling aqueous solution of 10 g/L NaOH for 5 minutes, fabricating the irregular micro-nanostructure (denoted as IMN, Fig. 1d). The chemically etched microstructures are ~2–10 μm in depth, and the nanopits are dispersedly distributed on the microstructure surfaces (Fig. 1h). The surface roughness measurements in Fig. 1i indicate that the MCNP surface (*S*_r_ = 9.88 μm) is slightly rougher than the MBNP (*S*_r_ = 8.82 μm) and SMC (*S*_r_ = 8.11 μm), and is much rougher than IMN (*S*_r_ = 1.44 μm). More detailed processing information is given in Supplementary Table 1 and Supplementary Method 1. After being chemically modified by fluoroalkyl silane, three surfaces (MCNP, MBNP, and IMN) become superhydrophobic with contact angles (CA) of $159.0^\circ \pm 1.8^\circ$, $154.8^\circ \pm 1.5^\circ$ and $155.7^\circ \pm 1.8^\circ$, and sliding angles (SA) of $1.6^\circ \pm 0.7^\circ$, $6.1^\circ \pm 2.0^\circ$ and $25.1^\circ \pm 6.0^\circ$ respectively (Fig. 1j). The SMC surface is hydrophobic with contact angles of $142.6^\circ \pm 2.0^\circ$ and sliding angles of $30.2^\circ \pm 2.9^\circ$.

### Icing & melting cycles on the four types of surfaces

**Fig. 2:**
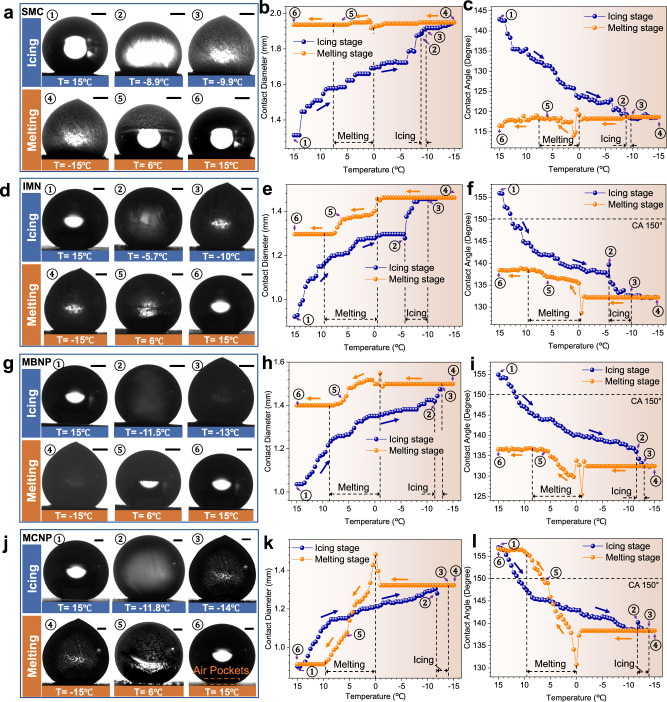
States, contact diameters, and contact angles (CA) of droplets on the four hydrophobic surfaces during icing and melting processes. **a**–**c** On the SMC surface. **d**–**f** On the IMN surface. **g**–**i** On the MBNP surface. **j**–**l** On the MCNP surface. Orange dashed box marks the recovered air pockets in **j**. Blue represents the icing processes while orange denotes the melting processes. Black dashed lines mark icing zones and melting zones. Circulation directions are presented by blue arrows (icing direction) and orange arrows (melting direction). The CA 150° boundaries of superhydrophobicity are indicated with black dashed lines. Scale bars and temperatures corresponding to different states are marked. The scale bars of **a**, **d**, **g** and **j** are 500 μm, 500 μm, 500 μm, and 300 μm, respectively. The fluctuations of the correlation evaluations can be found in the Methods section.

To investigate the droplet states and surface hydrophobicity changes in the icing and melting processes along with the temperature decreases and increases, we performed icing & melting cycle experiments on the four types of surfaces. Detailed experiment methods are described in the Methods section. It can be observed that all the droplets on the four surfaces undergo similar stages during the icing and melting processes. During the icing process, a C2W state transition, recalescence and icing (Fig. 2a) occur in sequence with temperature decreases. The hydrophobicity of the four surfaces gradually deteriorates, resulting in the continuous increase of contact diameters and the continuous decrease of contact angles. During the melting process, there are no apparent changes for all the ice droplets on the four surfaces at first. Until the temperature increases to 0 °C, the contact diameters of droplets suddenly increase sharply, and the contact angles decrease sharply due to the beginning of melting. Subsequently, the hydrophobicity begins to recover by degrees as the melting process proceeds. The contact diameters decrease, and the contact angles increase gradually. However, the recovery extents of the four surfaces’ hydrophobicity are evidently different.

Figure 2a, d, and g depict the common irreversible C2W transition during the icing & melting cycle. In the icing processes, the contact angles of droplets on the MBNP, IMN, and SMC surface decrease from $154.9^\circ$, $155.9^\circ$, and $142.9^\circ$ to $132.4^\circ$, $132.2^\circ$, and $118.6^\circ$ (Fig. 2c, f, i), and the contact diameters increase from 1.04 mm, 0.92 mm, and 1.31 mm to 150 mm, 1.46 mm, and 1.93 mm (Fig. 2b, e, h), respectively. After reaching −15 °C, the melting process starts by turning off the cooling power, and the contact diameters and angles start to recover slowly. Nevertheless, the recovery extents of the contact diameters and angles for the three surfaces are small during the melting processes. The eventually recovered contact angles and diameters can only reach $136.6^\circ$, $138.4^\circ$, $116.5^\circ$, and $1.40\,{{{{{\rm{mm}}}}}}$, $1.30\,{{{{{\rm{mm}}}}}}$, $1.94\,{{{{{\rm{mm}}}}}}$, indicating that the icing-induced impaled droplets on the three surfaces cannot achieve the W2C transitions. Especially for the droplet on the SMC surface, the contact diameters and angles even further increase and decrease during the melting process, respectively. Supplementary Movies 1–3 show the icing & melting cycles on the IMN, MBNP, and SMC surface, respectively.

However, the droplet states on the MCNP surface perform differently in an icing & melting cycle. The original contact angle and the contact diameter of the droplet are $157.0^\circ$ and 0.90 mm, respectively, and the air pockets can be clearly observed (Fig. 2j), therefore it is in the typical CB state. As the temperature decreases, the C2W transition occurs, and the contact diameter gradually increases from 0.90 mm to 1.32 mm while the contact angle decreases from $157.0^\circ$ to $138.3^\circ$ (Fig. 2k, l, blue lines). When the temperature reaches −15 °C, the ice droplet is firmly pinned in the micro-nano valleys of the MCNP surface. Whereafter, the ice droplet starts to melt naturally with the sequential increase of the temperature. Unlike the droplets on the other three surfaces, an apparent spontaneous W2C transition occurs on the MCNP surface during the melting process. The contact diameter rapidly decreases from 1.32 mm to 0.91 mm, and the contact angle increases from 138.34° to 156.69° (Fig. 2k, l, orange lines). The recovered contact diameter and angle are nearly equal to the original ones. Besides, the air pockets are also recovered completely (Fig. 2j). The whole icing & melting cycle on the MCNP surface was recorded in Supplementary Movie 4. Comparison results of the four types of surfaces during the icing & melting cycle are shown in Supplementary Figs. 3–6 and Supplementary Discussion 1.

**Fig. 3:**
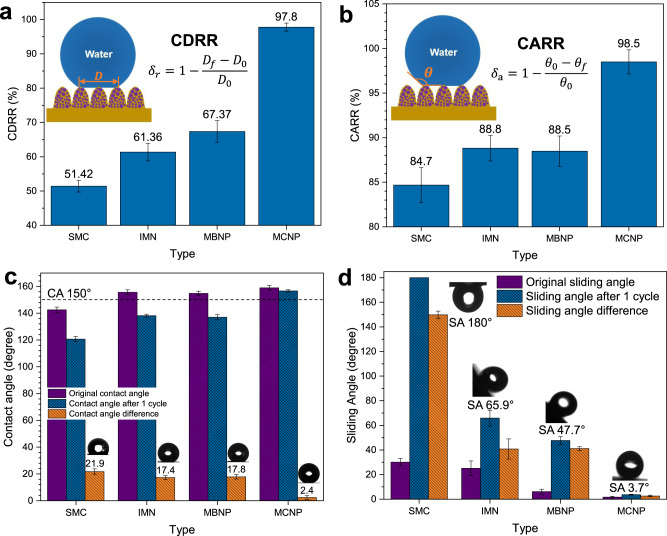
The contact diameter recovery rate (CDRR), the contact angle recovery rate (CARR), contact angle, and sliding angle changes of droplets on the four hydrophobic surfaces after the icing & melting cycle. **a** and **b** CDRR and CARR of droplets on the four surfaces after icing and melting. **c** Comparison of the original contact angles (CA), contact angles after one cycle, and the contact angle differences on the four hydrophobic surfaces. **d** Comparison of the original sliding angles (SA), the sliding angles after one cycle and the sliding angle differences on the four hydrophobic surfaces. The CDRR (*δ*_r_) and CARR (*δ*_a_) represent the CB state recovery extents of droplets. They can be calculated by ${\delta }_{{{{{{\rm{r}}}}}}}=1-\frac{{D}_{{{{{{\rm{f}}}}}}}-{D}_{0}}{{D}_{0}}$ and ${\delta }_{a}=1-\frac{{\theta }_{0}-{\theta }_{{{{{{\rm{f}}}}}}}}{{\theta }_{0}}$, where *D*_f_ and *θ*_f_, *D*_0_ and *θ*_0_ denote the final contact diameter and contact angle after melting, the original contact diameter and angle, respectively. Schematics are inserted in **a** and **b**. The CA 150° boundary of superhydrophobicity is indicated with the black dashed line. Sliding angles of four surfaces after one cycle are marked. Data are mean ± s.d. from at least three independent measurements.

To further characterize the CB state recovery extents of droplets, we compare the contact diameter recovery rate (CDRR) and the contact angle recovery rate (CARR) of droplets on the four surfaces. Apparently, the CDRR of droplets on the SMC, IMN, and MBNP surfaces during icing & melting cycles can only reach 51.42%, 61.36%, and 67.37%, respectively, while that on the MCNP surfaces can reach up to 97.8%, and the CARR also exceeds 98.5% (Fig. 3a, b), both of which further confirm that the droplets on the MCNP surfaces go through a sound W2C transition and nearly recover back to the original states after an icing & melting cycle. By comparing the contact angle differences during icing & melting cycle (Fig. 3c), the contact angles of the droplets on the IMN and MBNP surfaces have greatly decreased after melting. While distinctively for the droplets on the MCNP surfaces, the contact angle after melting can still stay 156.6° ± 0.9°, only decreasing by 2.4° ± 1.4°. Furthermore, the sliding angle of the melted droplets on the MCNP surface hardly increases. Droplets can rapidly roll away at the low tilt angle of only 3.7° (Fig. 3d), further demonstrating the high recovery of the CB state. As the contrasts, the sliding angles on the MBNP and IMN surfaces deteriorate sharply after an icing & melting cycle. Even the droplets on the SMC surfaces cannot yet slide away at a complete inverted angle of 180°, corresponding to the Wenzel state.

## Discussion

### Icing process of droplets on MCNP surfaces

**Fig. 4:**
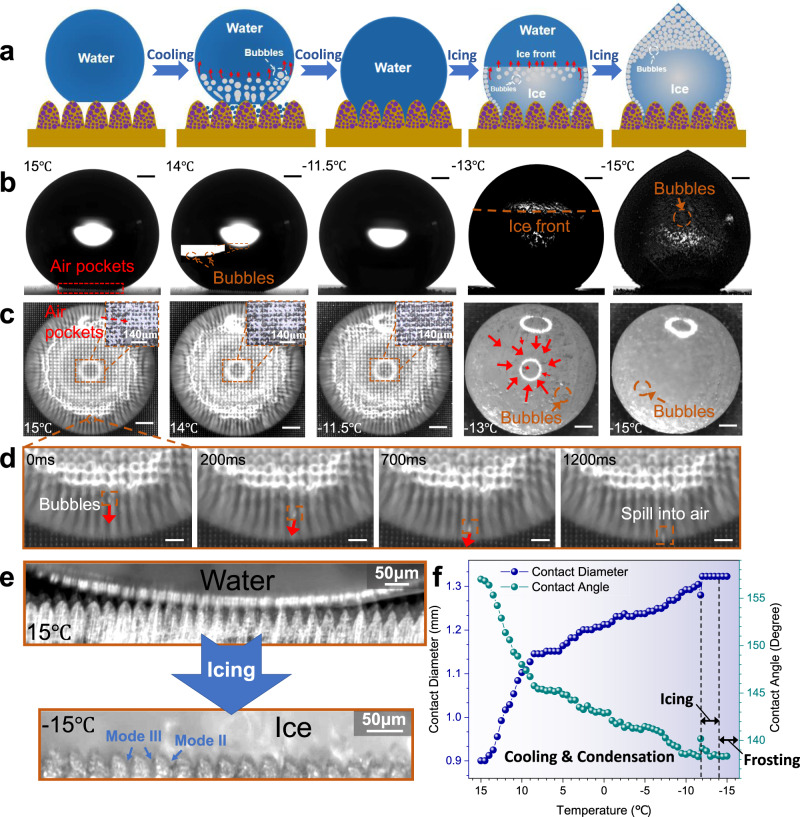
Icing process of a droplet on the MCNP surface. **a** Schematic illustration of icing process. **b** Direct observation from the side view. Proof of bubbles forming in the icing process is shown via the magnified picture. Orange dashed lines indicate the icing front. Bubbles and air pockets are marked with orange dashed boxes and red dashed boxes, respectively. **c** Direct observation from the top view. The magnified observations from the top view for the disappearance of the underneath air pockets are shown in the 1st, 2nd, and 3rd pictures. The zones with lighter contrast in the magnified pictures indicate air pockets, while those with darker contrast indicate the pinned zones. **d** Proof of bubbles movement. The bubble is marked with an orange dashed box. The moving directions of bubbles are marked with red arrows. **e** Observations for the original air pockets at 15 °C and ice penetration in the micro-nanostructure at −15 °C. Ice permeation modes are marked with blue arrows. **f** The changes of contact diameters and angles of the droplet on the MCNP surface with the temperature decrease. Three stages of the icing process are marked by black dashed lines, which indicate the cooling and condensation stage, icing stage and frosting stage, respectively. Scale bars, times and temperatures corresponding to different states are marked. The scale bars of **b**–**d** are 300 μm, 300 μm, and 70 μm, respectively. The fluctuations of the correlation evaluations can be found in the Methods section.

To sufficiently understand the spontaneous and well W2C transition mechanisms, we investigate the icing process of droplets on the MCNP surfaces. Figure 4a depicts the schematic of the icing process on the MCNP surface. According to the experimental results, the whole icing process can be divided into three stages: (i) cooling and condensation stage; (ii) icing stage; and (iii) frosting stage. In the cooling and condensation stage, the droplet on the MCNP surface undergoes a C2W transition and the spreading of the contact diameter (Fig. 4b). These phenomena are mainly due to the intense van der Waals interactions in the micro-nano valleys, leading to vapor condensation and premature condensation^14,37^. Under the condensations, air pockets are pierced gradually, which can be explicitly observed in the magnified pictures for the bottom micro-nano valleys (Fig. 4c, the 1st, 2nd, and 3rd pictures). Meanwhile, the air in air pockets and environmental air constantly dissolve in the droplet or partly form bubbles moving upwards in the droplet (Fig. 4d) since the air solubility in the water droplet increases as temperature decreases.

When the temperature decreases to −11.8 °C, the droplet enters the icing stage, where nucleation and recalescence occur in sequence. At the beginning of recalescence, the droplet bottom solidifies instantaneously while the top remains liquid. Under the volume retraction of the bottom, the contact diameter slightly decreases from 1.28 mm to 1.27 mm, and the contact angle increases from 138.3° to 140.2° (Fig. 4f). Then, with the freezing proceeds, the icing-induced volume expansion leads to the continuous increase in contact diameter and the decrease in contact angle. Noteworthily, since the air solubility in ice is much lower than in liquid water at the same temperature, bubbles are rapidly dissolved out in the ice droplets and escape upwards as the ice front rises (Fig. 4b, c, similar to the solid-gas eutectic^38^). But the escaping speeds of most bubbles ($\frac{2{\rho }_{{{{{{\rm{d}}}}}}}g{r}_{{{{{{\rm{b}}}}}}}^{2}}{9{\eta }_{{{{{{\rm{d}}}}}}}}$, where *r*_b_ is bubble radius, *ρ*_d_ is droplet density, and *η*_d_ is the dynamic viscosity) are much lower than the rising speed of the ice front (~0.14 mm/s), it is therefore difficult for bubbles to escape out from the ice droplet in time, further resulting in the ice droplet containing a large number of bubbles (Fig. 4c, the 4th and 5th pictures). In the frosting stage (−11.8 to −15 °C), the contact diameter and angle are almost constant, maintaining at 1.32 mm and 138.3°, respectively. Some ice whiskers slowly grow on the top of ice droplets (Fig. 4b, the 5th picture).

Figure 4e depicts the ice penetration on the MCNP surface at −15 °C. It can be observed that most ice has been firmly pinned in the micro-nanostructure. In terms of the ice penetration depth, three ice penetration modes are summarized: (i) mode I: ice has almost no permeation into the micro-nanostructure, it is noted as Cassie-ice; (ii) mode II: ice permeates the micro-nanostructure to a certain depth, but not completely; (iii) mode III: ice permeates the micro-nanostructure completely, it is Wenzel-ice. In this work, mode II and mode III exist at the interface between the ice droplet and the MCNP surface. By measuring the ice adhesion strength, it is found that the ice adhesion strength on the MCNP surface gradually increases with the substrate temperature decreases. At −15 *°C*, the ice adhesion strength reaches 364.74 kPa (Supplementary Figs. 7 and 8), even higher than the pristine surface’s (~240 kPa^39^). The above results indicate that the ice droplets have firmly pinned in the micro-nanostructure of the MCNP surfaces, and superhydrophobic surfaces will fail if the ice cannot be removed in time.

### Melting process of ice droplets on MCNP surfaces

**Fig. 5:**
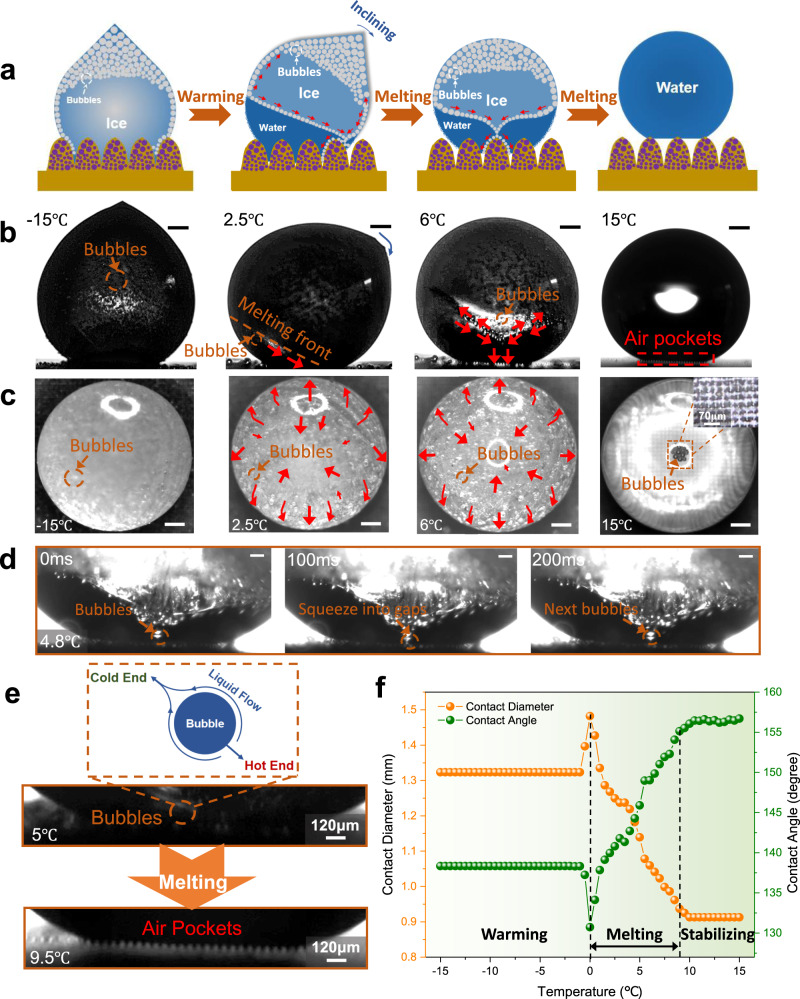
Melting process of an ice droplet on the MCNP surface. **a** Schematic illustration of melting process. **b** Direct observation from the side view. The orange dashed line indicates the melting front. Blue curved arrow represents the inclining direction of the ice droplet. Red arrows indicate the bubble movement directions. Bubbles and air pockets are marked with orange dashed boxes and red dashed boxes, respectively. **c** Direct observation from the top view. The magnified observation for the bottom air pockets is shown in the 4th picture. **d** Proof of bubbles downward movement and squeezing into the bottom micro-nano valleys sequentially. **e** Magnified observations for the recovery of the bottom air pockets with the increase of temperature. A schematic of bubbles movement during the melting stage is depicted. **f** The changes of contact diameters and contact angles of droplets with the temperature increase. Three stages of the melting process are marked by black dashed lines, which are the warming stage, melting stage, and stabilizing stage, respectively. Temperatures, times and scale bars corresponding to different states are marked. The scale bars of **b**–**d** are 300 μm, 300 μm, and 100 μm, respectively. The fluctuations of the correlation evaluations can be found in the Methods section.

When the temperature reaches −15 °C, the Peltier cooling system is powered off so that the surface warms up and ice droplets melt naturally. Figure 5a depicts the melting process schematic on the MCNP surface. Similarly, the melting process consists of three stages: (i) warming stage, (ii) melting stage, and (iii) stabilizing stage. In the first stage of the melting process (−15–0 °C), the ice whiskers melt gradually, and the contact angle and diameter almost remain constant. When the temperature reaches 0 °C, the ice droplet begins to melt from bottom to top directionally. At this time, the ice droplet tends to shake slightly due to the asynchrony of the bottom melting and the uneven density distribution of ice. The bottom of the ice droplet has the trend to spread under the effect of directional melting, leading to a sudden increase in the contact diameter and a decrease in the contact angle (Fig. 5f, the left dashed line).

As the melting stage continues, surface tension differences exist between the melting and non-melting zones due to their temperature differences. The surface tension of the melting zone with higher temperatures is lower, while that of the non-melting zone with lower temperatures is higher. Under the surface tension gradient, small bubbles in the ice droplet migrate continuously from the non-melting zone to the melting zone (Fig. 5b, c). This phenomenon is attributed to the Marangoni effect between the bubbles and the melted droplet. However, for some large bubbles, the Marangoni force *F*_m_ cannot be high enough to overcome their buoyancy *F*_b_ and the water drag *F*_d_ so that these large bubbles will continue moving upwards. Under the multidirectional bubble movements, the ice droplet is often forced to turn over (Fig. 5b, the 2nd picture). In terms of the surface tension equation (${{{{{\mathrm{d}}}}}}\sigma =\frac{\partial \sigma }{\partial T}{{{{{{\mathrm{d}}}}}}T}+\frac{\partial \sigma }{\partial c}{{{{{{\mathrm{d}}}}}}c}$, where *T* is temperature, *c* is concentration), since the temperature difference between the non-melting zone and the melting zone increases gradually as the melting front rises, the Marangoni effect and the non-isothermal fluid flow constantly enhance. Thereby, an inverted conical phase interface forms between the melting and non-melting zones (Fig. 5b, the third picture), where bubbles move along the cone generatrix and rapidly impact downwards after escaping the cone vertex. The high-speed moving bubbles continuously impact the micro-nano valleys to prompt the formation of air pockets (Fig. 5d and Supplementary Movie 5). The bubble impact speed increases exponentially with the increase of temperature difference between the melting zone and the non-melting zone (Supplementary Fig. 9). When the temperature difference reaches 6.5 °C, the impact speed even reaches 4.57 mm/s, which is about twice the droplet diameter (*D*_d_ = 2.122 mm).

Unlike the bubble impact speed, the bubble impact frequency firstly increases and then decreases with the increase of temperature difference (Supplementary Fig. 10). This phenomenon can be attributed to the ice droplet turning over at the early stage of melting so that the melting front height decreases slightly. The lower melting front height results in less water drag to the bubble movement, therefore the bubble impact frequency increases rapidly at first. However, as the melting front rises, the water drag *F*_d_ gradually increases and approaches to *F*_m_ at a quicker rate. Once the critical condition *F*_m_ ≤ *F*_d_ +  *F*_b_ is met, the bubbles will start to move upwards instead of downwards. Therefore, the flux of bubbles subsequently gradually decreases until the melting end. The critical condition for bubbles moving downwards can be expressed as

1 $${\int }_{0}^{2\pi }\frac{{{{{{\mathrm{d}}}}}}\gamma }{{{{{{{\mathrm{d}}}}}}T}}\triangle T\cdot {r}_{{{{{{\rm{bmax}}}}}}}d\alpha -\frac{4\pi }{3}\rho g{r}_{{{{{{\rm{bmax}}}}}}}^{3}-\frac{\pi }{2}C\cdot \rho {v}_{{{{{{\rm{b}}}}}}}^{2}{r}_{{{{{{\rm{bmax}}}}}}}^{2}=0$$

While $\frac{d\gamma }{{dT}}=0.1{{{{{\rm{mN}}}}}}\cdot {{{{{{\rm{m}}}}}}}^{-1}\cdot {{{{{{\rm{K}}}}}}}^{-1}$; Δ*T* is the temperature difference between the substrate and the top unmelted ice; *ρ* is the water density, $\rho \approx 960{{{{{{\rm{kg}}}}}}\cdot {{{{{\rm{cm}}}}}}}^{-3}$; *g* is the gravitational constant, $g\approx 9.8{{{{{\rm{N}}}}}}\cdot {{{{{{\rm{kg}}}}}}}^{-1}$; ${r}_{{{{{{\rm{bmax}}}}}}}$ denotes the maximum bubble radius for moving downwards; *C* is the resistance coefficient of water, $C\approx 5.9\times {10}^{3}$; ${v}_{{{{{{\rm{b}}}}}}}$ is the bubble impact speed. ${r}_{{{{{{\rm{bmax}}}}}}}$ increases gradually with the increase of the temperature difference Δ*T* (Supplementary Fig. 11), indicating that more bubbles move downwards in higher temperature differences. Meanwhile, the bubble size scopes for moving downwards become smaller as the bubble impact speed ${v}_{{{{{{\rm{b}}}}}}}$ increases. Most bubbles in the ice droplets exceed the maximum bubble radius for high-speed moving downwards, hence the bubble impact frequency decreases gradually. After the complete melting, the droplet enters the stabilizing stage, where the contact diameter and angle almost remain constant (Fig. 5f, the right dashed line). Under the aid of rapid bubble impact, the droplet undergoes a spontaneous W2C transition during the melting process and highly recovers to the CB state, with the decrease of contact diameter from 1.32 mm to 0.91 mm and the increase of contact angle from 138.3° to 156.7°. The recovered air pockets can be clearly observed (Fig. 5e, c, the magnified picture).

### Mechanism discussion

**Fig. 6:**
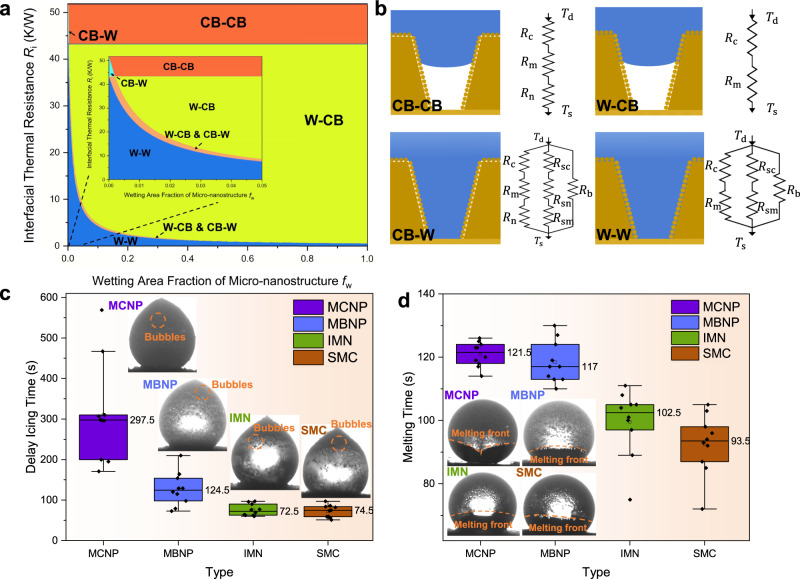
Analysis of the interfacial thermal resistances ***R***_**i**_ in different wetting modes and experimental results for icing delay time and melting delay time on the four surfaces. **a** Phase diagram of interfacial thermal resistances *R*_i_ in different wetting modes. The local magnified diagram is inserted in the center, and different wetting modes are marked. **b** Schematic showing different wetting modes and their equivalent thermal resistances. Droplet temperature (*T*_d_), substrate temperature (*T*_s_) and different equivalent thermal resistances are marked. **c** Median icing delay time on the four surfaces. The icing delay time is recorded from the beginning of the icing process (15 °C) to the recalescence. The side views of ice droplets on the four surfaces are presented. The bubbles of ice droplets are also marked with orange dashed boxes. **d** Median melting delay time on the four surfaces. The melting delay time is recorded from 0 °C to the end of melting. The side views of ice droplets during melting processes are also presented. Melting fronts are marked with orange dashed lines. For each type of surface, 10 repeated experiments are conducted to guarantee the accuracy of the results.

As discussed above, the Marangoni force *F*_m_ is related to the temperature difference Δ*T*, while the Δ*T* is closely related to the interfacial thermal resistance. Therefore, we take the MCNP surfaces as an example and establish a quasi-static thermal current model to analyze the interfacial thermal resistances under different wetting states (Supplementary Fig. 12 and Supplementary Discussion 2). As shown in Fig. 6a, the interfacial thermal resistance between the droplet and the substrate is the smallest when the droplet is in the W-W state, and it rapidly decreases with the increase of the wetting area fraction of the micro-nanostructure *f*_w_. The thermal resistance of the W-W wetting state can be calculated by

2 $${R}_{{{{{{\rm{W}}}}}}-{{{{{\rm{W}}}}}}}=\frac{1}{\pi {r}_{{{{{{\rm{d}}}}}}}^{2}{{{\sin }}}^{2}\theta }\cdot {\left(\frac{{k}_{{{{{{\rm{c}}}}}}}{k}_{{{{{{\rm{m}}}}}}}{f}_{{{{{{\rm{m}}}}}}}}{{\delta }_{{{{{{\rm{c}}}}}}}{k}_{{{{{{\rm{m}}}}}}}+{\delta }_{{{{{{\rm{m}}}}}}}{k}_{{{{{{\rm{c}}}}}}}}+\frac{{k}_{{{{{{\rm{c}}}}}}}{k}_{{{{{{\rm{m}}}}}}}\left(1-{f}_{{{{{{\rm{m}}}}}}}\right)\left(1-{f}_{{{{{{\rm{b}}}}}}}\right){f}_{{{{{{\rm{w}}}}}}}}{{\delta }_{{{{{{\rm{c}}}}}}}{k}_{{{{{{\rm{m}}}}}}}+\frac{{\delta }_{{{{{{\rm{m}}}}}}}{k}_{{{{{{\rm{c}}}}}}}}{2}}+\frac{{k}_{{{{{{\rm{c}}}}}}}\left(1-{f}_{{{{{{\rm{m}}}}}}}\right){f}_{{{{{{\rm{b}}}}}}}{f}_{{{{{{\rm{w}}}}}}}}{{\delta }_{{{{{{\rm{c}}}}}}}}\right)}^{-1}$$

where *k*_*m*_, *k*_*c*_, and *δ*_m_, *δ*_c_ are the thermal conductivity and thickness related to the aluminum alloy microcones and hydrophobic coating, respectively; *f*_m_ and *f*_b_ are the area fraction of the top and the bottom of microcones; *f*_w_ is the wetting area fraction of micro-nanostructure; *r*_d_ is the radius of droplet; and *θ* is the apparent droplet contact angle. When the micro-nanostructure underneath the droplet is thoroughly wetted (Eq. 2, *f*_w_ = 1), the interfacial thermal resistance is the minimum, only 0.20 K W^−1^. This state means the heat from the substrate can be smoothly transferred to the droplet so that the droplet can ice and melt quickly. Meanwhile, the slight temperature difference between the substrate and the melting front leads to the small Marangoni force *F*_m_. In this case, for most bubbles in ice droplets, the critical condition for the bubble downward movement *F*_m_ ≥ *F*_d_ + *F*_b_ is not met. The melting front tends to rise in a straight line.

On the contrary, when the droplet is in the CB-CB state, the bottom air pockets are intact. The thermal resistance is composed of hydrophobic coating, partial nanoparticles, and microcones. It can be given by

3 $${R}_{{{{{{\rm{CB}}}}}}-{{{{{\rm{CB}}}}}}}=\frac{1}{\pi {r}_{{{{{{\rm{d}}}}}}}^{2}{{{\sin }}}^{2}\theta }\cdot \left(\frac{{\delta }_{{{{{{\rm{c}}}}}}}}{{k}_{{{{{{\rm{c}}}}}}}{f}_{{{{{{\rm{m}}}}}}}{f}_{{{{{{\rm{n}}}}}}}}+\frac{{\delta }_{{{{{{\rm{m}}}}}}}}{{k}_{{{{{{\rm{m}}}}}}}{f}_{{{{{{\rm{m}}}}}}}}+\frac{{\delta }_{{{{{{\rm{n}}}}}}}}{{k}_{{{{{{\rm{n}}}}}}}{f}_{{{{{{\rm{m}}}}}}}{f}_{{{{{{\rm{n}}}}}}}}\right)$$

Where *f*_n_, *k*_n_, and *δ*_n_ are the area fraction, the thermal conductivity and thickness of nanoparticles. Under the ideal CB-CB wetting state (Eq. 3), the interfacial thermal resistance reaches 51.71 K W^−1^, larger than any other wetting state. This result indicates that the large thermal resistance in the CB-CB wetting state can delay icing effectively and make melting slower. On the other hand, the large interfacial thermal resistance blocks the heat from the bottom to transfer to the upper non-melting zone directly, forming a significant temperature difference. Under such a large temperature difference, a large surface tension gradient occurs, which drags bubbles downwards to prompt the formation of air pockets, further stimulating the W2C transition. As the air pockets form increasingly, the CB-CB state recovers gradually, contributing to the increase of the interfacial thermal resistances again and making bubbles move downwards more quickly until the bubbles stop moving due to the water drag.

The four surfaces’ icing and melting delay times were tested based on the above discussion for the interfacial thermal resistances. Figure 6c, d shows the differences in the icing and melting delay time of the four surfaces. With the decrease of substrate temperature, MCNP surfaces can significantly delay icing due to high interfacial thermal resistances, and the median of the icing delay time reaches up to 297.5 s, more than 2.3 times longer than the other three surfaces. Meanwhile, longer icing delay time leads to more air dissolution in droplets, further dissolving out more bubbles during icing processes (Supplementary Discussion 3 and Supplementary Figs. 13 and 14). It can be observed that the total bubbles volume in the ice droplet on the MCNP surface is significantly greater than on other surfaces.

During melting processes, more bubbles in the ice droplets on the MCNP surfaces meet the critical condition *F*_m_ ≥ *F*_d_ + *F*_b_ to impact downwards rapidly due to the higher total bubbles volume. The melting front of ice droplets on the MCNP surfaces forms an inverted triangle bubble flow, and lots of bubbles move downwards rapidly, accelerating the W2C transition. Moreover, the melting delay time of ice droplets on the MCNP surface is also longer than that on other surfaces because of the high CB state recovery (Fig. 6d). While for the other three surfaces, the melting processes are more stable, and the melting fronts are almost straight or upward convex. This phenomenon can be attributed to two reasons: one is that the temperature differences between the melting zone and the non-melting zone of droplets are not significant due to poor superhydrophobicity, forming the small downward *F*_m_; the other is that the short icing delay time makes the amounts of bubbles in the ice droplets smaller, hence fewer bubbles meet the critical condition *F*_m_ ≥ *F*_d_ + *F*_b_. Based on the two reasons, almost no bubbles move downwards during the melting processes on the three surfaces, therefore it is more difficult to spontaneously and highly recover to the CB state without the aid of bubbles.

### Theoretical analyses for the dewetting transitions

To acquire the criterion of the dewetting transitions, we analyze the systematic energy changes during wetting and dewetting transitions (Supplementary Figs. 16–19 and Supplementary Discussion 5). Two conditions need to be met for the dewetting transitions. Firstly, the total system energy of the wetted state should be higher than that of the dewetted state. Besides, the energy barriers during the wetting process should be greater than the energy release of the system after wetting, in other words, there are overwhelming energy barriers so that it is almost impossible to overcome them spontaneously. By establishing models to analyze the systematic energy changes during the wetting process (Supplementary Fig. 16), the first condition can be expressed as

4 $${\triangle G}_{1}^{* }\,= \,	{G}_{{{{{{\rm{CB}}}}}}-{{{{{\rm{W}}}}}}}^{* }-{G}_{{{{{{\rm{CB}}}}}}-{{{{{\rm{CB}}}}}}}^{* }=-\left(1-{f}_{1}\right)\\ \,	-\left(1-{f}_{1}\right)\left(1+\frac{2{f}_{1}{H}_{{{{{{\rm{p}}}}}}}}{\left(1-{f}_{1}\right){r}_{{{{{{\rm{p}}}}}}}}\right)\left({f}_{2}{{{{{\rm{cos }}}}}}{\theta }_{0}+{f}_{2}-1\right)-{G}_{{P}_{0}}^{* }\,\ge\, 0$$

While ${\triangle G}_{1}^{* }$ denotes the nondimensionalized Gibbs free energy difference between the CB-W state and the CB-CB state, ${\triangle G}_{1}^{* }=\frac{{\triangle G}_{1}\left(1-{f}_{1}\right)}{{N{{\pi }}{r}_{{{{{{\rm{eff}}}}}}}^{{{{{{\rm{g}}}}}}}}^{2}{\gamma }_{{{{{{\rm{lv}}}}}}}}$; ${r}_{{{{{{\rm{eff}}}}}}}^{{{{{{\rm{g}}}}}}}$ denotes the equivalent geometric radius, $r_{{{{{{\rm{eff}}}}}}}^{{{{{{\rm{g}}}}}}} = \left(\sqrt{\frac{\pi }{{2{f}_{1}}}}-1\right){r}_{{{{{{\rm{p}}}}}}}$; *r*_p_, *N* and *H*_p_ denote the microstructure radius, the total number of microstructures and the microstructure height, respectively. *f*_1_ and *f*_2_ denote the solid fractions of the microstructures and nanostructures, respectively.

For the second condition, the energy barriers during the wetting process mainly consist of the capillary energy barrier and the air pockets energy barrier, while the system energy release is due to the replacement of the solid–air and liquid–air interfaces by the liquid–solid interface. To realize the dewetting spontaneity, the following equation should be met

5 $${\triangle G}_{2}^{* }\,=	 \,\frac{{\triangle G}_{2}}{N\pi {{r}_{{{{{{\rm{eff}}}}}}}^{g}}^{2}{\gamma }_{{{{{{\rm{lv}}}}}}}}=-\left(1-{f}_{1}\right)\frac{2}{1+{{{{{\rm{sin }}}}}}{\theta }_{{{{{{\rm{adv}}}}}}}}\\ 	\,+\left(1-{f}_{1}\right)\left({f}_{2}{{{{{\rm{cos }}}}}}{\theta }_{0}+{f}_{2}-1\right)-(1-{f}_{1})\frac{{r}_{{{{{{\rm{eff}}}}}}}^{{{{{{\rm{g}}}}}}}}{{r}_{{{{{{\rm{eff}}}}}}}^{{{{{{\rm{c}}}}}}}}\frac{2\left({{{{{\rm{sin }}}}}}{\theta }_{{{{{{\rm{adv}}}}}}}-1\right)}{{{{{{\rm{cos }}}}}}{\theta }_{{{{{{\rm{adv}}}}}}}}\\ 	 \;\;\;\;\;({f}_{2}{{{{{\rm{cos }}}}}}{\theta }_{0}+{f}_{2}-1)+{P}_{0}{\varepsilon }_{{{{{{\rm{r}}}}}}}\frac{{r}_{{{{{{\rm{eff}}}}}}}^{{{{{{\rm{g}}}}}}}}{{\gamma }_{{{{{{\rm{lv}}}}}}}}\left[\frac{{{{{{\rm{sin }}}}}}{\theta }_{{{{{{\rm{adv}}}}}}}-1}{{{{{{\rm{cos }}}}}}{\theta }_{{{{{{\rm{adv}}}}}}}}+\frac{{(1-{{{{{\rm{sin }}}}}}{\theta }_{{{{{{\rm{adv}}}}}}})}^{2}(2+{{{{{\rm{sin }}}}}}{\theta }_{{{{{{\rm{adv}}}}}}})}{3{{{{{{\rm{cos }}}}}}}^{3}{\theta }_{{{{{{\rm{adv}}}}}}}}\right]\ge 0$$

While ${r}_{{{{{{\rm{eff}}}}}}}^{{{{{{\rm{c}}}}}}}$ denote the equivalent capillary radius, ${r}_{{{{{{\rm{eff}}}}}}}^{{{{{{\rm{c}}}}}}}=\frac{\left(1-{f}_{1}\right){r}_{{{{{{\rm{p}}}}}}}}{{f}_{1}}$; *p*_0_ denotes the ambient air pressure, *p*_0_ = 101.325 kpa; *θ*_adv_ denotes the advancing contact angle; *ε*_r_ denotes the recovery factor of the bottom air pockets, which is introduced to stand for the bubble contributions for the recovery of the bottom air pockets. The range of *ε*_r_ is from 0 to 1. The first condition guarantees the static energy condition of the dewetting transitions while the second ensures the dynamic energy condition.

**Fig. 7:**
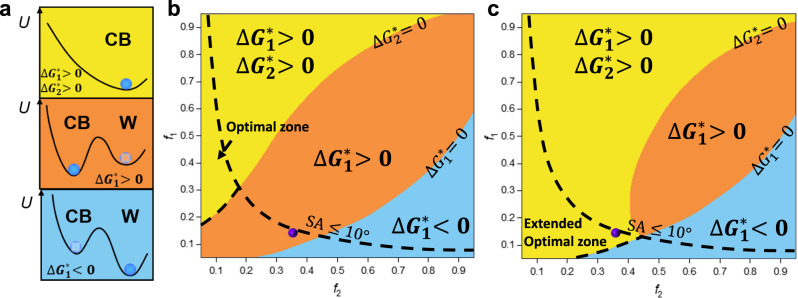
Theoretical analysis for the energy changes during dewetting transitions. **a** Three energy states of droplets on different surfaces. **b** Original phase diagram describing different energy states on surfaces with different micro-nanostructures. Background colors correspond to the distributions of each state, respectively. Yellow denotes the monostable CB zones while orange and blue denote the metastable Wenzel and metastable CB zones, respectively. The black dashed line represents the superhydrophobic conditions, which are described in Supplementary Fig. 15 and Supplementary Discussion 4. The optimal zone for surface design is emphasized by the dashed arrows. The experimental result in this work is marked with the purple point. **c** Phase diagram after bubbles impacting. It is corresponding to the condition that bubbles contribute to the recovery of 3% of the total air pockets volume (*ε*_r_ = 0.03). The extended optimal zone under bubbles impact is emphasized. It can be observed that the droplets on the MCNP surface become the monostable CB state under bubbles impact.

Figure 7a depicts three types of energy states. When ${\triangle G}_{1}^{* } < 0$, the system is in the classical metastable CB wetting state, where the Wenzel state is more stable than the CB state, and a huge energy barrier exists between the two wetting states. While for ${\triangle G}_{1}^{* } > 0$, the CB state becomes more stable, but the system is still in the metastable Wenzel state due to the existence of the energy barrier between the CB state and the Wenzel state. If ${\triangle G}_{2}^{* } > 0$ is met under the premise of ${\triangle G}_{1}^{* } > 0$, the energy barrier between the two wetting states will be enhanced, resulting in the occurrence of the monostable CB state. Even if the droplet in this state is transited to the Wenzel state under external disturbances, the reversible transition will occur when the external disturbances are released. Considering the request of superhydrophobic surfaces for contact angles (CA ≥ 150°) and sliding angles (SA ≤ 10°), the optimal zone for the monostable superhydrophobic surfaces is marked in Fig. 7b. It can be observed that the zone area is small, and the monostability cannot be realized on most surfaces. Based on the former experimental phenomena that the bubbles in ice droplets impact the bottom micro-nano valleys rapidly to prompt the recovery of the air pockets during the melting process, we assume the bubbles impact contributes to 3% of the air pockets to recover (*ε*_r_ = 0.03, which can be realized in terms of the calculation in Supplementary Discussion 3). The optimal zone under bubbles impact can be seen in Fig. 7c. The optimal zone is significantly extended so that the surfaces with the original bistable state can also be transited to the monostable state to realize the dewetting transitions effectively during icing & melting cycle.

### Effects of surface resistance on the dewetting transitions

**Fig. 8:**
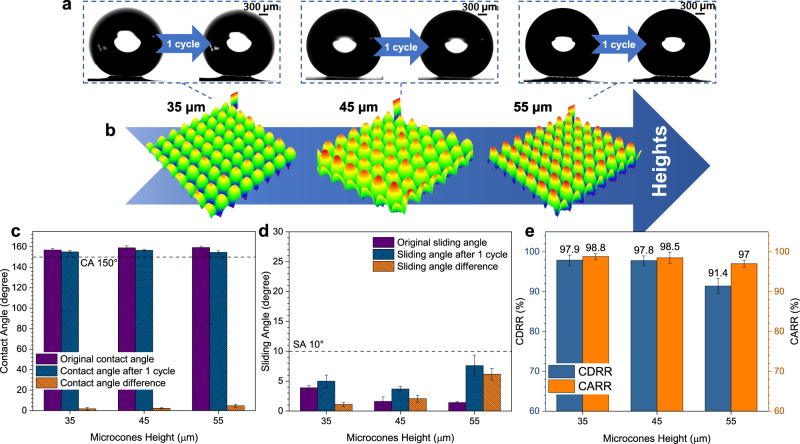
Effects of different microcones heights on the recovery of CB state. **a** State changes of droplets on the MCNP surfaces with different microcones heights after an icing & melting cycle. **b** Morphology evolutions of micro-nanostructure with different microcones heights. By adjusting laser processing parameters (Supplementary Table 2), we achieve precisely tunable heights and pitches of the micro-nanostructure on the MCNP surfaces. Two kinds of surfaces are fabricated: the surfaces with different microcones heights and constant pitch and the surfaces with different microcones pitches and constant height (Supplementary Figs. 20–22 and Supplementary Method 2). In the figure, microcones pitch is fixed at 35 μm while microcones heights vary from 35 μm to 55 μm. **c**–**e** Effects of different microcones heights on the contact diameter recovery rates (CDRR), the contact angle recovery rates (CARR), contact angles (CA), and sliding angles (SA), respectively. The CA 150° and SA 10° boundaries of superhydrophobicity are indicated with the black dashed line. Data are mean ± s.d. from at least three independent measurements.

**Fig. 9:**
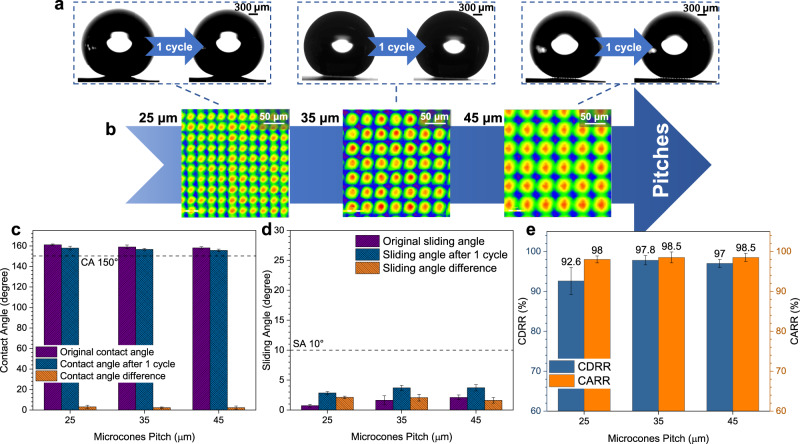
Effects of different microcones pitches on the recovery of CB state. **a** State changes of droplets on the MCNP surfaces with different microcones pitches after an icing & melting cycle. **b** Morphology evolutions of micro-nanostructure with different microcones pitches. Microcones height is fixed at 45 μm while microcones pitches vary from 25 μm to 45 μm. **c**–**e** Effects of different microcones pitches on the contact diameter recovery rates (CDRR), the contact angle recovery rates (CARR), contact angles (CA), and sliding angles (SA), respectively. The CA 150° and SA 10° boundaries of superhydrophobicity are indicated with the black dashed line. Data are mean ± s.d. from at least three independent measurements.

Considering that different micro-nanostructures may cause different resistances to the recovery of the CB state, icing & melting cycle experiments on the MCNP surface with different microcones heights and pitches were conducted (Figs. 8 and 9). With the increase of microcones height, the CDRR and CARR of droplets gradually decrease after an icing & melting cycle (Fig. 8a, b, e). When the microcones height increases to 55 μm, the CDRR and CARR decrease to 91.43% and 97%, the contact angle difference slightly increases, and the sliding angle difference significantly increases to 6.2° (Fig. 8c–e). These phenomena are mainly since the higher micro-nanostructure blocks bubble movement and droplet retraction, resulting in a more difficult recovery of the CB state. Similarly, with the increase of the microcones pitch (Fig. 9a, b), the droplets have less resistance to transit to the CB state. Hence, the CDRR and CARR show a slightly increasing trend while the contact angle and sliding angle differences decrease gradually (Fig. 9c–e). However, as the microcones pitch increases to a specific value, the superhydrophobicity starts to deteriorate. Droplets penetrate the micro-nanostructure more easily, further resulting in more difficulties for droplets to recover back to the CB state, therefore the CDRR and CARR slightly decrease when the microcones pitch increases to 45 μm. The optimal microcones heights and pitches are further explored in Supplementary Figs. 23–25 and Supplementary Discussion 6.

In addition, we conducted the icing & melting cycles on the other six kinds of superhydrophobic surfaces with different micro-nanostructures (Supplementary Figs. 26–29, Supplementary Table 4, and Supplementary Discussion 7), which were reported as the highly stable surfaces^40–42^. It is observed that although the more abundant micro-nanostructure can improve the CB stability and reduce the ice adhesion strength markedly, it cannot avoid the C2W transition during the continuous cooling process. Moreover, the rich micro-nanostructure also places more barriers to the recovery of air pockets during melting, making it difficult to transit to the CB state after an icing & melting cycle (Supplementary Figs. 30 and 31 and Supplementary Discussion 8). Therefore, the surface resistance of the micro-nanostructure plays a vital role in the W2C transition and high recovery of the CB state.

From the above discussion, the recovery of CB state is related to surface superhydrophobicity, bubbles movement, and micro-nanostructure. Therefore, to achieve a spontaneous dewetting transition and high recovery of the CB state, there are three criteria to be met: (i) superior superhydrophobicity, guaranteeing the bottom air pockets cannot be easily pierced, is the key for the W2C transition; (ii) superior icing delay property, resulting in more air dissolved in the droplets and more bubbles dissolved out in the ice droplets; (iii) appropriate micro-nanostructure sizes with less surface resistance, ensuring bubbles can move swimmingly and droplets can retract smoothly, is essential for the high recovery of the CB state. The detailed analysis for the three criteria can be found in Supplementary Discussions 3–5 and 8, respectively.

### Robustness tests for the dewetting transition

**Fig. 10:**
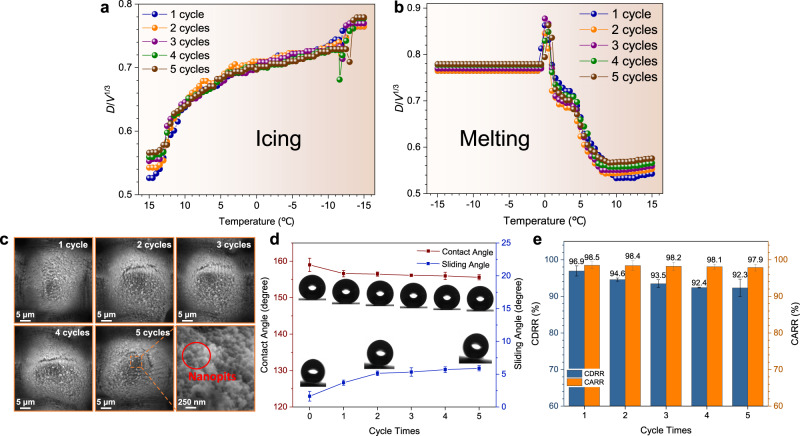
Robustness tests of the W2C transition. **a** The $\frac{D}{{V}^{\frac{1}{3}}}$ changes of the same droplet on the same location with the temperature decrease after 1–5 cycles. It corresponds to the icing process. *D* represents the contact diameters of droplets while *V* represents the droplet volumes. **b** The $\frac{D}{{V}^{\frac{1}{3}}}$ change of the same droplet on the same location with increasing temperature after 1–5 cycles. It corresponds to the melting process. **c** Evolutions of surface micro-nano structures after multiple cycles. The formed nanopits are noted by the red circle. Cycle times and scale bars are marked. **d** Evolutions of contact angles and sliding angles of the same dropl**e**t after multiple cycles. **e** The changes of the contact diameter recovery rates (CDRR) and the contact angle recovery rates (CARR) of droplets on the MCNP surface with the increase of cycle times. Data are mean ± s.d. from at least three independent measurements.

By conducting the repeated icing & melting tests for a droplet (5 μL) on the MCNP surface, the robustness of the dewetting transition on the MCNP surfaces is investigated. Considering the influence of volume evaporation on the contact diameter of droplets after multiple icing & melting cycles, $\frac{D}{{V}^{\frac{1}{3}}}$ is adopted as the dependent variable (where *D* denotes contact diameter, *V* is the droplet volume). As shown in Fig. 10a, b, the $\frac{D}{{V}^{\frac{1}{3}}}$ deteriorates slightly with the cycle times increase, but the overall changes during five cycles are not significant. After five icing & melting cycles, the $\frac{D}{{V}^{\frac{1}{3}}}$ increases from 0.53 to 0.57, only deteriorating by 7.5%. The contact angle and sliding angle can still maintain 155.6° ± 0.72° and 5.9° ± 0.4° (Fig. 10d). The CDRR and CARR of droplets decrease slowly with the cycle times increase and still reach 92.3% and 97.9% after five icing & melting cycles (Fig. 10e). The slight deterioration of recovery ability can be attributed to the damage of the micro-nanostructure (Fig. 10c), where partial nanoparticles are peeled off from the top of microcones during the icing & melting cycle. However, the peeling depth of nanoparticles from microcones is shallow and gradually tends to be constant after multiple icing & melting cycles. Most zones of microcones are still covered with abundant nanoparticles and nanopits formed in the shedding zones of nanoparticles. These micro-nanostructures also guarantee the MCNP surfaces can still meet the criteria (i) after multiple icing & melting cycles, therefore the droplets on the MCNP surface still have a sound W2C transition after multiple icing & melting cycles. Furthermore, we performed icing & melting cycle tests under different conditions including different temperatures, humidities, droplet sizes, ice types and dynamic impacting conditions (Supplementary Figs. 32–42, Supplementary Discussions 9–15, and Supplementary Table 5). It is found that similar dewetting phenomena still exit on the MCNP surfaces during the icing & melting cycle, which further confirms the dewetting robustness of the MCNP surfaces.

In conclusion, we report a spontaneous and sound recovery of the CB state from the Wenzel state on the MCNP surface during the icing & melting cycle. Droplets’ CB state and self-removal ability on the MCNP surface can be completely recovered after the icing & melting cycle. Based on the further investigations on the icing and melting processes, we demonstrate that a significant number of bubbles frozen in the ice droplets rapidly impact the micro-nano valleys under Marangoni force during the melting process, prompting the W2C transition. By establishing models, we explain the inner mechanisms and confirm that the bubble impact greatly broadens the dewetting transition conditions for the micro-nanostructure of superhydrophobic surfaces, which leads to the occurrence of the spontaneous W2C transition even if the surface is in the metastable state. This result not only contradicts the former theory that spontaneous transition cannot occur when in the metastable state but also enlarges the design paths of anti-icing superhydrophobic surfaces greatly. Besides, superhydrophobicity, icing delay time, and the micro-nanostructure all influence the occurrence of the W2C transition and the recovery extents of the CB state. For more applications, we reveal three criteria for the spontaneous and high recovery of the CB state to guide the better design of anti-icing superhydrophobic surfaces. The remarkable W2C transition robustness of the MCNP surface is confirmed by multiple icing & melting cycle tests and other tests under different environmental conditions. Our findings might contribute to understanding the general icing and melting phenomena in nature. We expect our research on the bubble dynamics during the icing & melting cycle could pave a path to the development of wettability theory and superhydrophobic applications in many fields.

## Methods

### Micro-nanostructure fabrication

A Trumpf TruMicro 5000 ultrafast laser system with 800 fs pulses at a central wavelength of 1030 nm and a repetition rate of 200 kHz, was utilized for the micro-nanostructure fabrication of SMC, MBNP, and MCNP surfaces. Before laser processing, aluminum alloy samples (from BondHus, 6061) were mechanically polished to a mirror finish and cleaned ultrasonically with ethanol for 5 min. An x−y galvo was used to focus and scan the laser beam on the surfaces in a pattern of crossed lines in an atmospheric environment. The IMN surfaces were fabricated by chemical etching. The cleaned aluminum alloy samples were immersed in a boiling aqueous solution of 10 g/L NaOH for 5 min to etch out micro-nanostructures. Then, all four types of samples were washed with ethanol and deionized water to remove the unstable micro-nanostructure and were slowly dried with pure nitrogen. At last, all the micro-nanostructured surfaces were immersed in the 1H,1H,2H,2H-perfluorodecyltrimethoxysilane alcohol solution with a concentration of 1% for 2 h and dried in an oven at 90 °C for 60 min. After these processes, the surfaces became superhydrophobic. Laser fabrication equipment, laser parameters, and performances are shown in Supplementary Figs. 44–48, Supplementary Table 3, and Supplementary Method 3.

### Characterization and measurement

The morphology of all the samples was examined by the field-emission scanning electron microscopy (SEM, TESCAN MIRA 3 LMH) equipped with an energy-dispersive spectroscope (EDS, Oxford). The 3D topography measurement was characterized using a 3D digital optical microscope (OLYMPUS LEXT 4100).

Contact angle and sliding angle measurements were carried out to evaluate the wettability of various surfaces by a video-based optical contact angle measuring device (OCA 15 Plus from Data Physics Instruments). The droplets in this work are deionized and purified water with a volume of 5 μL. The contact and sliding angles of every sample were examined at different randomly selected locations at least three times.

### Icing & melting cycle experiments

All samples were placed horizontally on a Peltier colling plate (TEC2-19006), with both sides fixing a dual-probe temperature recorder with a measuring accuracy of 0.1 °C to record the real-time temperature. The surrounding temperature and humidity were sustained at 15 °C and 20%. The water-chilling plant was installed under the Peltier cooling plate. The thermal conductive silicone (RG-ICFN-200G-B1) was smeared evenly in the bonding surfaces of the Peltier cooling plate and the water-chilling plant. By running the cooling system, the temperature of the samples gradually decreased from 15 °C to −15 °C at a specific rate. When the temperature reached −15 °C, the cooling system was turned off, and then the sample temperature naturally rose back to 15 °C. A CCD camera with an adjustable magnification lens was positioned horizontally to record the side view of the droplet in real-time, with 10 frames per second and 0.2 μm of resolution. The schematic diagram of the experiment setup and the temperature curves are shown in Supplementary Figs. 43 and 33, respectively.

The contact and sliding angles of droplets during icing and melting processes were recorded by a real-time video of the OCA measurement device. Then, by using image processing software, the contact diameters of droplets in different temperatures were measured. The icing & melting cycle experiments were conducted at randomly selected locations on each sample’s surface. The standard deviation of the measured contact angles is less than 3° for CA ≥150° and around 5° for CA≤140°. The uncertainty in the contact diameter evaluation is 0.002–0.03 mm (average standard deviation is 0.02 mm). The CDRR, CARR, contact angles and sliding angles are averaged over three measurements.

### Ice adhesion strength measurement

The setup for measuring the ice adhesion strength is shown in Supplementary Fig. 7. It was performed by the Peltier cooling plate and a force transducer (Imada ZP-100N), which is connected to a computer. Because of the excellent icing delay effects of MCNP surfaces, we chose −11 °C, −13 °C, and −15 °C as the experimental temperatures of ice adhesion strength. After turning on the Peltier cooling plate, the temperature of sample surfaces started to decrease. When the droplets iced and reached the objective temperature, the force transducer probe was driven to push the ice droplets at a speed of 0.5 mm/s. The peak forces applied to detach ice droplets were recorded in the software. By calculating the ratio of the peak force and the ice droplet cross-sectional area, the ice adhesion strengths were acquired. The force accuracy is up to 0.01 N. The ice adhesion strength at each temperature is measured at least three times.

### Reporting summary

Further information on research design is available in the Nature Research Reporting Summary linked to this article.

## Supplementary information

Supplementary Information

Description of Additional Supplementary Files

Supplementary Movie 1

Supplementary Movie 2

Supplementary Movie 3

Supplementary Movie 4

Supplementary Movie 5

Lasing Reporting Summary

## Data Availability

The data that support the findings of this study are available from the corresponding authors upon reasonable request.
