# Supplementary material for: Spontaneous dewetting transitions of droplets during icing & melting cycle
Source: Nat Commun. 2022 Jan 19;13:378. doi: 10.1038/s41467-022-28036-x (PMC8770474; doi:10.1038/s41467-022-28036-x)
Supplement: Supplementary file 1 — Supplementary Information [file 41467_2022_28036_MOESM1_ESM.pdf]

Supplementary Information

Spontaneous Dewetting Transitions of Droplets during Icing & Melting Cycle

Lizhong Wang[1], Ze Tian[1], Guochen Jiang[1], Xiao Luo[1], Changhao Chen[1], Xinyu Hu[1], Hongjun Zhang[1] & Minlin Zhong[1]*

[1] Laser Materials Processing Research Center, Key Laboratory for Advanced Materials Processing Technology (Ministry of Education), Joint Research Center for Advanced Materials & Anti-icing of Tsinghua University (SMSE)-AVIC SARI, School of Materials Science and Engineering, Tsinghua University, Beijing 100084, P. R. China. *email: zhml@tsinghua.edu.cn

This PDF file includes:

Supplementary Figures 1- 48

Supplementary Tables 1- 5

Supplementary Method 1. Laser processing parameters and topologies of the samples

Supplementary Method 2. Laser processing parameters, topologies and wettability of the MNCP surfaces with different microcones heights and pitches.

Supplementary Method 3. Experimental setup

Supplementary Discussion 1. Comparison and records of droplets on four types of surfaces during icing & melting cycle

Supplementary Discussion 2. The interfacial thermal resistance of different wetting states

Supplementary Discussion 3. Calculation of total bubbles volume in the ice droplet

Supplementary Discussion 4. Design principles of superhydrophobic surfaces

Supplementary Discussion 5. Theoretical analysis for the wetting and dewetting transitions

Supplementary Discussion 6. Optimal design zone for the superhydrophobic surfaces fabricated by ultrafast laser

Supplementary Discussion 7. Icing & melting tests on the surfaces with different Cassie-Baxter stability

Supplementary Discussion 8. Surface resistances for droplet retraction during the melting process

Supplementary Discussion 9. Solar-assisted melting experiments

Supplementary Discussion 10. Effects of different substrate temperature on the dewetting transitions

Supplementary Discussion 11. Effects of different room temperatures on the dewetting transitions

Supplementary Discussion 12. Effects of different environmental humidities on the dewetting transitions

Supplementary Discussion 13. Effect of droplet volumes on the dewetting transitions

Supplementary Discussion 14. Effects of different ice types on the dewetting transitions

Supplementary Discussion 15. Droplet dynamic impacting on the MCNP surfaces

Other Supplementary Materials for this paper include:

Supplementary Movie 1: Icing & melting cycle of a droplet on the IMN surface (AVI)

Supplementary Movie 2: Icing & melting cycle of a droplet on the MBNP surface (AVI)

Supplementary Movie 3: Icing & melting cycle of a droplet on the SMC surface (AVI)

Supplementary Movie 4: Icing & melting cycle of a droplet on the MCNP surface (AVI)

Supplementary Movie 5: Magnification observation of a droplet on the MCNP surface during icing & melting cycle (AVI)

Supplementary Figures:

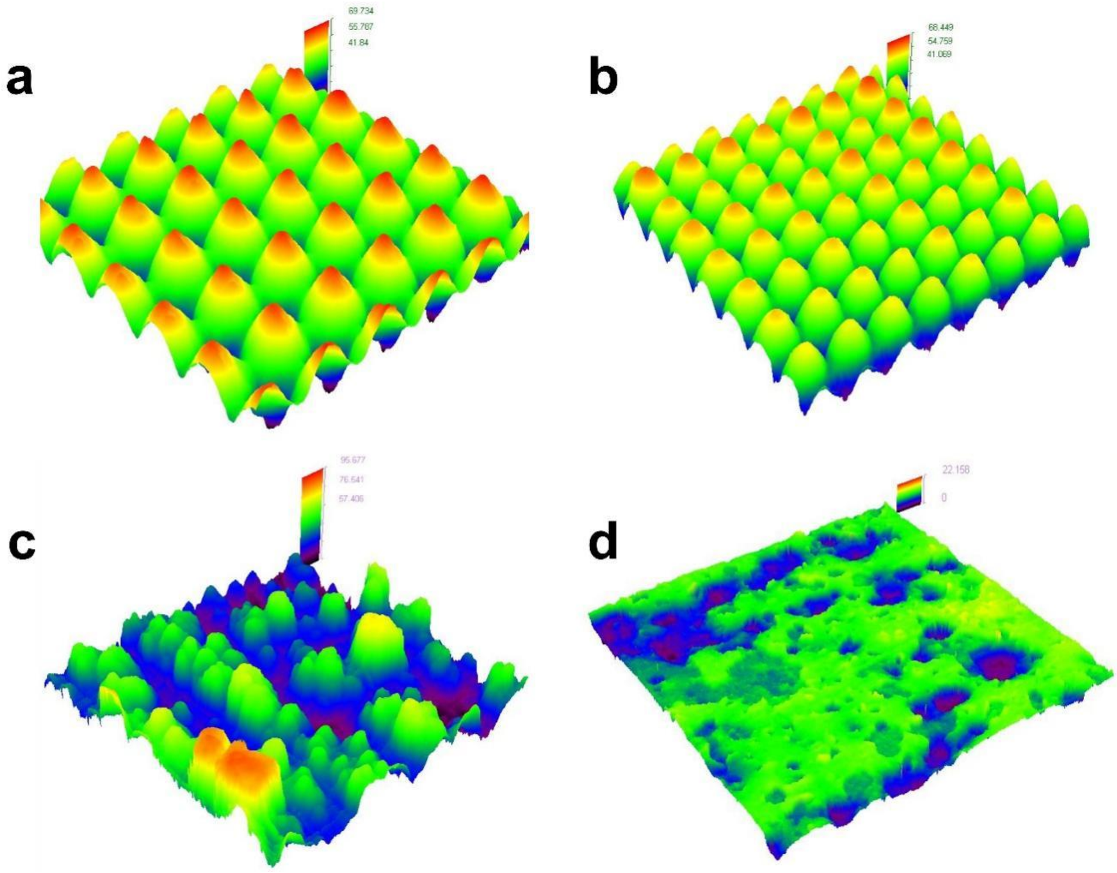

Supplementary Figure 1. 3D topologies of four types of micro-nanostructure. (a) MCNP, (b) SMC, (c) MBNP and (d) IMN.

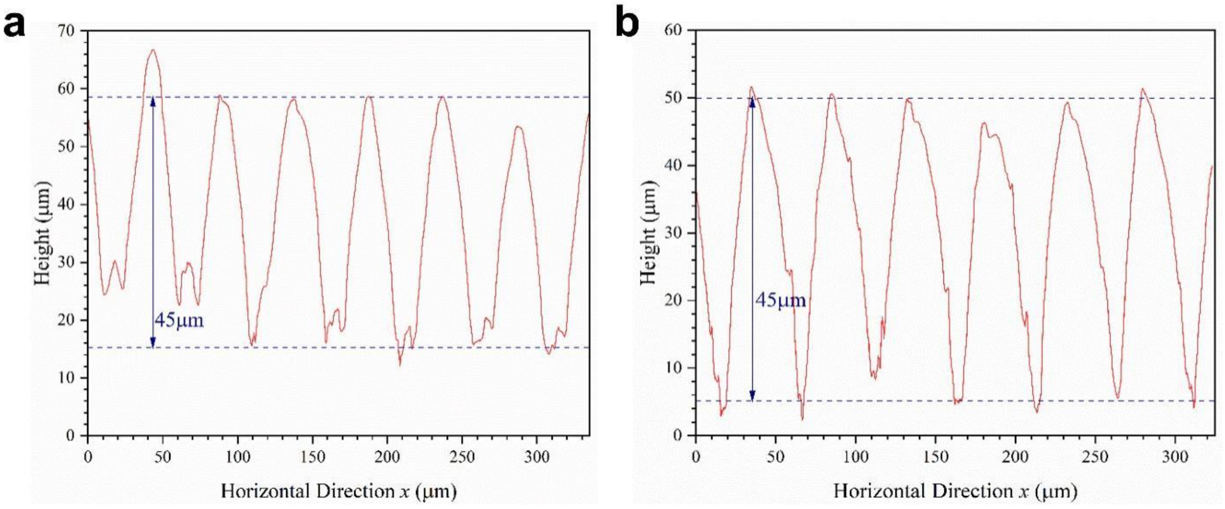

Supplementary Figure 2. The section profiles of MCNP (a) and SMC (b).

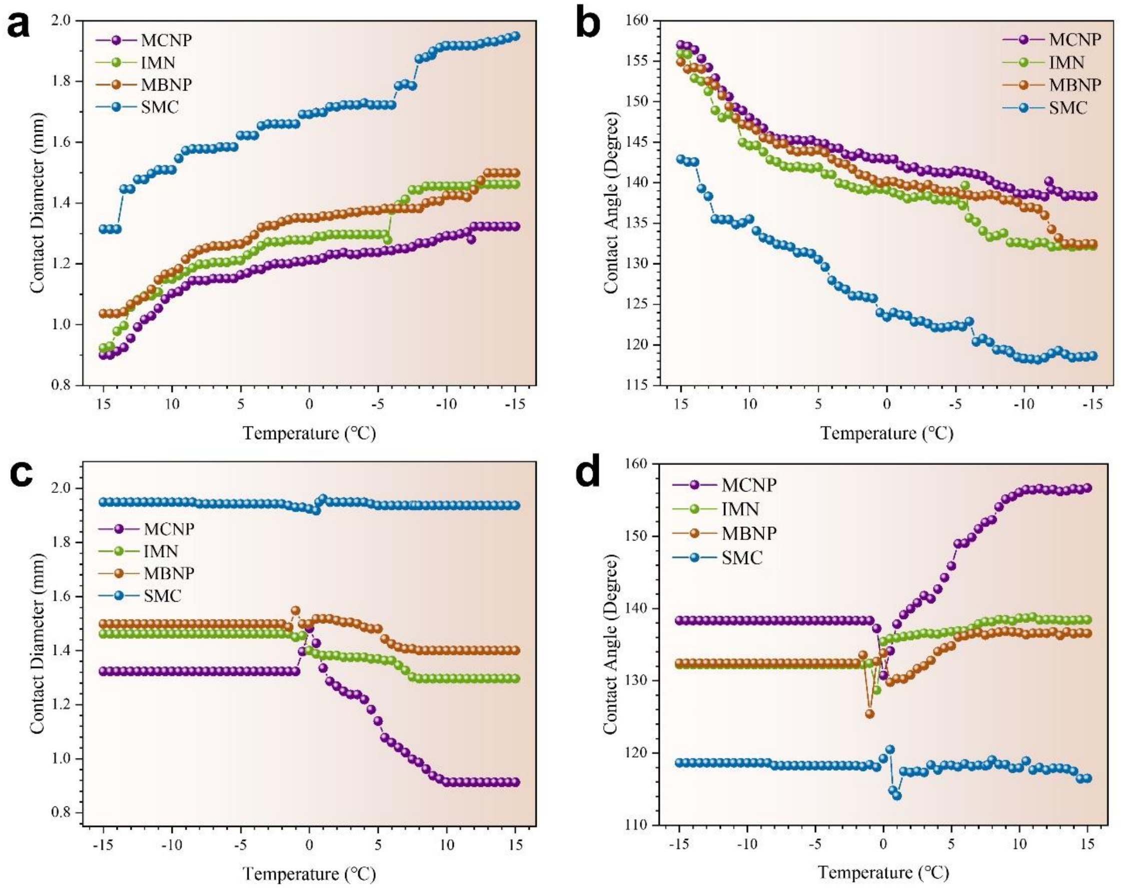

Supplementary Figure 3. Comparison of droplet state changes on four types of surfaces during icing & melting cycle. (a) and (b) The changes of contact diameters and angles of droplets on the four surfaces during icing

process. (c) and (d) The changes of contact diameters and angles of droplets on the four surfaces during melting process.

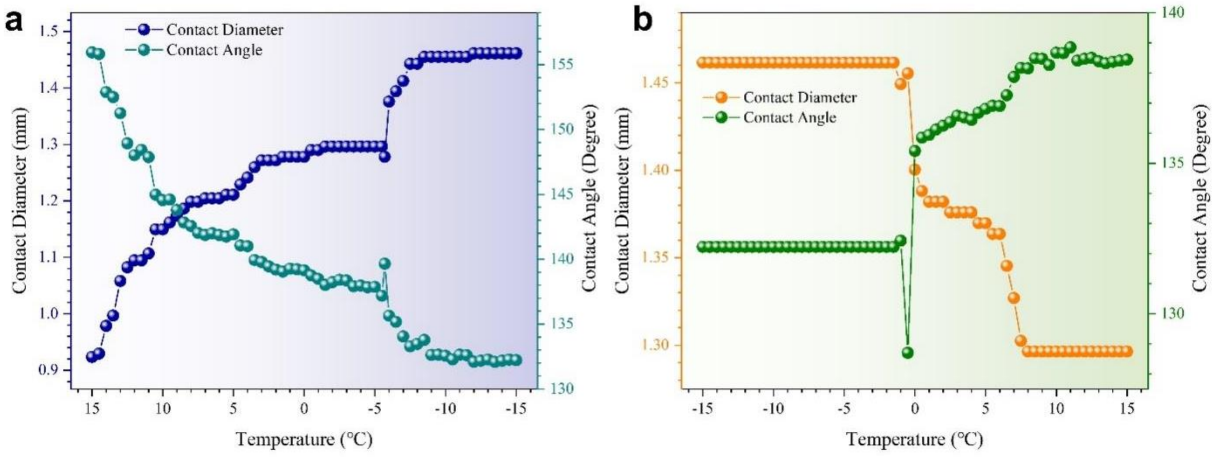

Supplementary Figure 4. The change of contact diameters and contact angles of droplets on the IMN surface during the icing and melting processes. (a) Icing process. (b) Melting process.

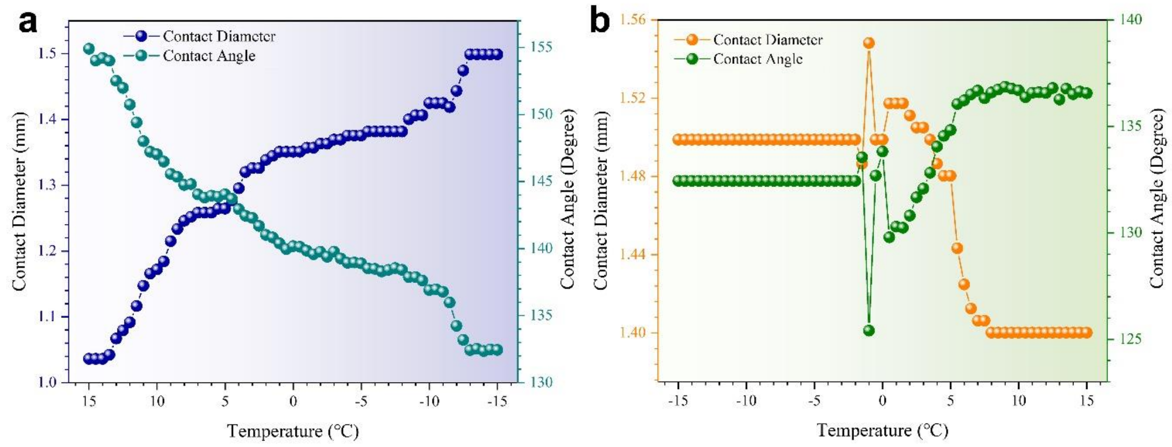

Supplementary Figure 5. The change of contact diameters and contact angles of droplets on the MBNP surface during the icing and melting processes. (a) Icing process. (b) Melting process.

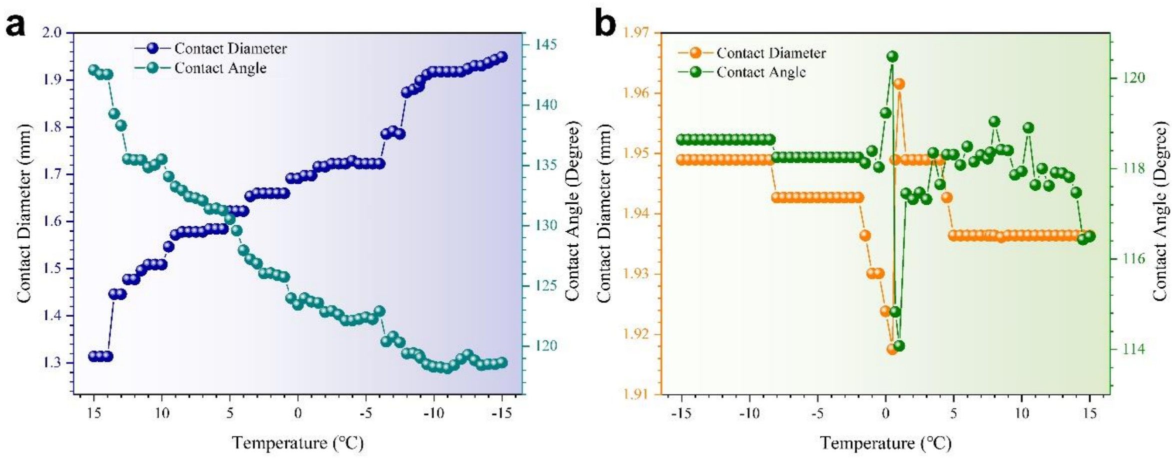

Supplementary Figure 6. The change of contact diameters and contact angles of droplets on the SMC surface during the icing and melting processes. (a) Icing process. (b) Melting process.

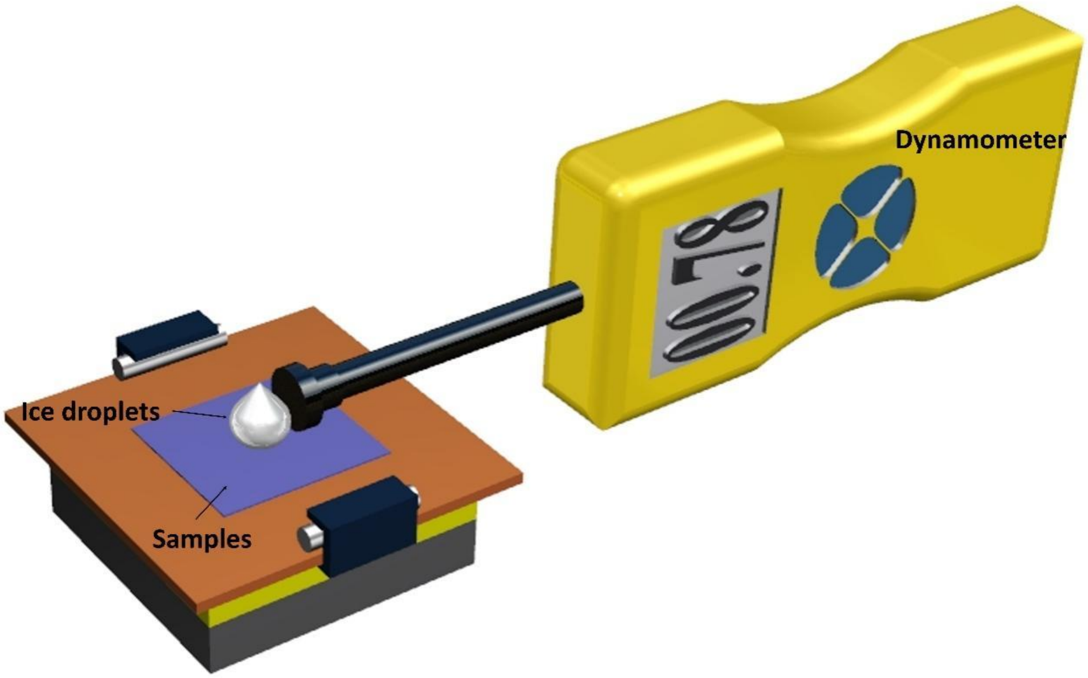

Supplementary Figure 7. The schematic for measuring the icing adhesion strength.

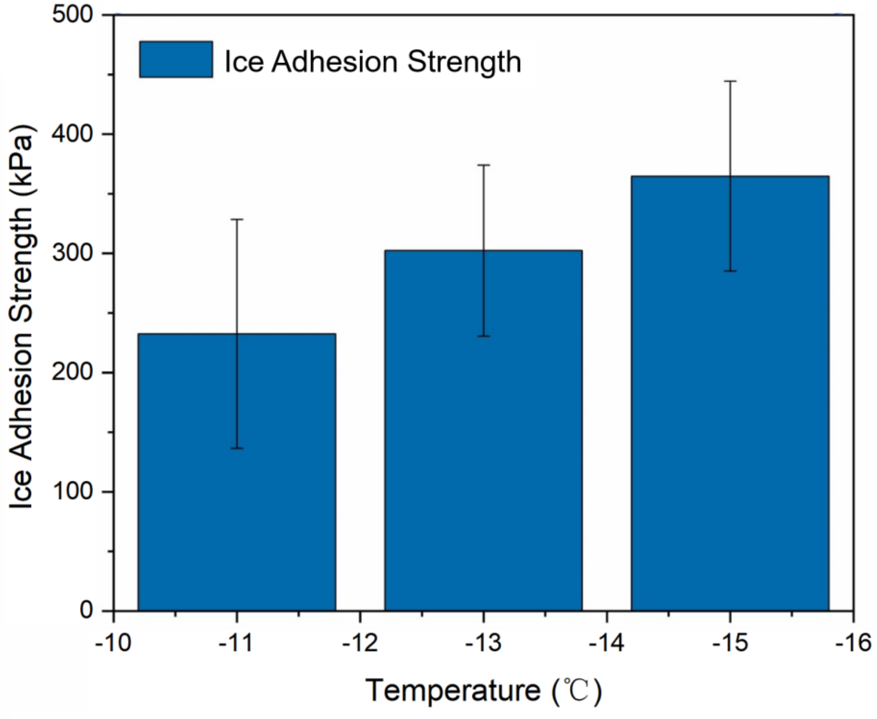

Supplementary Figure 8. The schematic for measuring the icing adhesion strength. Data are mean ± s.d. from at least three independent measurements.

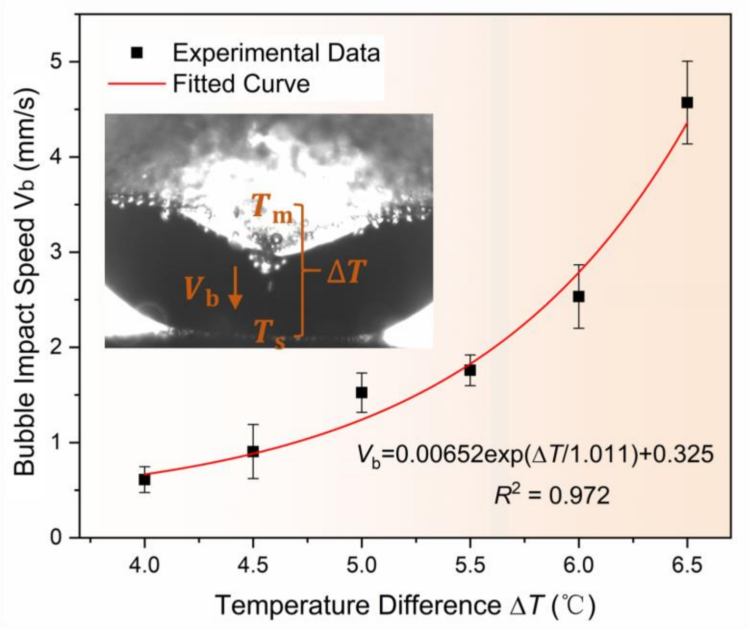

Supplementary Figure 9. The change of bubble impact speed V_b with the increase of temperature difference. The red line presents the fitted curve, which can be expressed as $V_{\mathrm{b}} = 0.00652\exp\left(\frac{\Delta T}{1.011}\right) + 0.325$, where V_{b} is bubbles speed, ΔT is the temperature difference between the melting zone and non-melting zone of the ice droplet. Experimental picture is inserted in the graph. Temperatures, bubble speed and moving directions are marked, where T_{m} and T_{s} represents the temperature of melting front and substrate, respectively. Data are mean ± s.d. from at least three independent measurements.

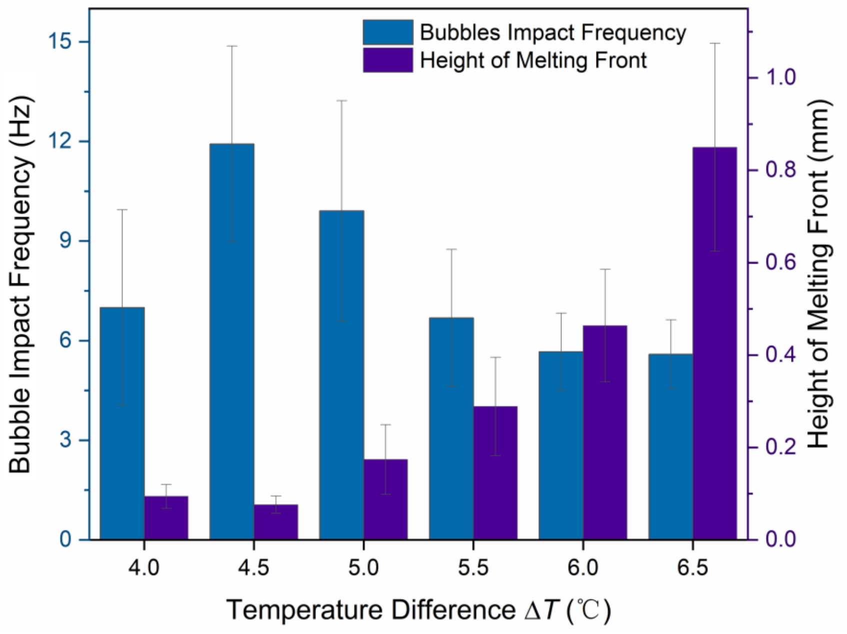

Supplementary Figure 10. The changes of the bubble impact frequency F_{bi} and melting front heights with the increase of temperature difference. The bubbles impact frequency F_{bi} describes the bubbles flux at different temperatures. It can be calculated by $F_{bi} = V_b/H_m$, where H_m is the height of the melting front, which is measured from the substrate to the lowest point of the melting front. Data are mean ± s.d. from at least three independent measurements.

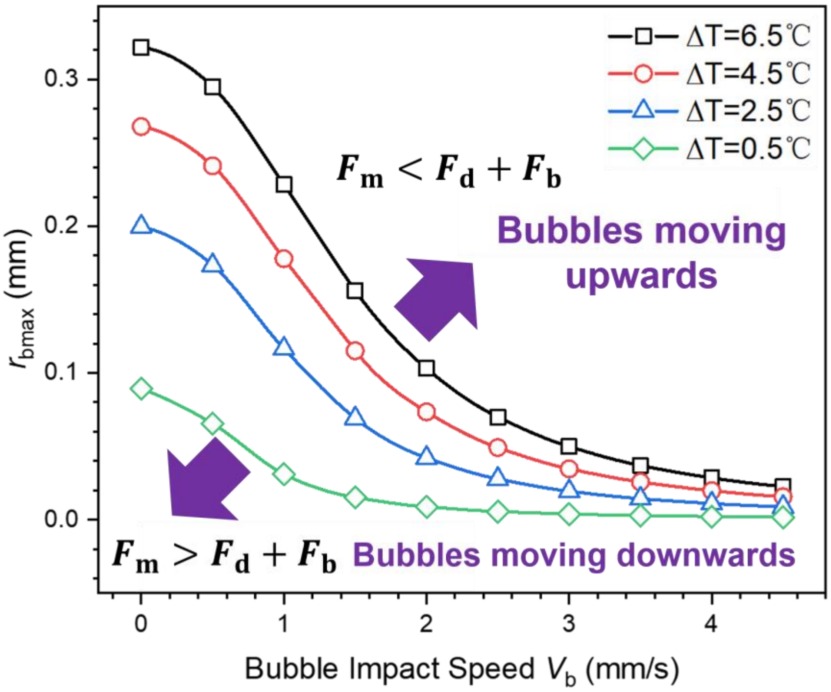

Supplementary Figure 11. Critical maximum bubble radius for moving downwards under different temperature differences. The critical moving equations and critical moving scopes are marked. When the bubble radius is larger than the critical maximum radius r_{bmax}, the bubble moves upwards instead of downwards.

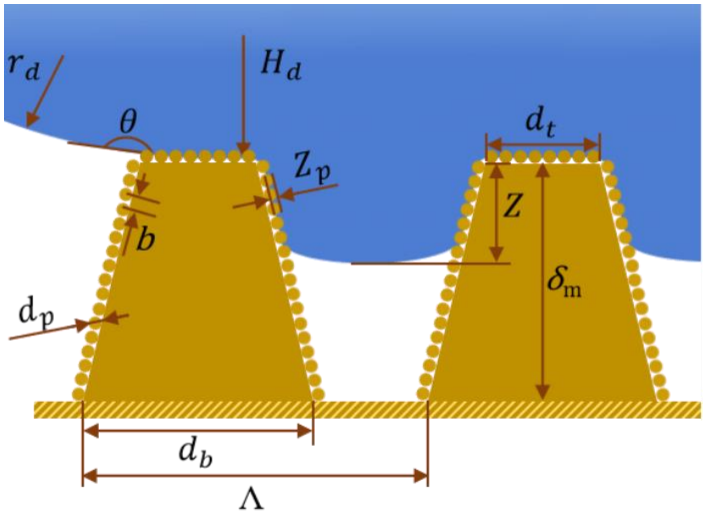

Supplementary Figure 12. Theoretical analysis model of different wetting modes.

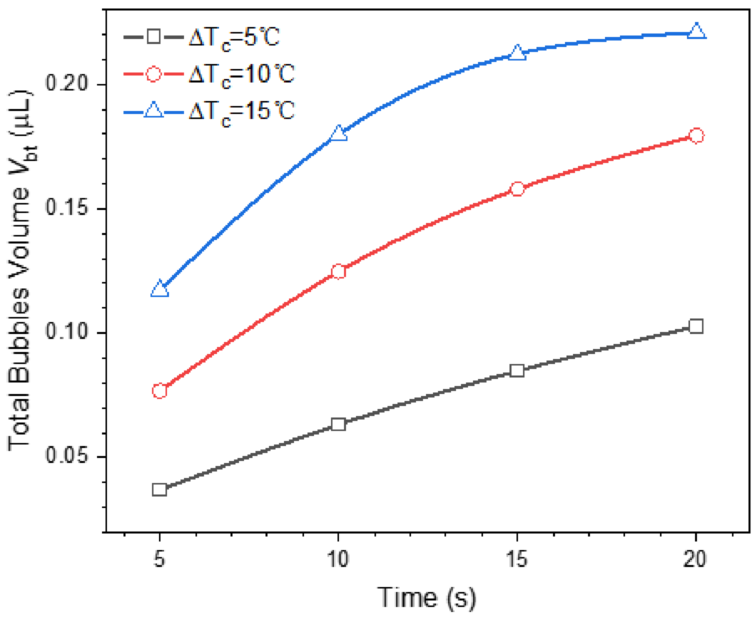

Supplementary Figure 13. The total bubbles volume frozen in the ice droplets under different supercooled temperatures.

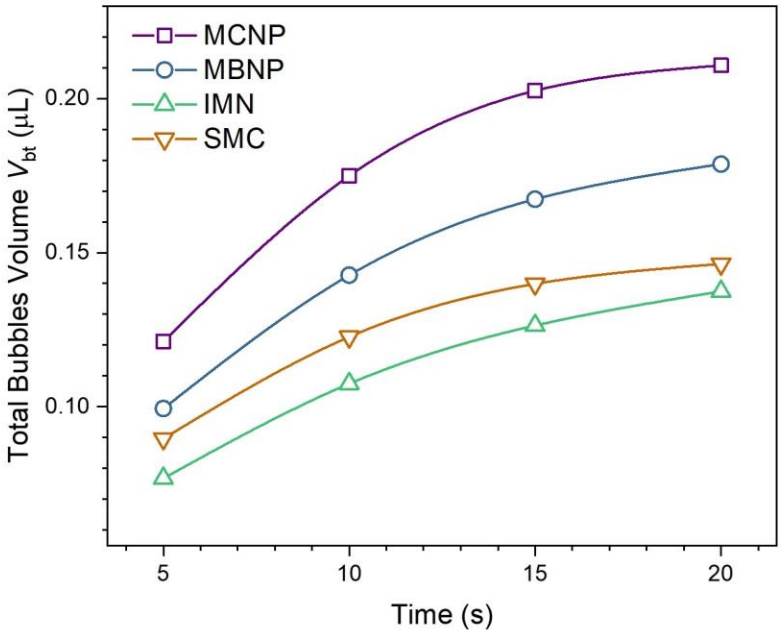

Supplementary Figure 14. The total bubbles volume frozen in the ice droplets on four hydrophobic surfaces.

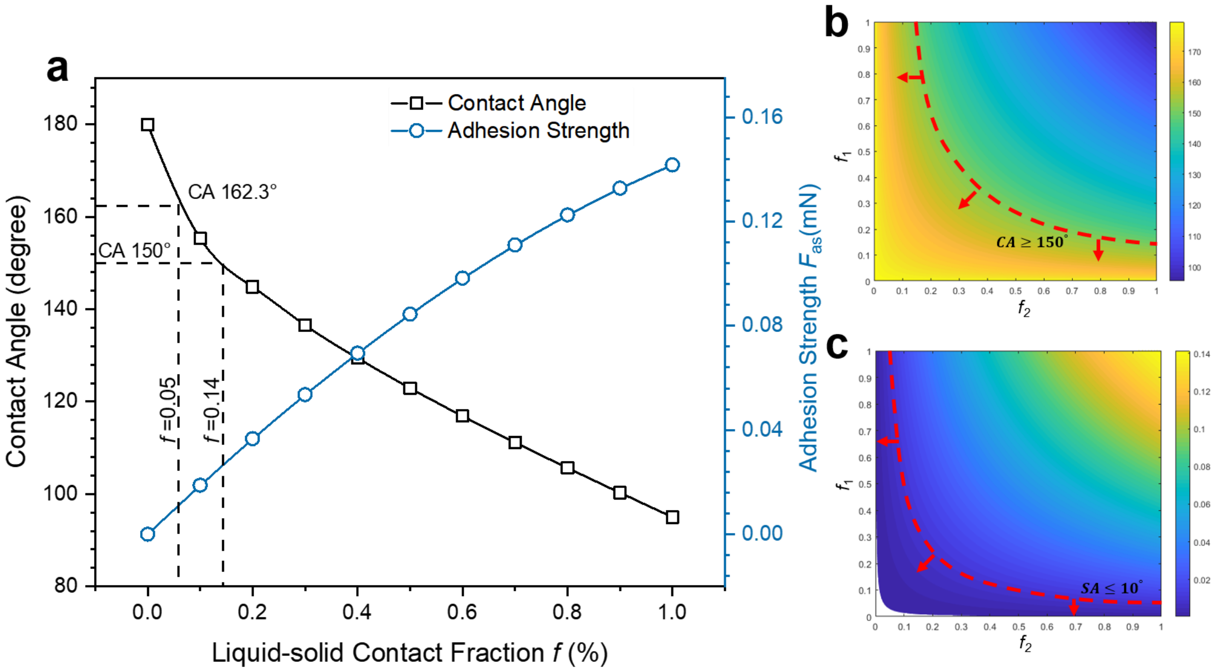

Supplementary Figure 15. Contact angles and adhesion strength (sliding angles) under different liquid-solid contact fractions. (a) Contact angles. (b) Sliding angles.

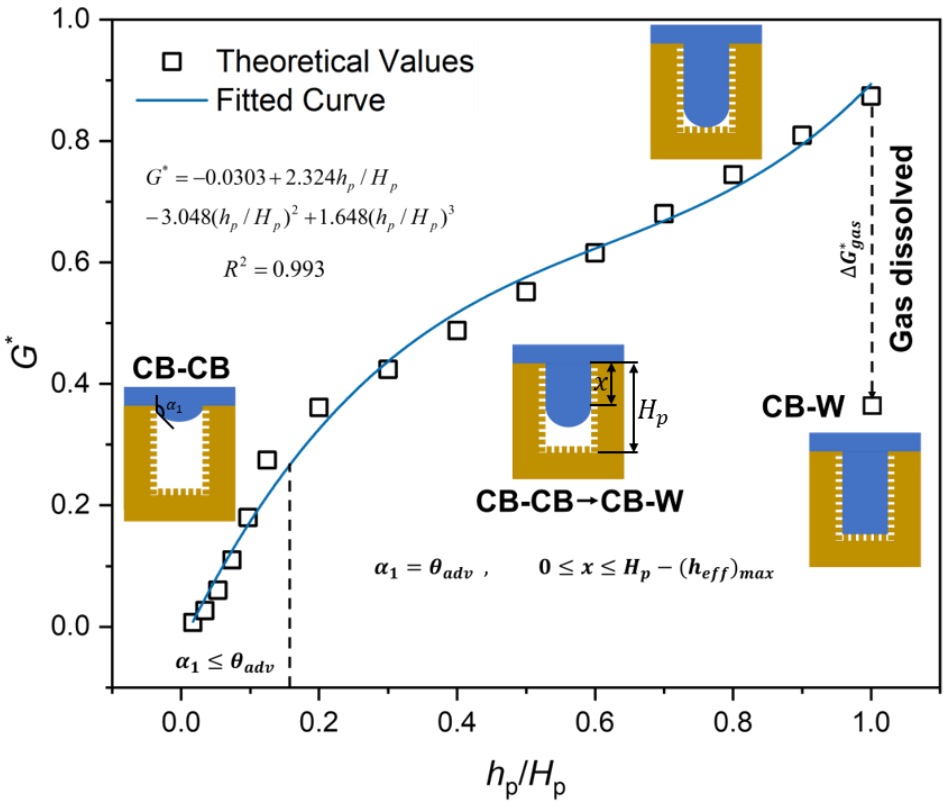

Supplementary Figure 16. The changes of the system free energy with the increase of the penetration depth h_p/H_p.

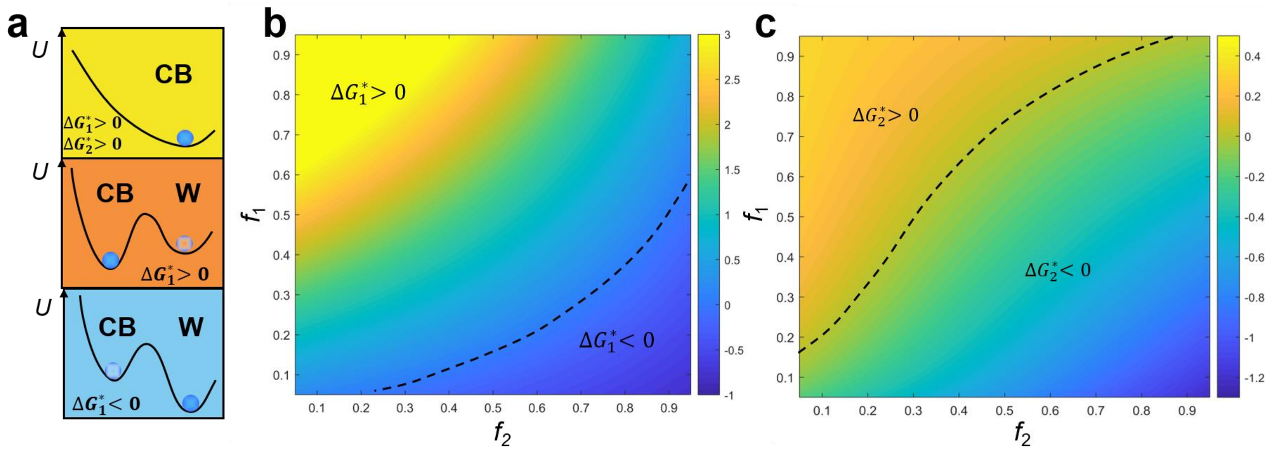

Supplementary Figure 17. Gibbs free energy phase diagram under different system energy states. (a) Three system energy states. (b) The distribution of ΔG_1^* on the different f_1 and f_2. (c) The distribution of ΔG_2^* on the different f_1 and f_2.

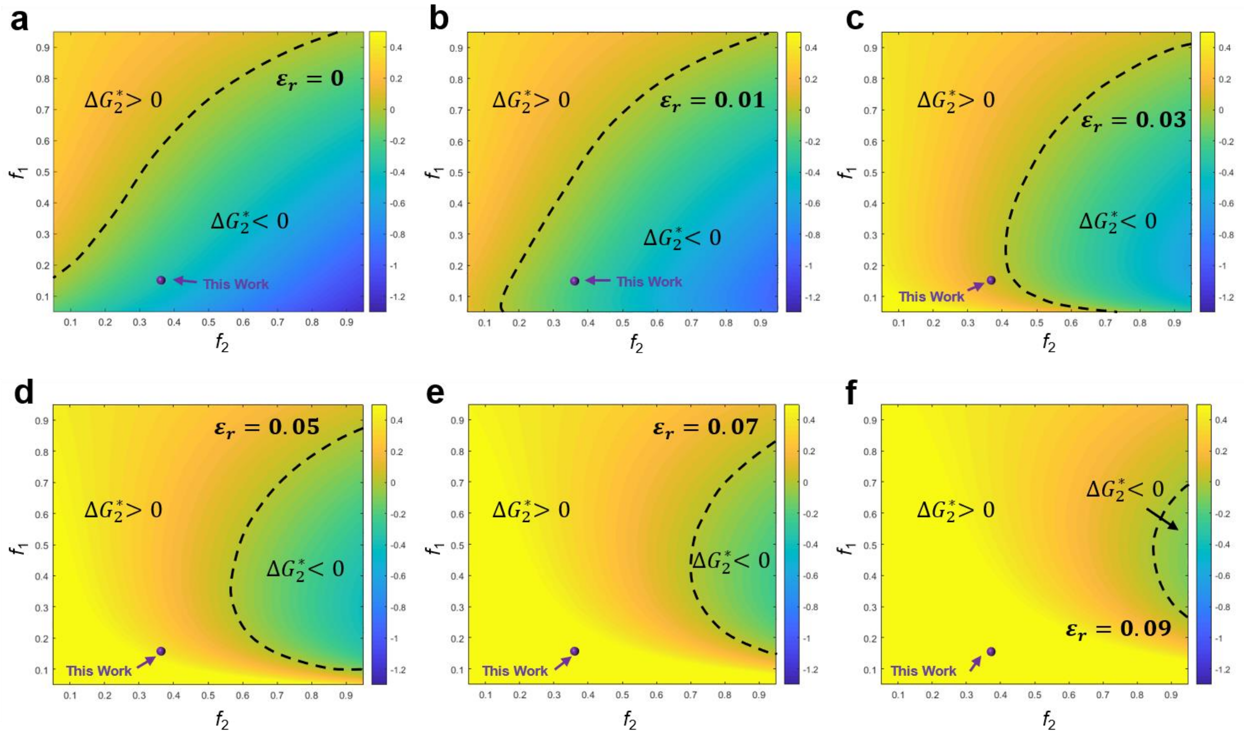

Supplementary Figure 18. Phase diagram of ΔG_2^* under different recovery factors ε_r. (a) $\varepsilon_r = 0$. (b) $\varepsilon_r = 0.01$. (c) $\varepsilon_r = 0.03$. (d) $\varepsilon_r = 0.05$. (e) $\varepsilon_r = 0.07$. (f) $\varepsilon_r = 0.09$.

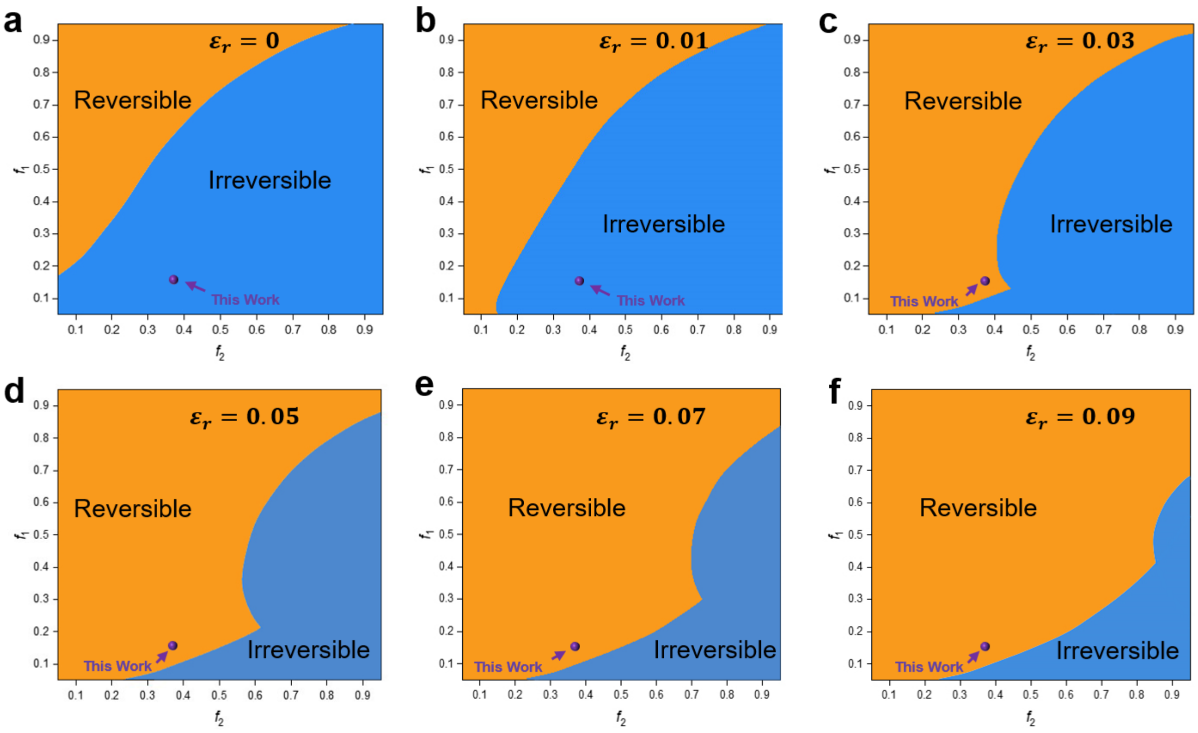

Supplementary Figure 19. Phase diagram of the occurrence of the dewetting transitions. (a) $\varepsilon_r = 0$. (b) $\varepsilon_r = 0.01$. (c) $\varepsilon_r = 0.03$. (d) $\varepsilon_r = 0.05$. (e) $\varepsilon_r = 0.07$. (f) $\varepsilon_r = 0.09$.

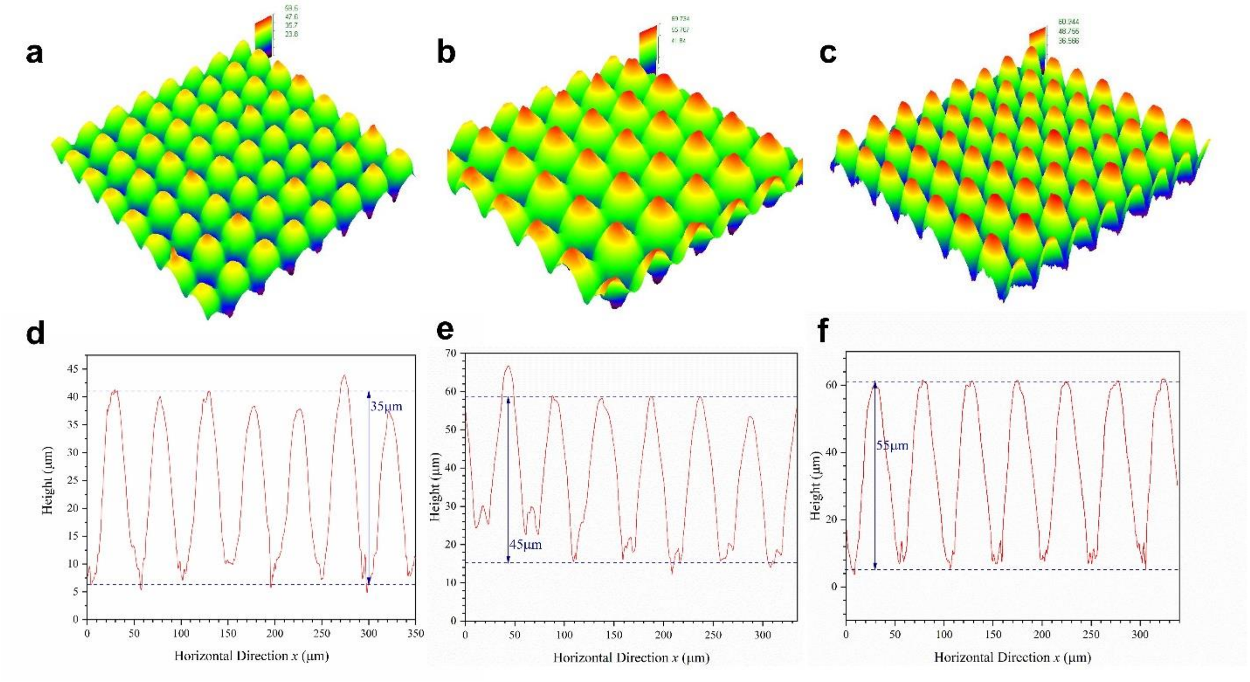

Supplementary Figure 20. Topologies and section view of the MCNP surfaces with different microcones heights. (a, d) H35 P35. (b, e) H45 P35. (c, f) H55 P35.

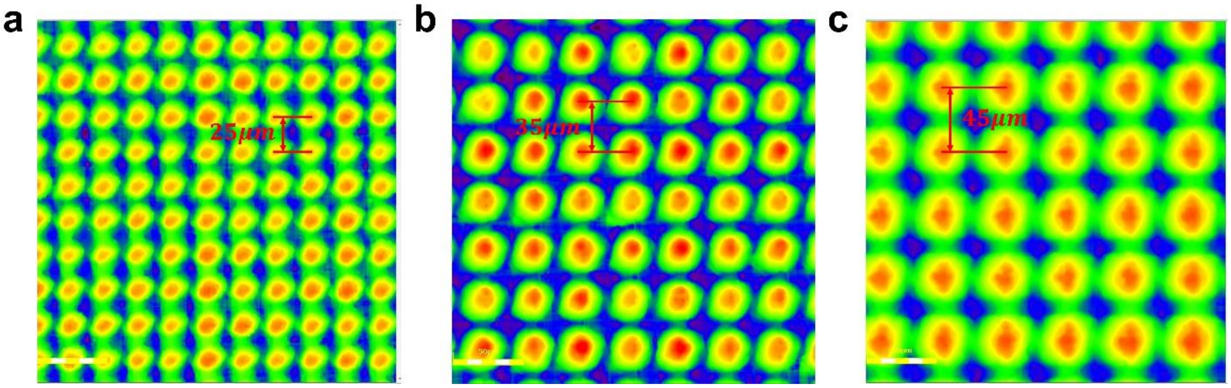

Supplementary Figure 21. Topologies of the MCNP surfaces with different microcones pitches. (a) H45 P25. (b) H45 P35. (c) H45 P45.

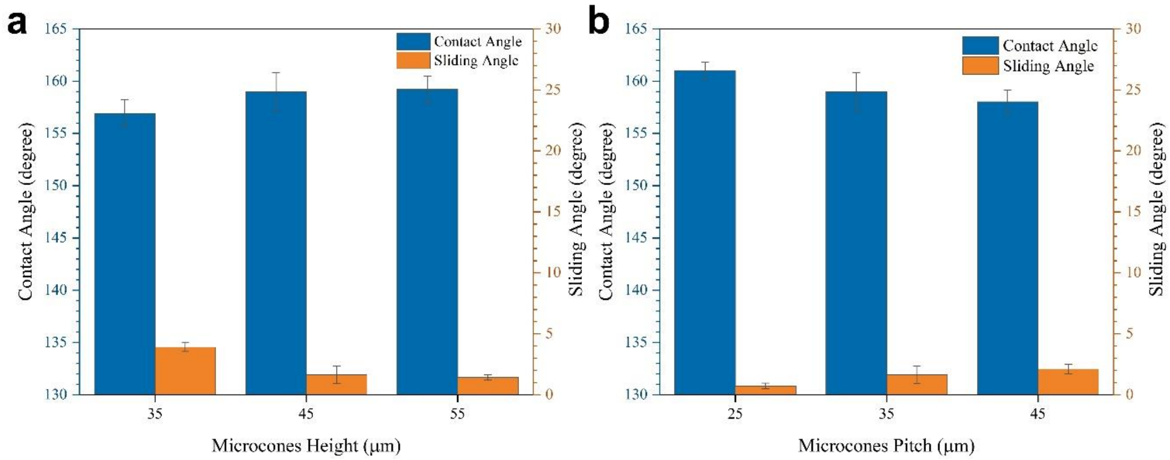

Supplementary Figure 22. Superhydrophobicity of the MCNP surfaces with different microcones sizes. (a) Different microcones heights. (b) Different microcones pitches. Data are mean ± s.d. from at least three independent measurements.

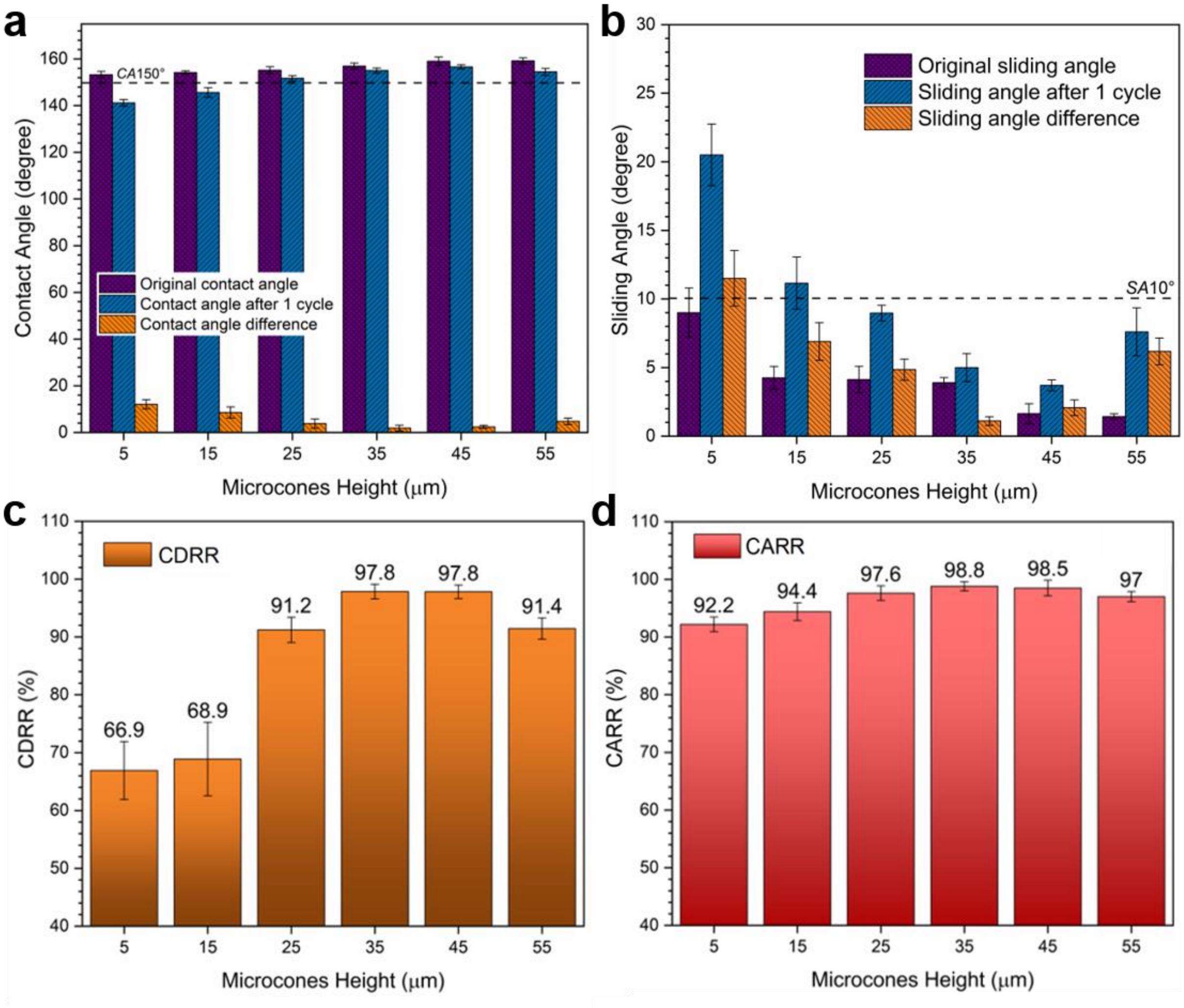

Supplementary Figure 23. Effects of different microcones heights on the dewetting transitions during an icing & melting cycle. (a) Contact angles. (b) Sliding angles. (c) CDRR. (d) CARR. Data are mean ± s.d. from at least three independent measurements.

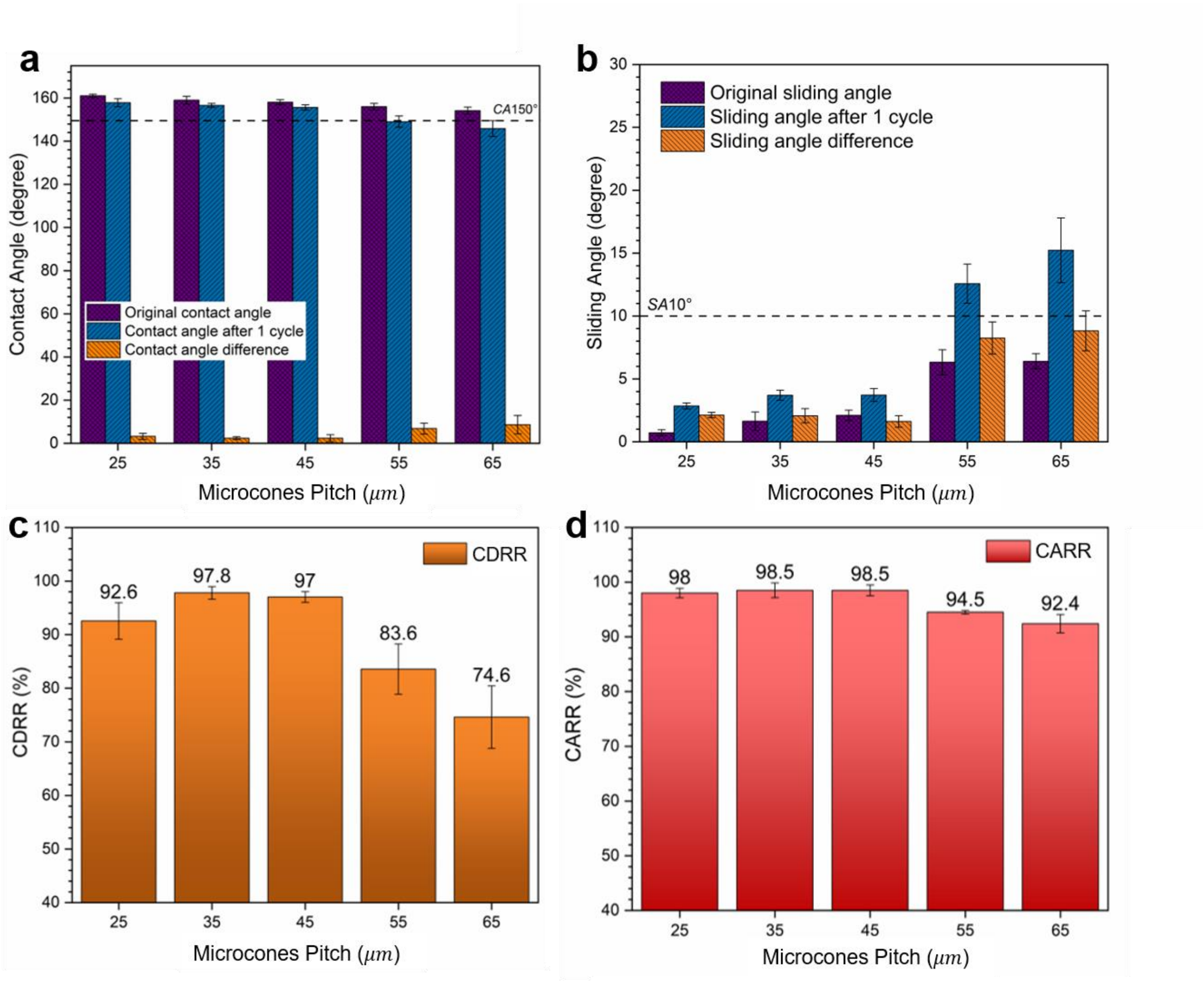

Supplementary Figure 24. Effects of different microcones pitches on the dewetting transitions during an icing & melting cycle. (a) Contact angles. (b) Sliding angles. (c) CDRR. (d) CARR. Data are mean ± s.d. from at least three independent measurements.

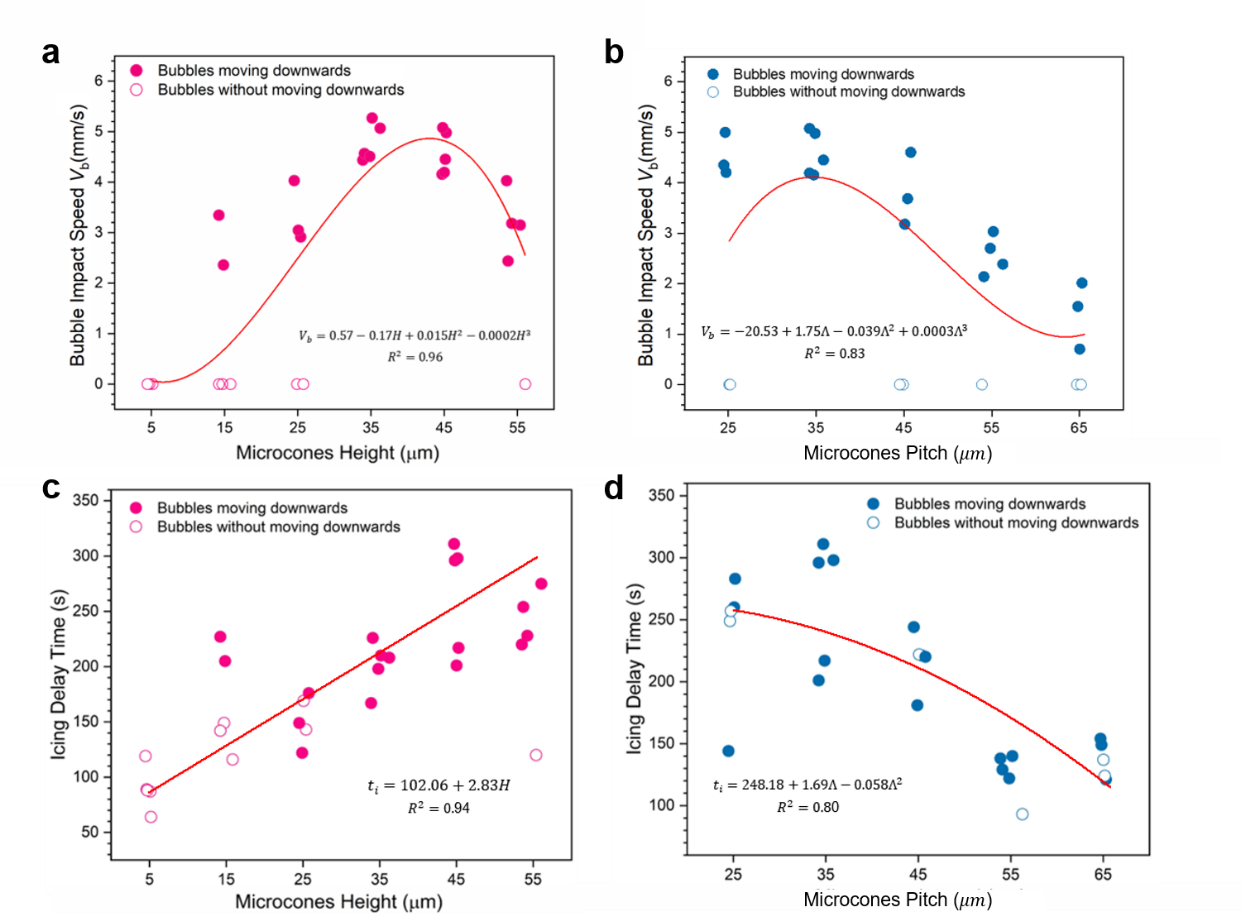

Supplementary Figure 25. Effects of different microcones heights and pitches on the bubble impact speed, icing delay time and the bubble movement. (a) Effects of microcones heights on the bubble impact speed and the bubbles moving directions. (b) Effects of microcones pitches on the bubble impact speed and the bubbles moving directions. (c) Effects of microcones heights on the icing delay time and the bubbles moving directions. (d) Effects of microcones pitches on the icing delay time and the bubbles moving directions. Solid dots represent the bubbles moving downwards while hollow dots represent the bubbles moving upwards. For each micro-nanostructure size, five repeated experiments are conducted. Red lines represent the average value of experimental results.

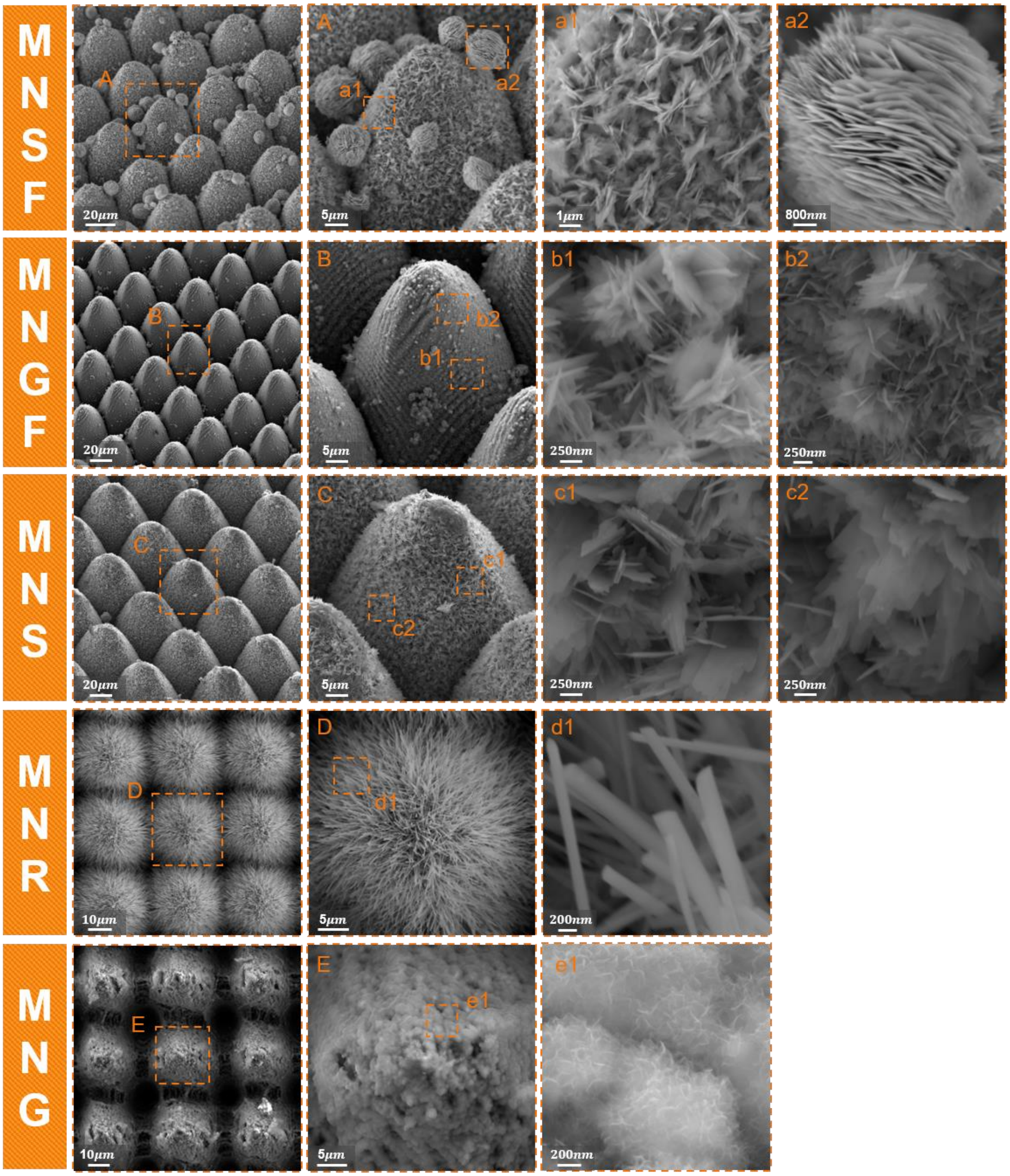

Supplementary Figure 26. SEM images of superhydrophobic surfaces with different micro-nanostructures: MNSF (Microcone arrays covered with dense nanosheets and dispersedly distributed microflowers), MNGF (Microcones covered with dense nanograsses and dispersedly distributed microflowers), MNS (Microcone

arrays covered with dense nanosheets), MNR (Microcone arrays covered with dense nanorods) and MNG (Microcone arrays covered with dense nanograsses).

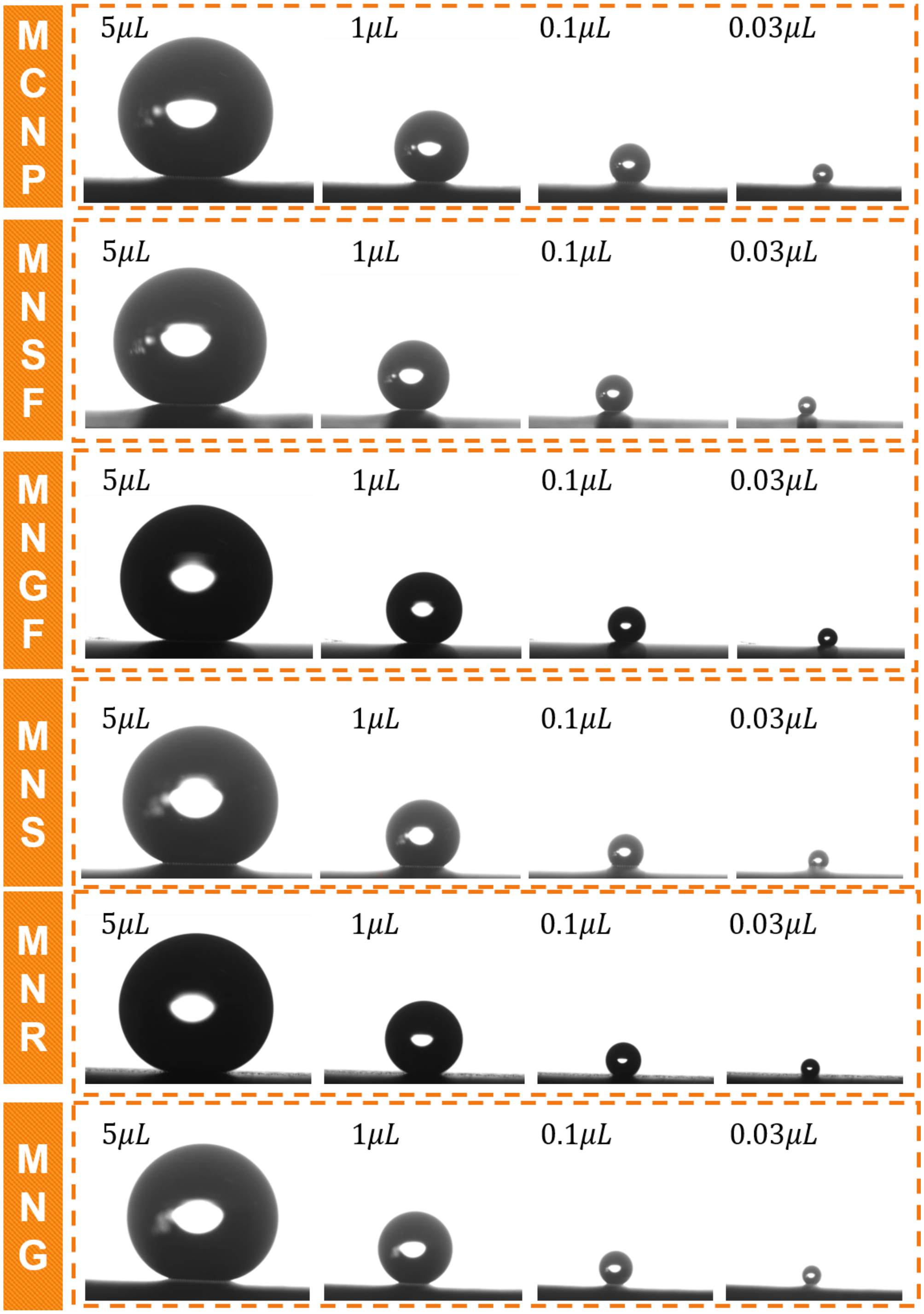

Supplementary Figure 27. The Cassie-Baxter stability experiments for different superhydrophobic surfaces.

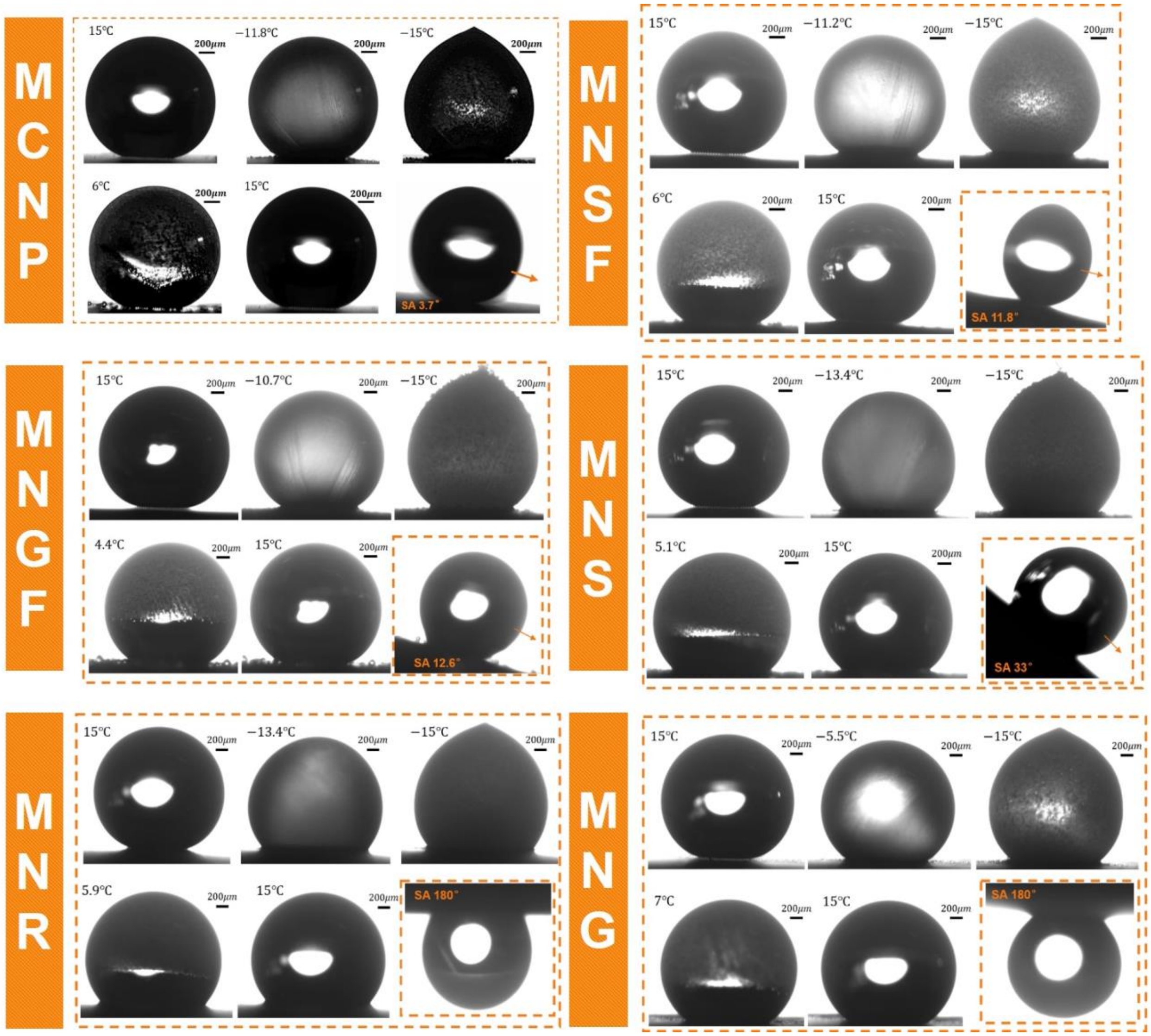

Supplementary Figure 28. The icing & melting processes on different surfaces. The sliding conditions of droplets after melting are also shown for each surface. Sliding angles are marked in the figure.

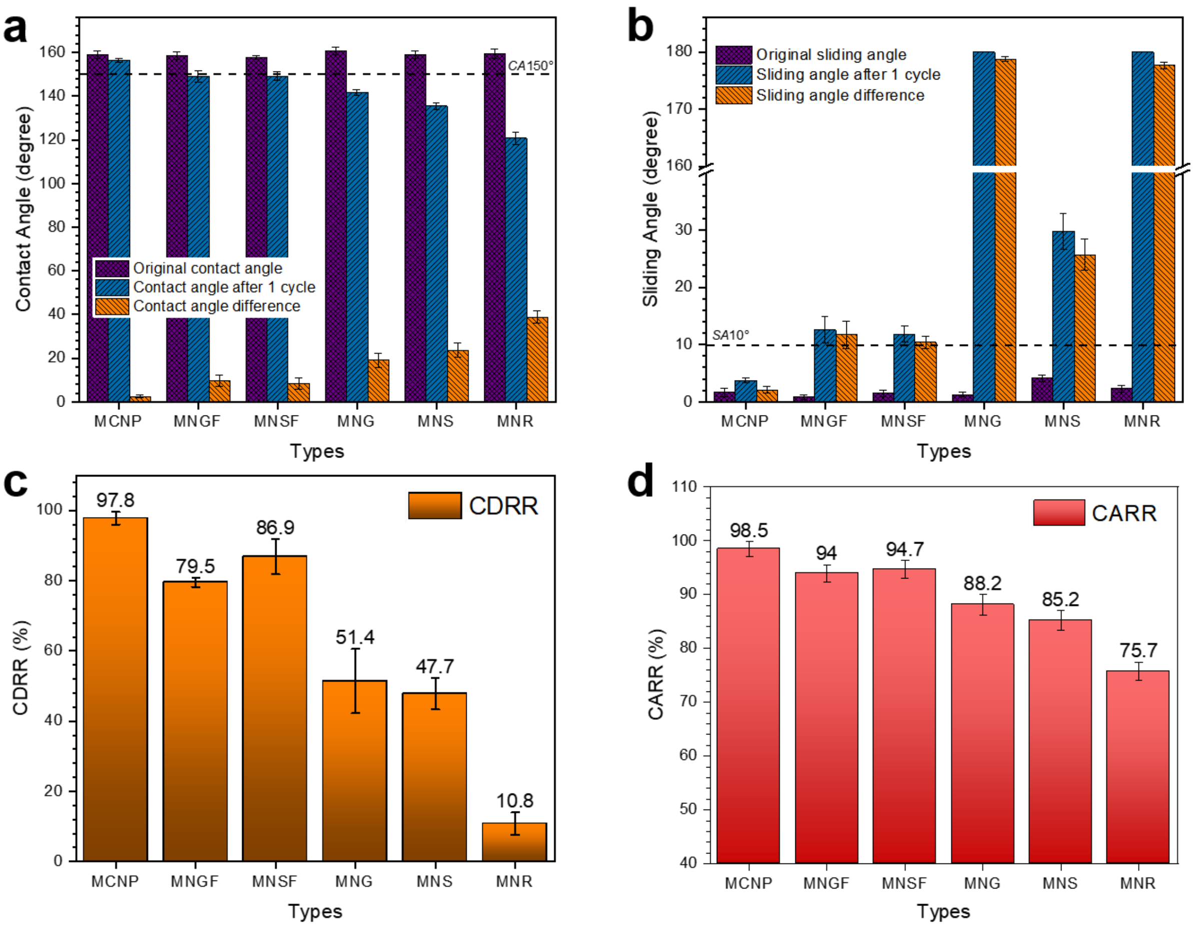

Supplementary Figure 29. The CA, SA, CDRR, CARR changes of droplets on different superhydrophobic surfaces after an icing & melting cycle. Data are mean ± s.d. from at least three independent measurements.

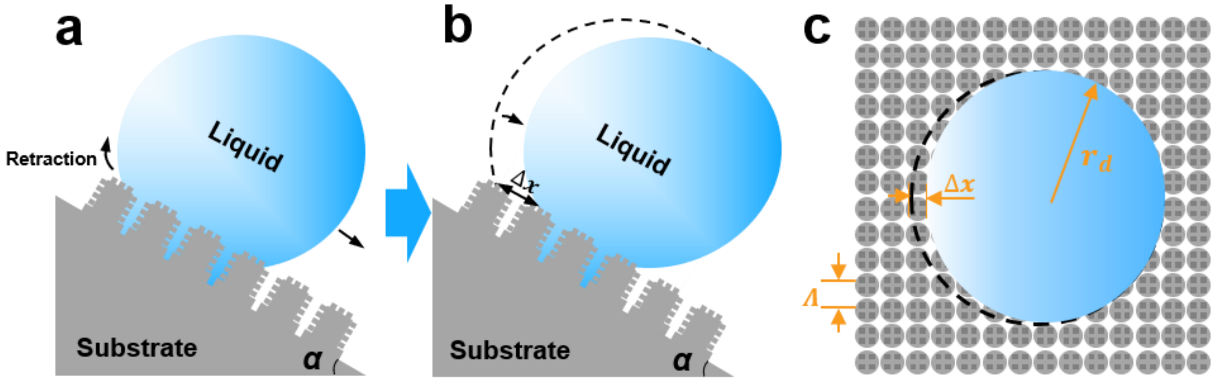

Supplementary Figure 30. Schematics of the model for the surface resistances. (a) Side view of the original state before the droplet sliding. (b) Side view of the final state after droplet sliding. The dashed line denotes the original location of the droplet (c) Top view of the changes of the three-phase-contact-line (TPCL) during the sliding movement.

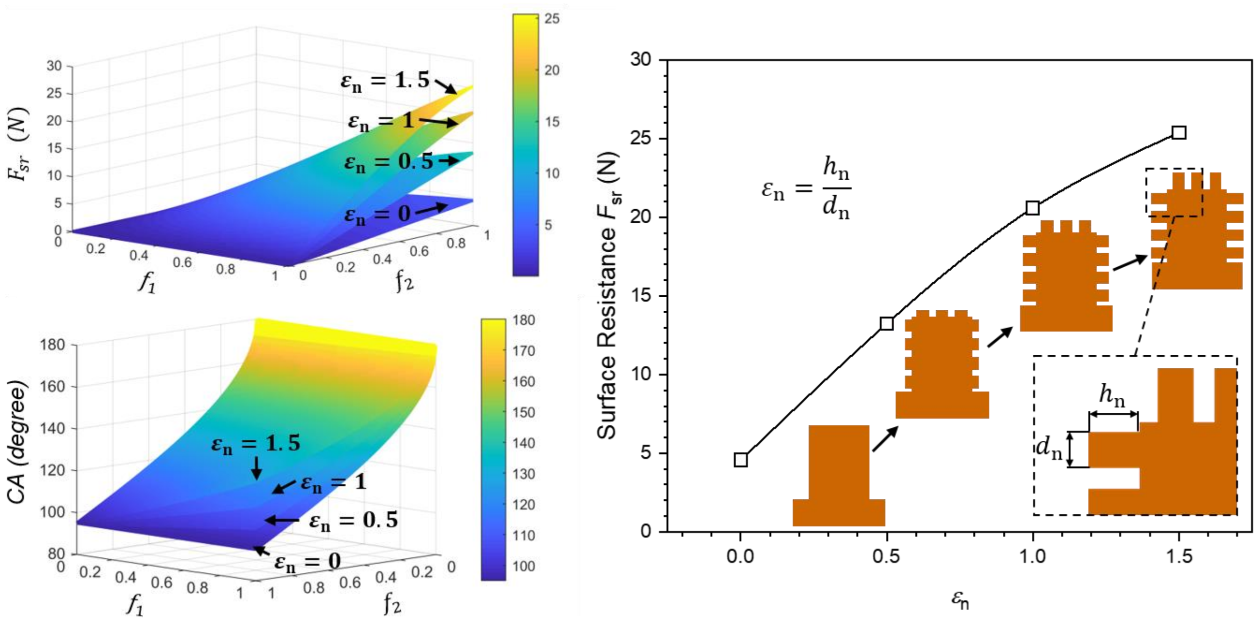

Supplementary Figure 31. Evolution of the contact angles and surface resistances F_{sr} with ε_n. (a) Evolution of F_{sr} with ε_n. (b) Evolution of contact angles with ε_n. (c) Evolution of the maximum surface resistance (when f_1=1, f_2=1) with nanostructures

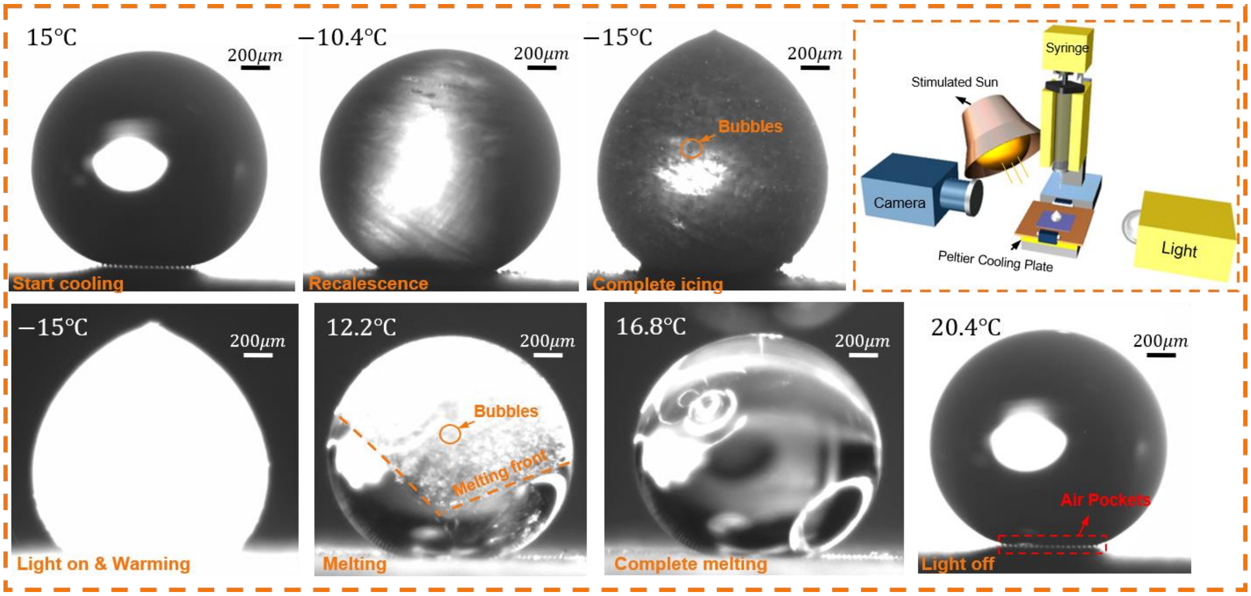

Supplementary Figure 32. Solar-assisted melting experiments on the MCNP surface.

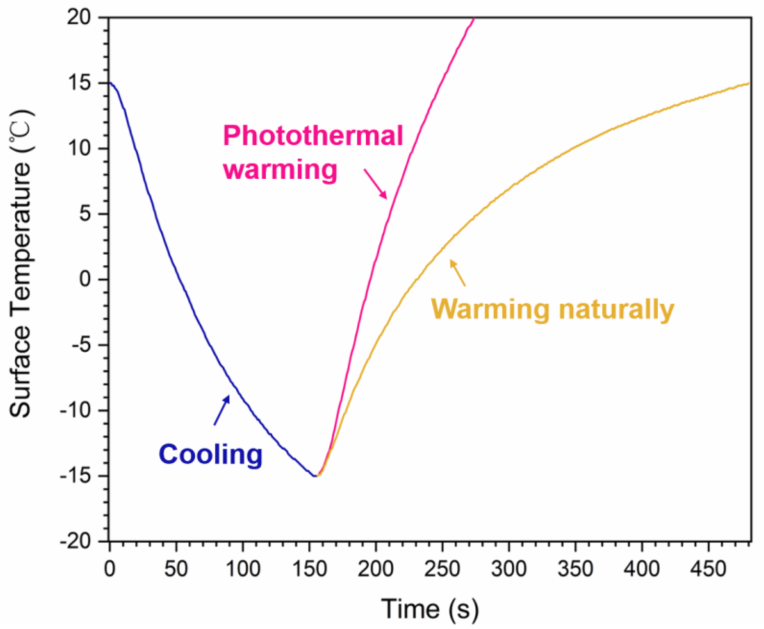

Supplementary Figure 33. Temperature curves of the solar-assisted melting and the natural melting.

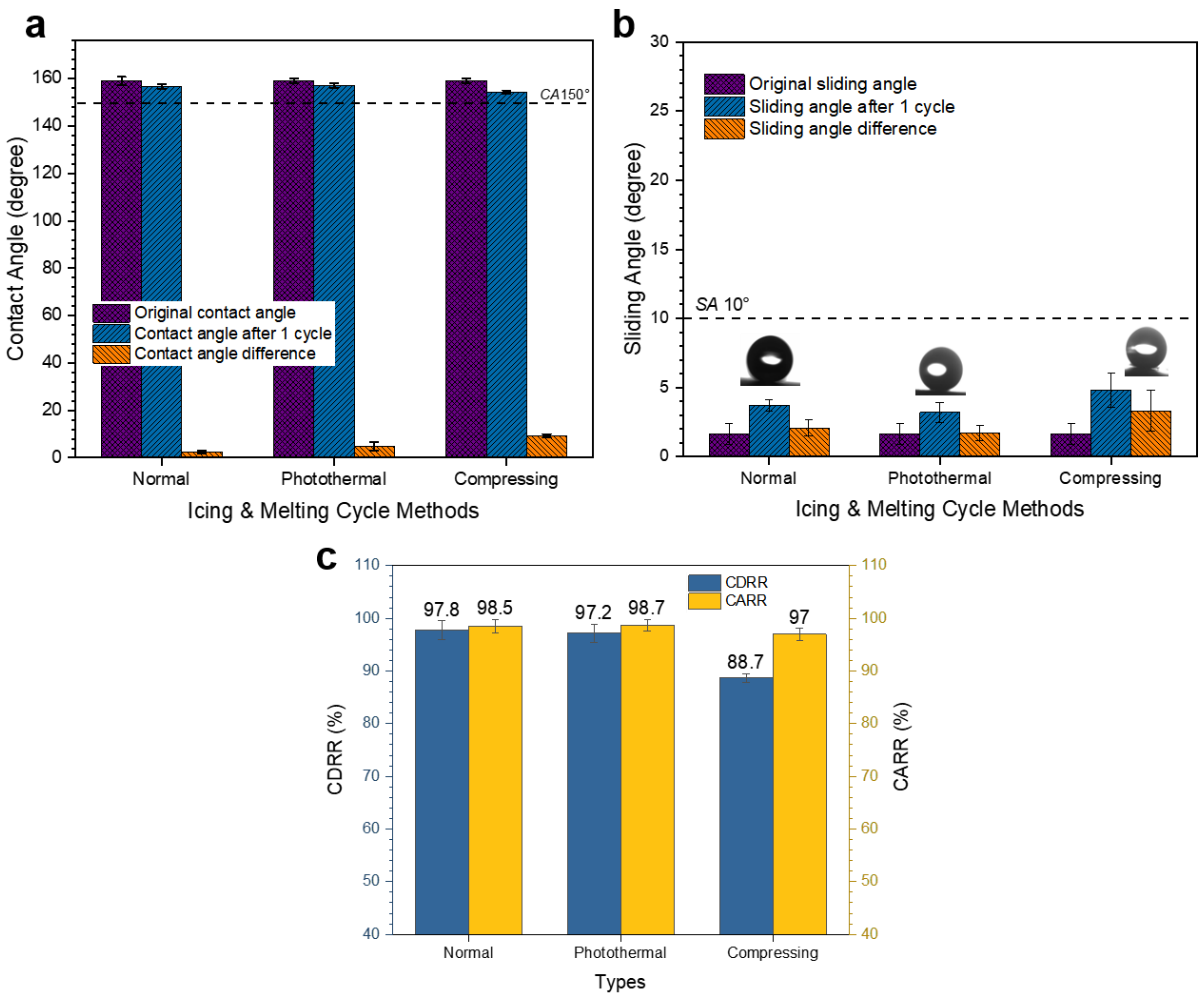

Supplementary Figure 34. Effects of three types of external conditions on the dewetting transitions. Data are mean ± s.d. from at least three independent measurements.

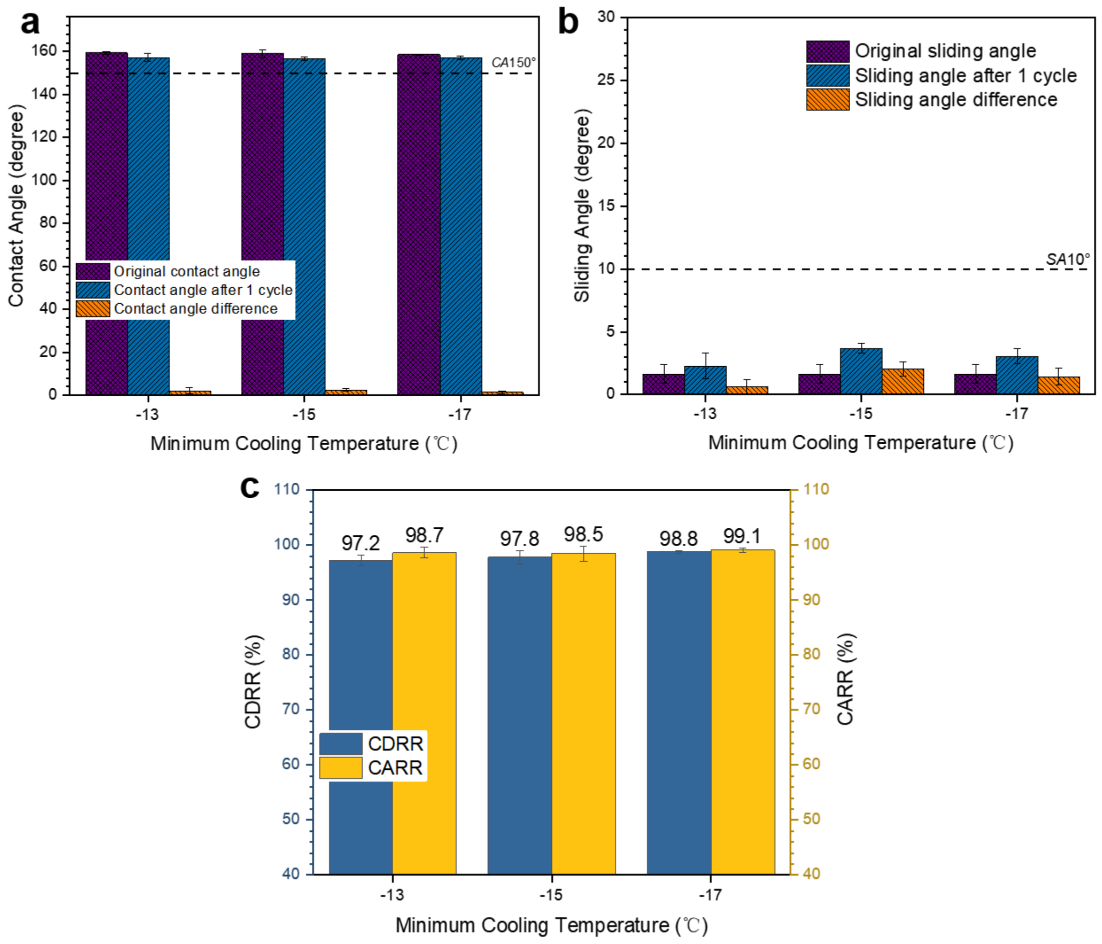

Supplementary Figure 35. Effects of different cooling temperatures on the dewetting transitions. (a) Effects of different cooling temperatures on the contact angles. (b) Effects of different cooling temperatures on the sliding angles. (c) Effects of different cooling temperatures on the CDRR and CARR. Data are mean ± s.d. from at least three independent measurements.

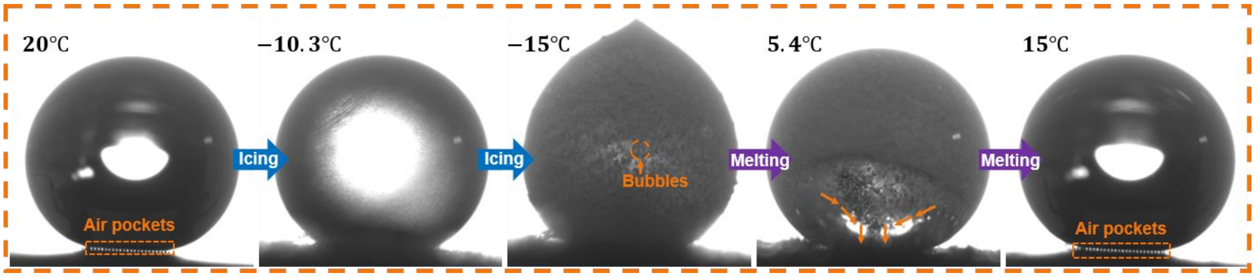

Supplementary Figure 36. Icing & melting tests at room temperature of 20°C.

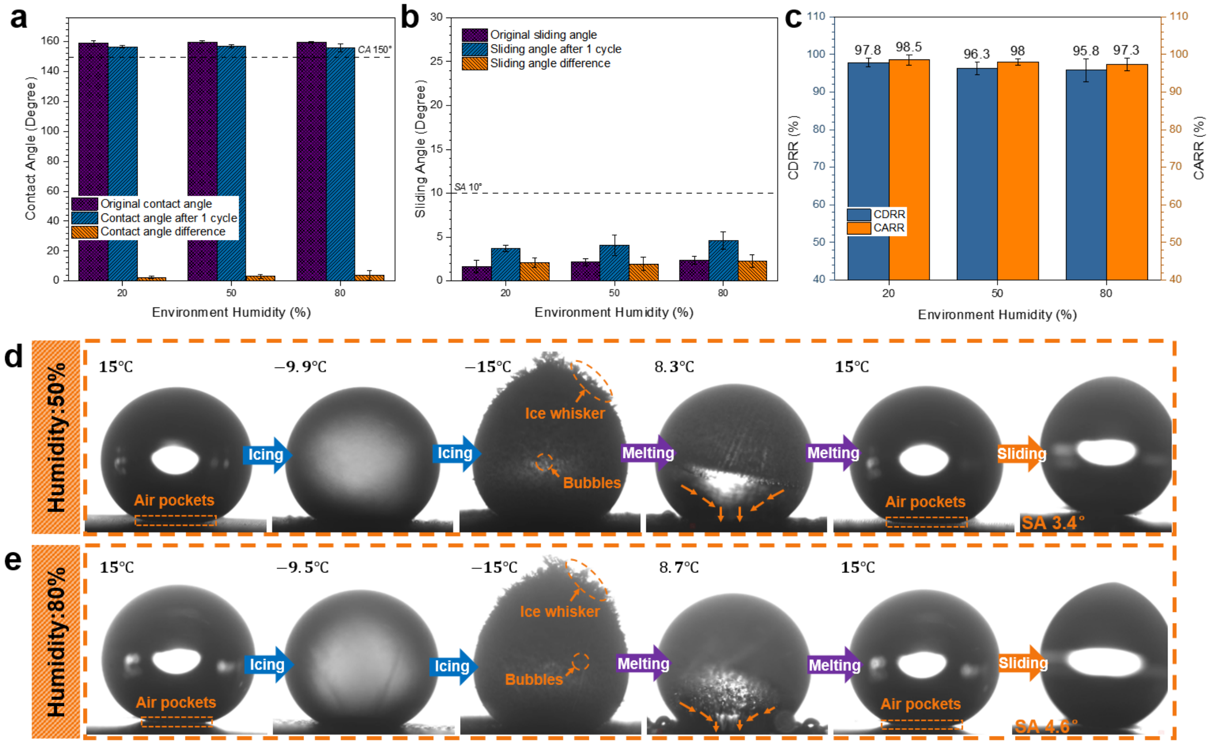

Supplementary Figure 37. Effects of different environmental humidities on the dewetting transitions. (a) Effects on the contact angles. (b) Effects on the sliding angles. (c) Effects on the CDRR and CARR. (d) Icing & melting processes at 50% environment humidity. (e) Icing & melting processes at 80% environment humidity. Ice whiskers, bubbles and air pockets are marked with orange dashed boxes. Sliding angles after melting are also indicated. Data are mean ± s.d. from at least three independent measurements.

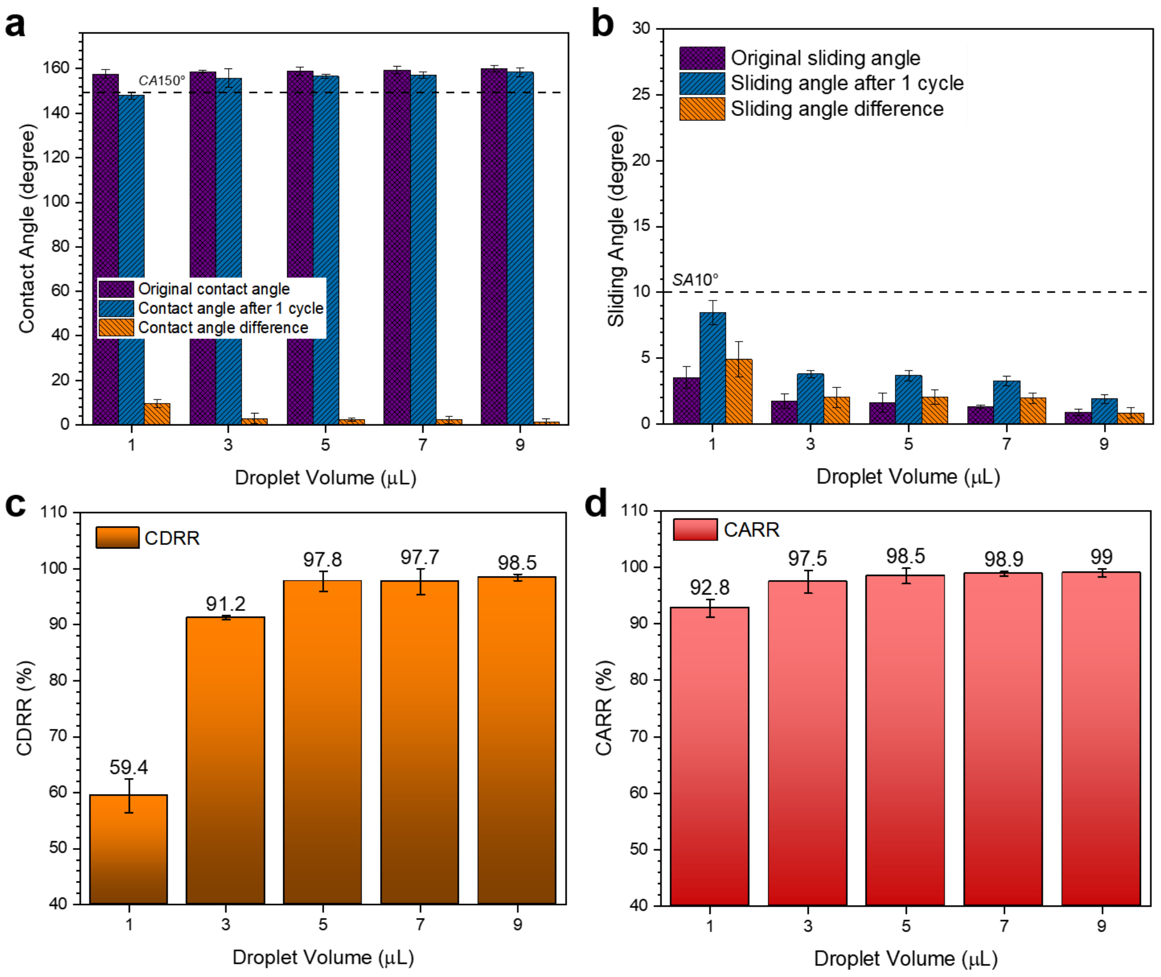

Supplementary Figure 38. Effects of droplets with different volumes on the dewetting transitions. (a) Effects on the contact angles. (b) Effects on the sliding angles. (c) Effects on the CDRR. (d) Effects on the CARR. Data are mean ± s.d. from at least three independent measurements.

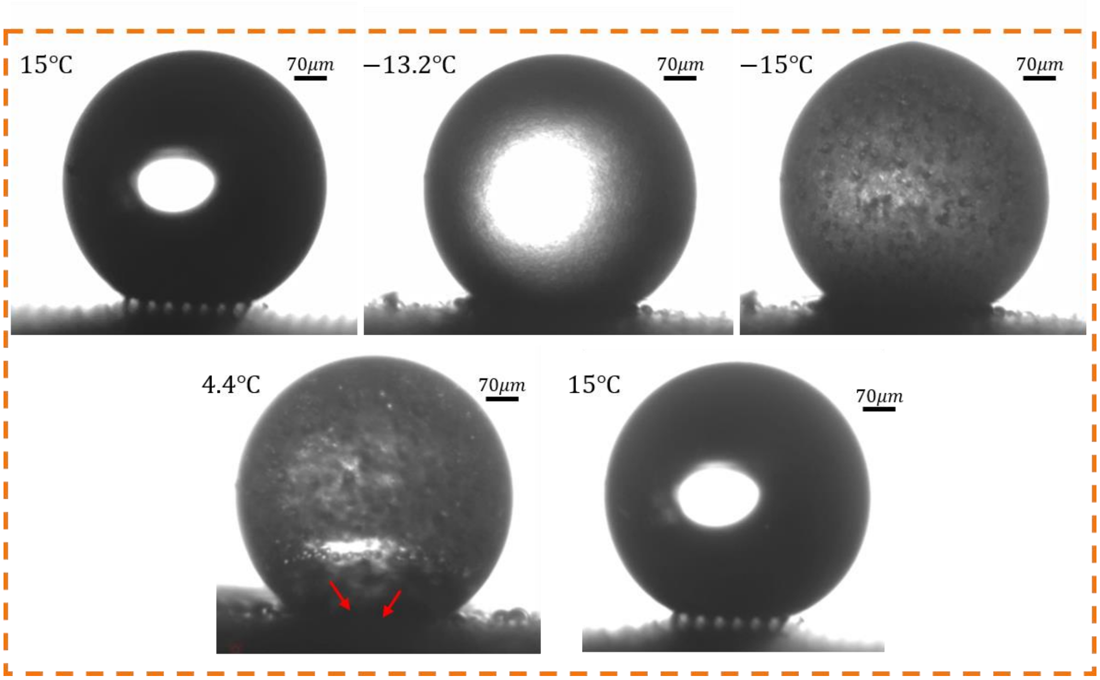

Supplementary Figure 39. Icing & melting cycle for the 50nL droplet.

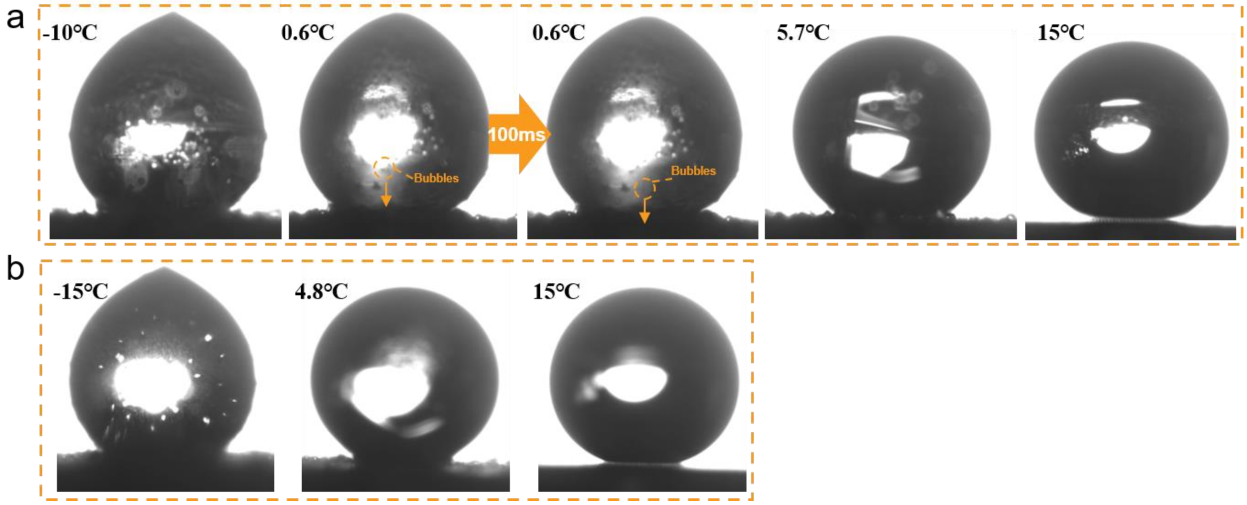

Supplementary Figure 40. Glaze ice formed in different surface temperatures. (a) −10°C. (b) −15°C. Bubbles and their moving directions are marked in the figure.

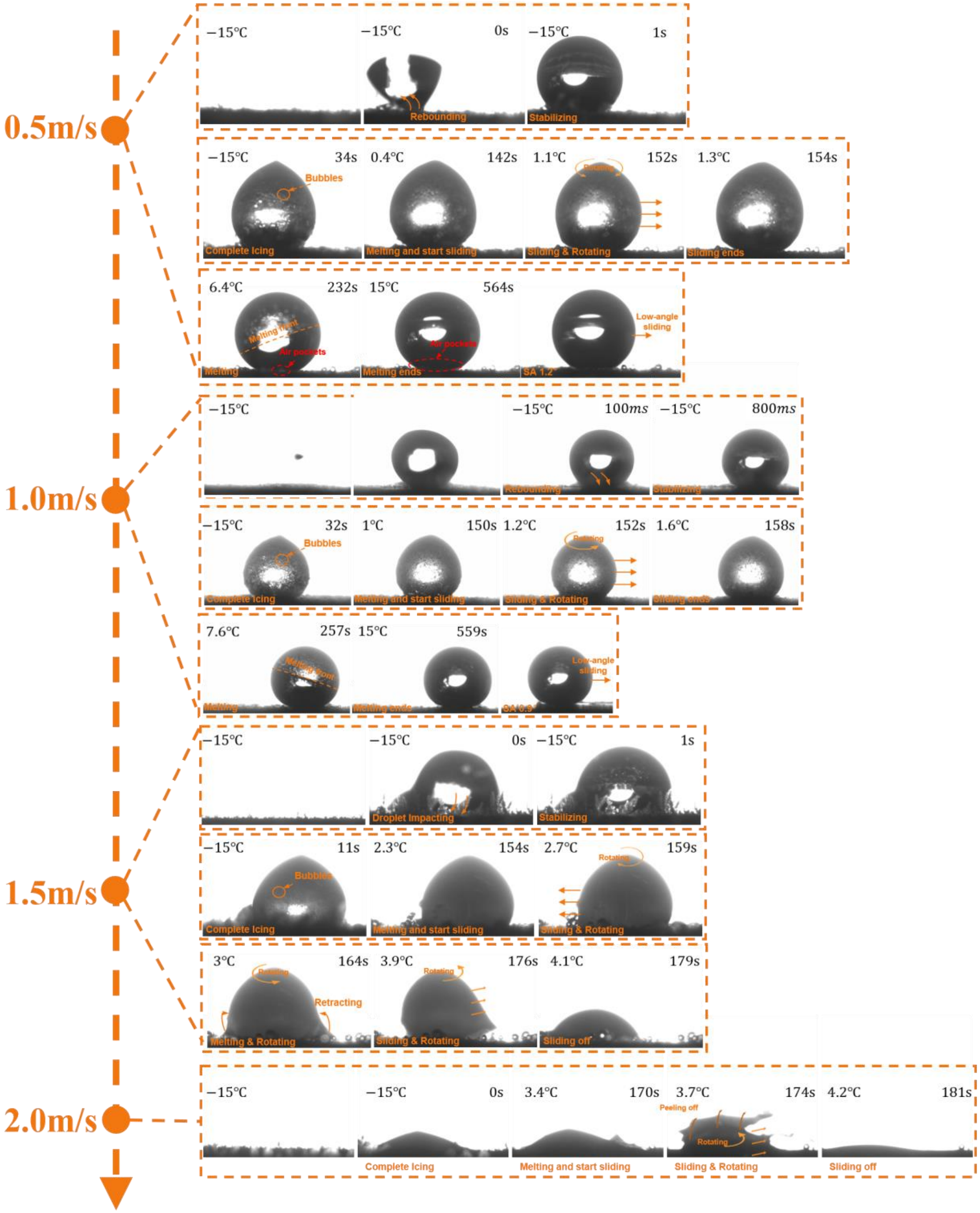

Supplementary Figure 41. The icing & melting phenomena for droplets dynamic impacting with different impacting speeds. The rotating directions, melting fronts and bubbles are marked with orange lines. Different stages of icing and melting processes are also indicated.

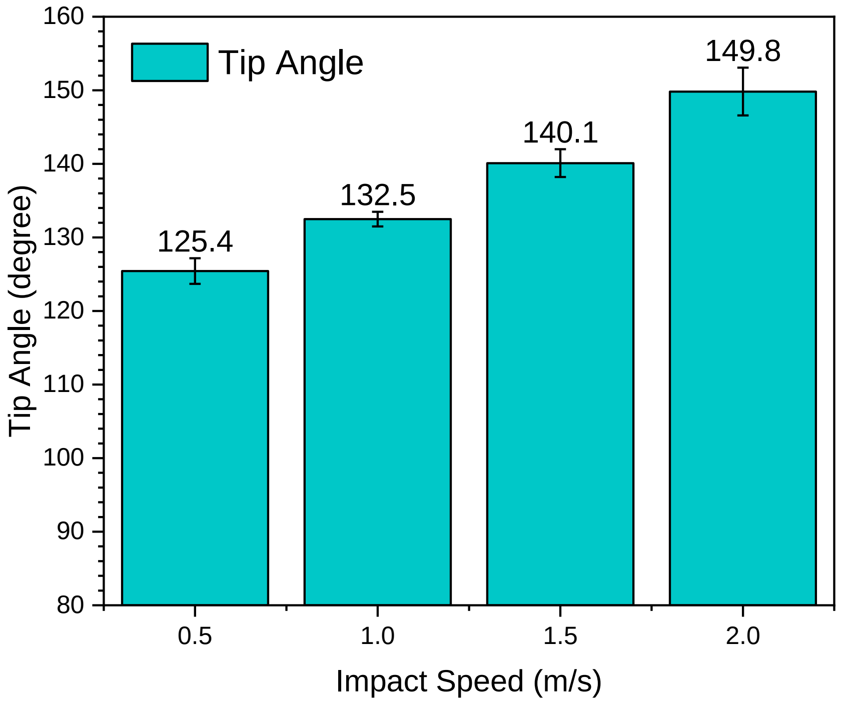

Supplementary Figure 42. The ice tip angles for ice droplets formed by dynamic impacting with different impacting speeds. Data are mean ± s.d. from at least three independent measurements.

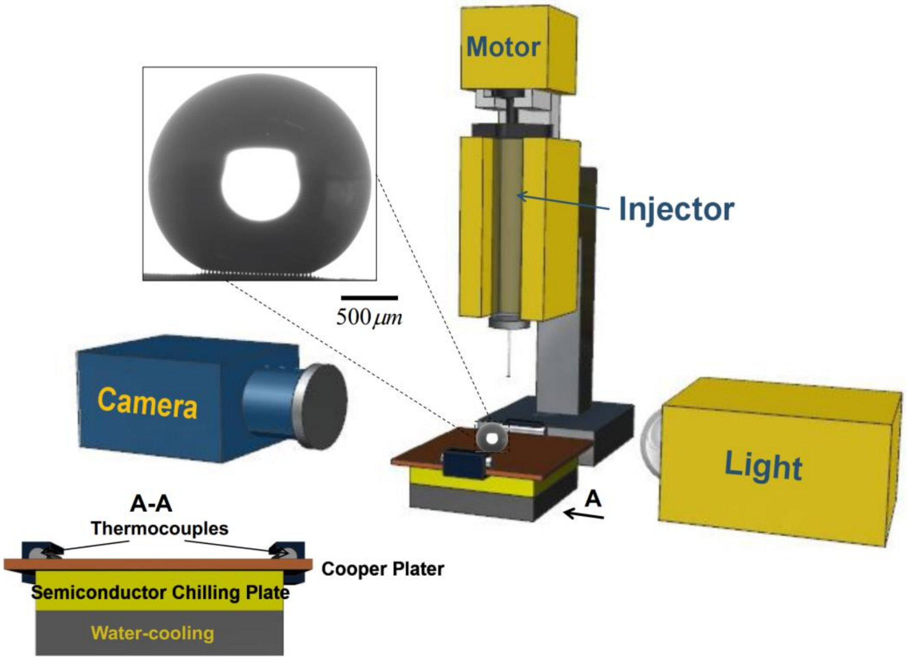

Supplementary Figure 43. Schematic diagram of the experimental setup.

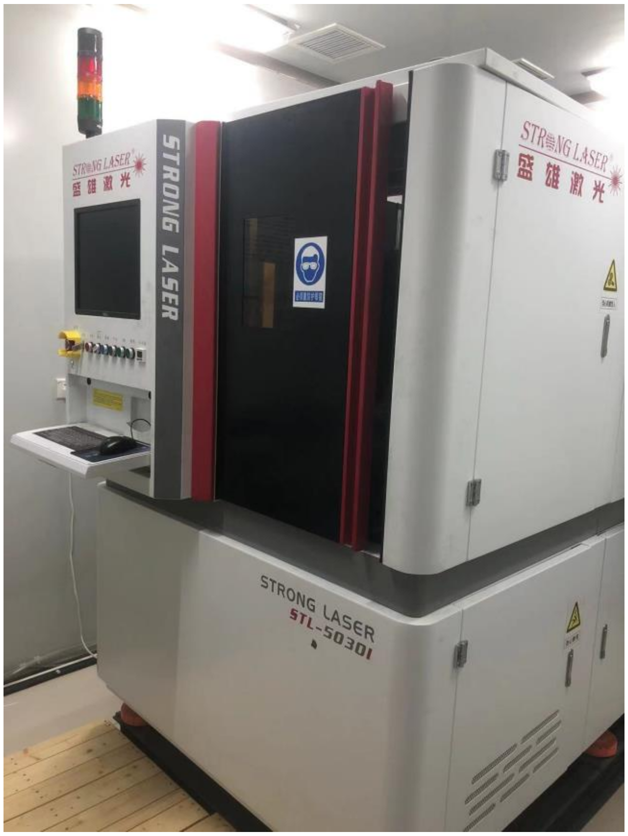

Supplementary Figure 44. Photo of laser manufacturing setup

	Cavity 1
I_C_MAX	30.50 A
I_C_100	25.50 A
I_C_THRES	2.00 A

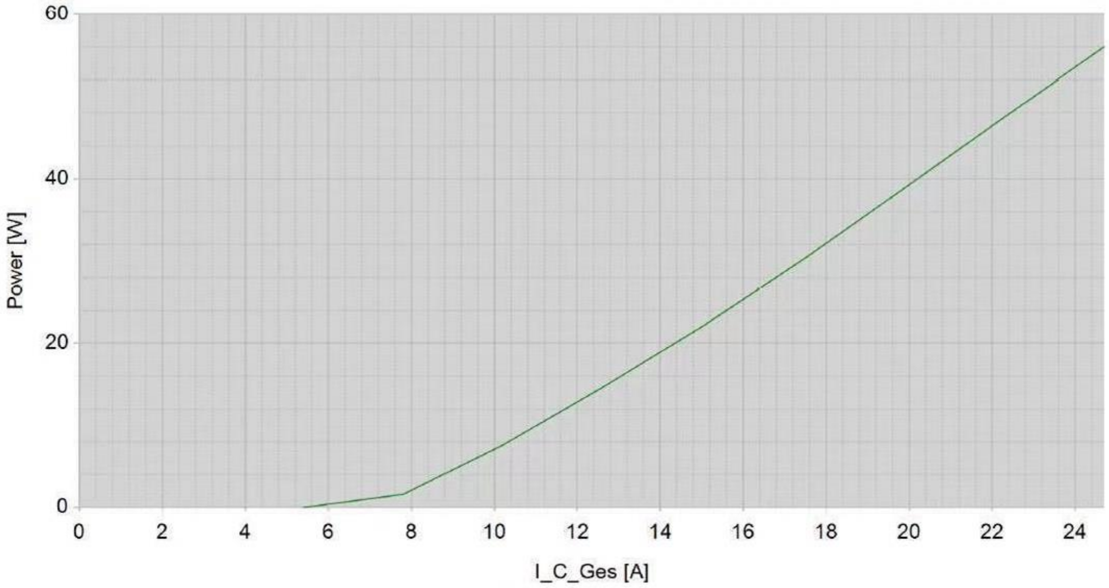

Supplementary Figure 45. Dependence of output energy on the input energy.

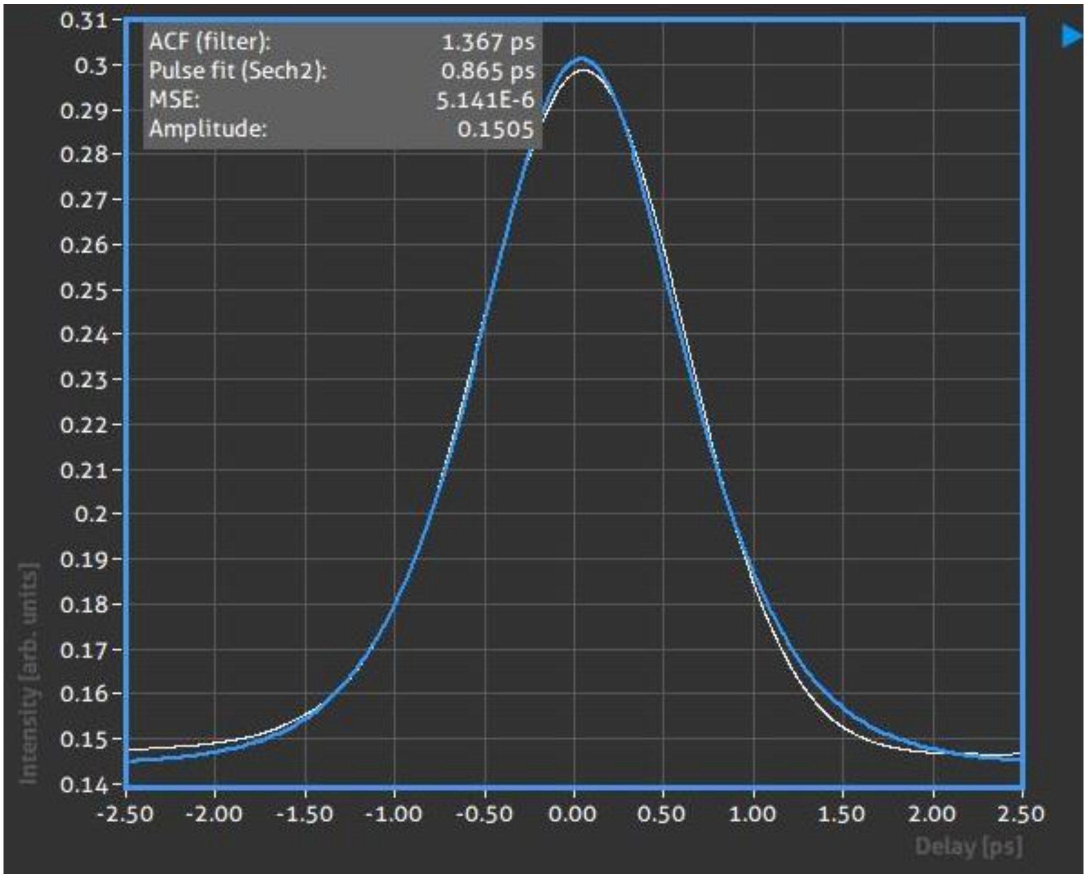

Supplementary Figure 46. Dependence of output intensity on the delay.

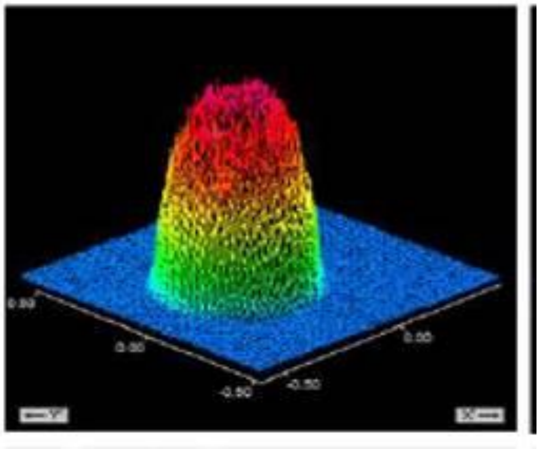

Supplementary Figure 47. Distribution of output energy of laser.

ISO-Norm-Messung

Caustic Measurement

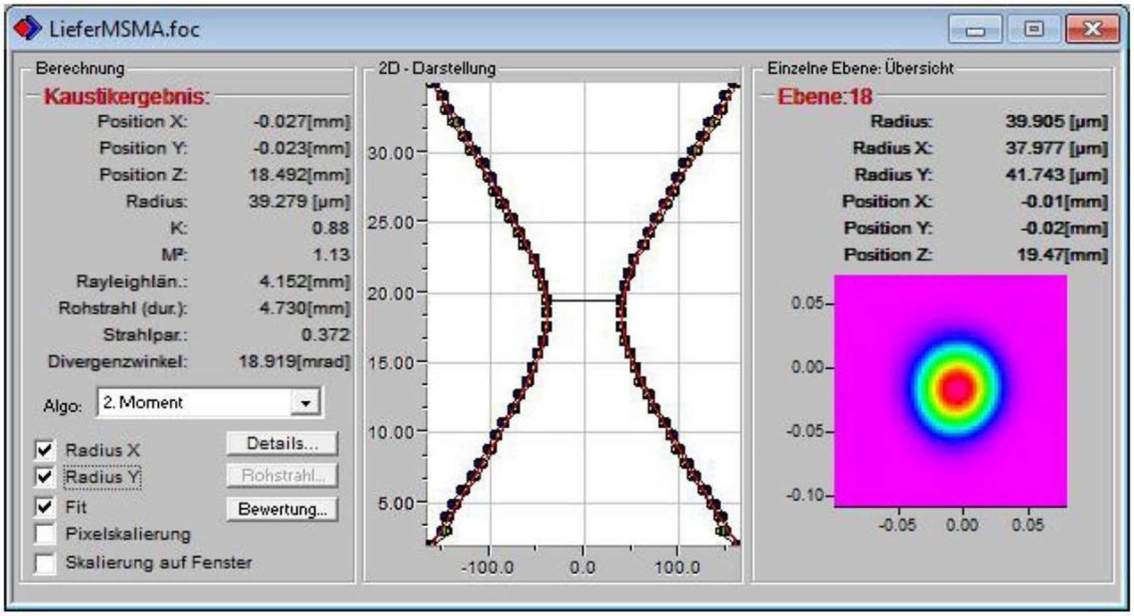

Supplementary Figure 48. Normalized two-dimensional measurement diagram of output energy.

Supplementary Tables:

Supplementary Table 1. Specific laser processing parameters for fabricating different materials.

Types	Processing	Laser Fluence (J/cm^2)	Scanning Speed (mm/s)	Repetition Rate	Pulse Duration	Scanning Route	Scanning Pitch (μm)
MCNP	One Irradiation	1.43	500	200kHz	800fs	Crossed lines	35
SMC	First Irradiation	1.43	500	200kHz	800fs	Crossed lines	35
	Second Irradiation	1.43	2000	200kHz	800fs	Crossed lines	35
MBNP	One Irradiation	3.23	50	200kHz	800fs	Crossed lines	10

Supplementary Table 2. Laser processing parameters for different microcones heights and pitches.

Types	Processing	Laser Fluence (J/cm^2)	Scanning Speed (mm/s)	Repetition rate	Pulse Duration	Scanning Route	Scanning Pitch (μm)	Repeated number
H35 P35	One Irradiation	1.43	500	200kHz	800fs	Crossed lines	35	8
H45 P35	One Irradiation	1.43	500	200kHz	800fs	Crossed lines	35	18
H55 P35	One Irradiation	1.43	500	200kHz	800fs	Crossed lines	35	30
H45 P45	One Irradiation	1.43	500	200kHz	800fs	Crossed lines	45	18
H45 P25	One Irradiation	1.43	500	200kHz	800fs	Crossed lines	25	18

Supplementary Table 3. Laser parameters.

Description	Nom.-/Limit val.	Value
Laser type	-	200kHz
Wavelength	-	1030nm
Average laser power	-	40W
Threshold value	-	$0.00115 W/cm^2$
Beam diameter	5+/-0,5	4.7mm
Beam quality M^2	<1,3	passed
Beam quality M^2x	-	1.12
Beam quality M^2y	-	1.14
Polarisation plane	-	vertical
Total working time	-	7557h

Supplementary Table 4. The critical Laplace pressures of different surfaces

Surface Types	Different Critical Laplace Pressure (Pa)		
	P_{CCA}	P_{CTD}	P_{CCB}
MCNP	897	1205	897
MNSF	1035	1383	1035
MNGF	912	942	912
MNS	465	1246	465
MNR	534	678	534
MNG	954	1503	954

Supplementary Table 5. The detailed parameters for droplet dynamic impacting with different impacting speeds

Sets	Droplet Releasing Heights (mm)	Impacting Velocity(m/s)	Weber Number
1	12.76	0.5	7.25
2	51.02	1.0	29.02
3	114.68	1.5	65.29
4	203.87	2.0	116.06

Supplementary Method 1. Laser processing parameters and topologies of the samples

The micro-nanostructure topologies of four types of hydrophobic surfaces are presented in Fig. 1. Thereinto, the MCNP, SMC and MBNP surfaces were fabricated by ultrafast laser, and the IMN surface was processed by chemical etching. The laser processing parameters of the former three surfaces are shown in Supplementary Table 1. The topologies of the four surfaces can also be seen in Supplementary Figure 1. By scanning the 3D topologies of the MCNP and SMC surfaces and observing their cross sections, we find the microcones height of SMC surfaces is almost equal to that of MCNP surfaces due to few fast-scanning times, as shown in Supplementary Figure 2.

Supplementary Method 2. Laser processing parameters, topologies and wettability of the MNCP surfaces with different microcones heights and pitches.

The laser processing parameters for different microcones heights and pitches of the MCNP surfaces are shown in Supplementary Table 2. By adjusting the repeated scanning number, the MCNP surfaces with different microcones heights can be acquired. The 3D topologies and scanning section of the MCNP surfaces with microcones heights of $35\mu m$, $45\mu m$ and $55\mu m$ are presented in Supplementary Figure 20. Similarly, the MCNP surfaces with tunable microcones pitches can fabricated by changing the scanning pitches. Supplementary Figure 21 shows the topologies of MCNP surfaces with the microcones pitches of $25\mu m$, $35\mu m$ and $45\mu m$.

After being chemically modified by fluoroalkyl silane, we tested the contact angles and sliding angles of the MCNP surfaces with different microcones heights and pitches at room temperature (15℃ and a humidity 20%), as shown in Supplementary Figure 22. It can be clearly

observed that the superhydrophobicity will weaken with the decrease of microcones heights or the increase of microcones pitches.

Supplementary Method 3. Experimental setup

The schematic of experimental setup is presented in Supplementary Figure 43. The side view of Peltier cooling plate is emphasized in the A-A view.

Supplementary Figure 44 shows the picture of laser fabrication equipment. We utilized a Trumpf TruMicro 5000 ultrafast laser system with 800 fs pulses at a central wavelength of 1030 nm and a repetition rate of 200kHz to fabricate the micro-nanostructures. The output of this equipment is the unpolarized light, and the operation temperature is at 22℃. Laser performances and parameters are shown in Supplementary Figures 45-48 and Supplementary Table 3.

Supplementary Discussion 1. Comparison and records of droplets on four types of surfaces during icing & melting cycle

Supplementary Figure 3 presents the comparison of droplet state changes on four types of surfaces during icing and melting processes. It can be clearly observed that the droplets on the MCNP surfaces not only have a slower deterioration rate of contact diameters and angles during the icing process, but also have a prominent recovery of contact diameters and angles during the melting process. The contact angle after droplets melting can even reach up to 156.69°. On the contrary, although the MBNP, IMN and SMC surfaces have the similar deterioration of superhydrophobicity, their contact diameters and angles are almost unchanged and still stay the deteriorated state during the melting process. The contact angles after complete recovery on the

three surfaces are still less than 140°. The changes of the contact diameters and angles of the other three surfaces during the icing & melting cycle are also shown in Supplementary Figures 4-6.

Supplementary Discussion 2. The interfacial thermal resistance of different wetting states

We adopted the quasi-steady thermal current model to analyze the interfacial thermal resistances of different wetting states.

For the CB-CB wetting state, the thermal resistance consists of air layer, hydrophobic layer, nanoparticles and microcones[1]. Because the thermal resistance of air layer is much larger than those of the other three, the thermal resistance of the CB-CB state can be equivalent to the later three thermal resistances in series. Therefore, the thermal resistance of the CB-CB state can be expressed by:

$$R_{\mathrm{CB-CB}} = \frac{1}{\pi r_{\mathrm{d}}^2 \sin^2\theta} \cdot \left(\frac{\delta_{\mathrm{c}}}{k_{\mathrm{c}} f_{\mathrm{m}} f_{\mathrm{n}}} + \frac{\delta_{\mathrm{m}}}{k_{\mathrm{m}} f_{\mathrm{m}}} + \frac{\delta_{\mathrm{n}}}{k_{\mathrm{n}} f_{\mathrm{m}} f_{\mathrm{n}}}\right) \quad (1)$$

where k_{m}, k_{n}, k_{c} and δ_{m}, δ_{n}, δ_{c}, are the thermal conductivity and thickness related to the aluminum alloy microcones, nanoparticles and hydrophobic coating; f_{m} and f_{b} are the area fraction of the top part and the bottom part of microcones; f_{w} is the wetting area fraction of micro-nanostructure; r_{d} is the radius of droplet; and θ is the apparent droplet contact angle. In our work, θ is $150°$, $k_{\mathrm{m}} = k_{\mathrm{n}} = 154.11\mathrm{W}\cdot\mathrm{m}^{-1}\cdot\mathrm{K}^{-1}$, $k_{\mathrm{c}} = 0.1\mathrm{W}\cdot\mathrm{m}^{-1}\cdot\mathrm{K}^{-1}$, $\delta_{\mathrm{c}} = 10\mathrm{nm}$, $\delta_{\mathrm{m}} = 45\mu\mathrm{m}$, $\delta_{\mathrm{n}} = 400\mathrm{nm}$. f_{m} and f_{b} are related to the micro-nanostructure, and they can be calculated by:

$$\begin{cases} f_{\mathrm{m}} = \frac{\pi d_{\mathrm{t}}^2}{4\Lambda^2} \\ f_{\mathrm{n}} = \frac{\pi d_{\mathrm{p}}^2}{2\sqrt{3}\mathrm{b}^2} \end{cases} \quad (2)$$

where d_{t} and d_{p} present the diameter of microcones top part and nanoparticles; Λ is the microcones pitch; b is the nanoparticles pitch. Here, $d_{\mathrm{t}} = 4\mu\mathrm{m}$, $d_{\mathrm{p}} = 400\mathrm{nm}$, $\Lambda = 35\mu\mathrm{m}$ and $b = 500\mathrm{nm}$. The schematic of micro-nanostructure is shown in Supplementary Figure 12.

For the W-CB wetting state, the droplet penetrates into the nanoparticles, while the microcones are not be pierced completely. This condition is always applied to the surfaces with dense microcones attached with loose nanoparticles. Therefore, the equivalent thermal resistance for this condition can be regarded as the series connection of the hydrophobic coating and microcones thermal resistances. The overall thermal resistance can be expressed by:

$$R_{\mathrm{W-CB}} = \frac{1}{\pi r^2 \sin^2\theta} \cdot \left(\frac{\delta_{\mathrm{c}}}{k_{\mathrm{c}} f_{\mathrm{m}}} + \frac{\delta_{\mathrm{m}}}{k_{\mathrm{m}} f_{\mathrm{m}}}\right) \quad (3)$$

For the CB-W wetting state, the condition is contrary to that of the W-CB state. The droplets cannot permeate in the nanoparticles, but the microcones are permeated completely. Generally, more dense nanoparticles and looser microcones can result in this wetting mode more easily. The interfacial thermal resistance of this mode is more complicated than those of the former two modes, and consists of the thermal resistances from the top, side and bottom. Hence, the thermal resistance of the CB-W state is given by,

$$R_{\mathrm{CB-W}} = \frac{1}{\pi r^2 \sin^2\theta} \cdot \left(\frac{k_{\mathrm{c}} k_{\mathrm{m}} f_{\mathrm{m}} f_{\mathrm{n}}}{\delta_{\mathrm{c}} k_{\mathrm{m}} + \delta_{\mathrm{m}} k_{\mathrm{c}} f_{\mathrm{n}} + \delta_{\mathrm{n}} k_{\mathrm{c}}} + \frac{k_{\mathrm{c}} k_{\mathrm{m}} k_{\mathrm{n}} (1-f_{\mathrm{m}})(1-f_{\mathrm{b}}) f_{\mathrm{w}} f_{\mathrm{n}}}{\delta_{\mathrm{c}} k_{\mathrm{m}} k_{\mathrm{n}} + \delta_{\mathrm{n}} k_{\mathrm{c}} k_{\mathrm{m}} + \delta_{\mathrm{m}} k_{\mathrm{c}} k_{\mathrm{n}} f_{\mathrm{n}}/2} + \frac{k_{\mathrm{c}} (1-f_{\mathrm{m}}) f_{\mathrm{b}} f_{\mathrm{w}} f_{\mathrm{n}}}{\delta_{\mathrm{c}}}\right)^{-1} \quad (4)$$

For the W-W wetting state, both of the microcones and nanoparticles are completely penetrated. Similar to the thermal resistance of the CB-W state, the thermal resistance of the W-W state is given by

$$R_{\mathrm{W-W}} = \frac{1}{\pi r_{\mathrm{d}}^{2}\sin^{2}\theta} \cdot \left(\frac{k_{\mathrm{c}}k_{\mathrm{m}}f_{\mathrm{m}}}{\delta_{\mathrm{c}}k_{\mathrm{m}}+\delta_{\mathrm{m}}k_{\mathrm{c}}} + \frac{k_{\mathrm{c}}k_{\mathrm{m}}(1-f_{\mathrm{m}})(1-f_{\mathrm{b}})f_{\mathrm{w}}}{\delta_{\mathrm{c}}k_{\mathrm{m}}+\frac{\delta_{\mathrm{m}}k_{\mathrm{c}}}{2}} + \frac{k_{\mathrm{c}}(1-f_{\mathrm{m}})f_{\mathrm{b}}f_{\mathrm{w}}}{\delta_{\mathrm{c}}}\right)^{-1} \quad (5)$$

The comparison figure and interfacial thermal resistance of the four wetting states are shown in Figs. 6a and b.

Supplementary Discussion 3. Calculation of total bubbles volume in the ice droplet

It is understandable that more and denser bubbles in a specific ice droplet tend to lead to a higher probability to achieve the downward movement of bubbles. However, due to the limited characterization devices, the datum of 3D bubbles in the ice droplets cannot yet be obtained in experiments. Chu et al. reported the theoretical calculation methods of total bubbles volume in the ice droplet[2]. The total bubbles volume can be expressed as

$$V_{\mathrm{tb}} = \pi\left(1+\frac{1}{\nu\alpha}\right)^{-1}\int_{0}^{t}\left[\left(r_{\mathrm{d}}^{2}\sin^{2}\theta - 2S_{\mathrm{t}}D\cdot t - 2r_{\mathrm{d}}(2S_{\mathrm{t}}D\cdot t)^{1/2}\cos\theta\right)\cdot\left(\frac{S_{\mathrm{t}}D}{2t}\right)^{1/2}\right]dt \quad (6)$$

While ν is the density ratio of ice and water, here, we take 0.92; α is the air solubility in supercooled water, which takes the values of 3.6%, 4.4%, 5.04% when the temperature of the supercooled water is -5℃, -10℃, -15℃, respectively; S_{t} is the Stefan number, $S_{\mathrm{t}} = C_{\mathrm{p}}\Delta T_{\mathrm{c}}/L_{\mathrm{m}}$; D is the thermal diffusivity, $D = \lambda_{\mathrm{ice}}/(\rho_{\mathrm{ice}}C_{\mathrm{p}})$; t denotes the time, which is recorded from the beginning of icing;

C_p is the heat capacity at constant pressure; ΔT_c represents the supercooled temperature; L_m is the latent heat when water freezes into ice; λ_{ice} is the thermal conductivity of ice; ρ_{ice} is the ice density.

From the above equation, it can be found that the total bubbles volume is closely related to the supercooled temperature ΔT_c, the icing time t and the contact angle of the ice droplet. Supplementary Figure 13 shows the total bubbles volume under different supercooled temperatures. The total bubbles volume of ice droplet increases significantly with the increase of ΔT_c and t. This indicates that more delayed icing time and more icing time can trap more bubbles in the ice droplet. Supplementary Figure 14 shows the total bubbles volumes in the ice droplets on four hydrophobic surfaces. It can be clearly observed that there are bubbles with a total volume of 0.21μL frozen in an ice droplet on the MCNP surface, while the total air pockets volume in the MCNP surface is only 0.0156μL. Therefore, there are abundant bubbles in the ice droplet to support the recovery of air pockets.

Supplementary Discussion 4. Design principles of superhydrophobic surfaces

Superhydrophobic surfaces need to meet two conditions[3]: (i) high contact angles of greater than 150°; (ii) low sliding angle of less than 10°. Thereinto, the contact angle can be calculated by：

$$\cos\theta = f_{\mathrm{top}} f_2 (\cos\theta_0 + 1) - 1 \tag{7}$$

The adhesion strength of droplets on the superhydrophobic surfaces can be expressed as follows[4]:

$$F_{\mathrm{as}} = 2r_{\mathrm{d}}\gamma_{\mathrm{lv}}\sin\theta(1+\cos\theta_0)\sqrt{f_{\mathrm{top}}f_2} \tag{8}$$

Where F_{as} denotes the adhesion strength of droplets on the substrate; γ_{lv} is the liquid−vapor interfacial energy, $\gamma_{\mathrm{lv}} = 73.49\mathrm{mN/m}$.

Supplementary Figure 15a shows the contact angles and adhesion strength under different liquid-solid contact fractions. To meet the two conditions of superhydrophobic surfaces, the solid fraction of the micro-nanostructure f should be less than 0.05. The critical design zones for contact angles and sliding angles are marked in Supplementary Figures 15b and c, respectively.

Supplementary Discussion 5. Theoretical analysis for the wetting and dewetting transitions

The C2W transition can be divided into three stages: (i) the meniscus expands with the increase of the sag angle (α_1); (ii) the three-phase-contact-line (TPCL) moves downwards, when the sag angle is θ_{adv}; (iii) the TPCL touches the bottom of the substrate so that the substrate is wetted, and the vapor in the air pockets is dissolved in the droplet completely[5, 6]. In the first stage, the droplet is in CB-CB state, where the system energy can be calculated by

$$G_{\mathrm{CB-CB}} = G_0 + N\pi {r_{\mathrm{eff}}^{\mathrm{g}}}^2\gamma_{\mathrm{lv}}\left(\frac{2}{1+\sin\alpha_1} - 1\right) + P_0 \cdot \left(\frac{V_0 T}{288.15}\right)\ln\left(\frac{H_{\mathrm{p}}}{H_{\mathrm{p}} - h_{\mathrm{m}}^{\mathrm{eff}}}\right) \tag{9}$$

The nondimensionalized total Gibbs free energy can be expressed as

$$G_{\mathrm{CB-CB}}^{*} = \frac{(G_{\mathrm{CB-CB}} - G_0)(1-f_1)}{N\pi {r_{\mathrm{eff}}^{\mathrm{g}}}^2\gamma_{\mathrm{lv}}} = (1-f_1)\left(\frac{2}{1+\sin\alpha_1} - 1\right) + (1-f_1)P_0 \cdot \left(\frac{V_0 T}{288.15 \cdot N\pi {r_{\mathrm{eff}}^{\mathrm{g}}}^2\gamma_{\mathrm{lv}}}\right)\ln\left(\frac{H_{\mathrm{p}}}{H_{\mathrm{p}} - h_{\mathrm{m}}^{\mathrm{eff}}}\right)\ (\pi/2 \le \alpha_1 \le \theta_{\mathrm{adv}}) \tag{10}$$

While G_0 denotes the original system energy; $G^*_{\mathrm{CB-CB}} = (G_{\mathrm{CB-CB}} - G_0)(1-f_1)/N\pi {r^{\mathrm{g}}_{\mathrm{eff}}}^2 \gamma_{\mathrm{lv}}$; $r^{\mathrm{g}}_{\mathrm{eff}}$, $r^{\mathrm{c}}_{\mathrm{eff}}$ denote the equivalent geometric radius and the equivalent capillary radius for hierarchically structured surfaces with pillars, respectively, $r^{\mathrm{g}}_{\mathrm{eff}} = (\sqrt{\pi/2f_1} - 1)r_{\mathrm{p}}$, $r^{\mathrm{c}}_{\mathrm{eff}} = (1-f_1)r_{\mathrm{p}}/f_1$; r_{p} is the pillar radius; N denotes the total number of pillars; α_1 denotes the sag angle of liquid-vapor interface; P_0 denotes the ambient air pressure, $P_0 = 101.325\mathrm{kPa}$; V_0 is the air pocket volume in one cell; T is the temperature of the air pocket; H_{p} is the pillar height; $h^{\mathrm{eff}}_{\mathrm{m}}$ is the equivalent value of h_{m}; h_{m} is the meniscus height.

In the second stage, the droplet is in a transition stage between the CB-CB stage and the CB-W stage. The sag angle of the meniscus stays constant, which is equal to θ_{adv}. The system energy of this stage can be expressed as

$$G_{\mathrm{CB-CB\to CB-W}} = G_0 + N\pi {r^{\mathrm{g}}_{\mathrm{eff}}}^2 \gamma_{\mathrm{lv}} \left(\frac{2}{1+\sin\alpha_1} - 1\right) - N\pi {r^{\mathrm{g}}_{\mathrm{eff}}}^2 \gamma_{\mathrm{lv}} \frac{2f_1 x(f_2\cos\theta_0 + f_2 - 1)}{(1-f_1)r_{\mathrm{p}}} + P_0 \cdot \left(\frac{V_0 T}{288.15}\right) \ln\left(\frac{H_{\mathrm{p}}}{H_{\mathrm{p}} - (h^{\mathrm{eff}}_{\mathrm{m}})_{\mathrm{max}} - x}\right) \tag{11}$$

The nondimensionalized total free energy can expressed as

$$G^*_{\mathrm{CB-CB\to CB-W}} = (1-f_1)\left(\frac{2}{1+\sin\alpha_1} - 1\right) - \frac{2f_1 x(f_2\cos\theta_0 + f_2 - 1)}{r_{\mathrm{p}}} + (1-f_1)P_0 \cdot \left(\frac{V_0 T}{288.15 \cdot N\pi {r^{\mathrm{g}}_{\mathrm{eff}}}^2 \gamma_{\mathrm{lv}}}\right) \ln\left(\frac{H_{\mathrm{p}}}{H_{\mathrm{p}} - (h^{\mathrm{eff}}_{\mathrm{m}})_{\mathrm{max}} - x}\right) \ (0 \le x < H_p - h_m) \tag{12}$$

While x is the downward displacement of three-phase contact line; $(h^{\mathrm{eff}}_{\mathrm{m}})_{\mathrm{max}}$ is the maximum value of $h^{\mathrm{eff}}_{\mathrm{m}}$.

In the third stage, the bottom of the substrate is completely wetted, and the vapor in the air pocket is totally dissolved in the droplet. The system energy of this state can be calculated by

$$G_{\mathrm{CB-W}} = G_0 - N\pi {r_{\mathrm{eff}}^{\mathrm{g}}}^2 \gamma_{\mathrm{lv}} - N\pi {r_{\mathrm{eff}}^{\mathrm{g}}}^2 \gamma_{\mathrm{lv}} (1 + \frac{2f_1 H_{\mathrm{p}}}{(1-f_1) r_{\mathrm{p}}})(f_2 \cos\theta_0 + f_2 - 1) + P_0 \cdot (\frac{V_0 T}{288.15}) \quad (13)$$

The nondimensionalized total free energy of this stage can expressed as

$$G_{\mathrm{CB-W}}^{*} = -(1 - f_1) - (1 - f_1)(1 + \frac{2f_1 H_{\mathrm{p}}}{(1-f_1) r_{\mathrm{p}}})(f_2 \cos\theta_0 + f_2 - 1) - G_{P_0}^{*} \quad (14)$$

Meanwhile, the h_{m}, $h_{\mathrm{m}}^{\mathrm{eff}}$, $(h_{\mathrm{m}}^{\mathrm{eff}})_{\max}$ can be respectively calculated by

$$h_{\mathrm{m}} = \frac{r_1(2 - 3\sin\alpha_1 + \sin^3\alpha_1)}{-3\cos^3\alpha_1} \quad (15)$$

$$h_{\mathrm{m}}^{\mathrm{eff}} = \frac{f_1}{(1-f_1)} (\sqrt{\frac{\pi}{2f_1}} - 1)^3 h_{\mathrm{m}} \quad (16)$$

$$(h_{\mathrm{m}}^{\mathrm{eff}})_{\max} = \frac{f_1}{(1-f_1)} \left(\sqrt{\frac{\pi}{2f_1}} - 1\right)^3 (h_{\mathrm{m}})_{\max} \quad (17)$$

Supplementary Figure 16 depicts the changes of the system free energy with the increase of the penetration depth $h_{\mathrm{p}}/H_{\mathrm{p}}$. The total free energy of the system increases gradually with the increase of the penetration depth. When the meniscus touches the bottom substrate, the bottom of the substrate is wetted. The excess vapor in the air pockets is compressed into the droplet, resulting in the sudden decrease of the total free energy of the system.

In terms of the former analysis, there are three kinds of energy barriers during the wetting process. In the first stage, the meniscus energy barrier needs to be overcome, which can be expressed by

$$G^*_{\text{meniscus}} = (1 - f_1)(\frac{2}{1+\sin\theta_{\text{adv}}} - 1) \quad (18)$$

As the meniscus moves downwards, the capillary energy barrier exits due to the vapor compression in the air pockets. The capillary energy barrier can also be described by

$$G^*_{\text{capillary}} = -\frac{2f_1 x(f_2\cos\theta_0 + f_2 - 1)}{r_{\text{p}}} \quad (19)$$

During the wetting process, the vapor in the air pockets is assumed not dissolved in the droplet. The air pockets energy barrier is relation to the penetration depth. Therefore, it can be expressed by

$$G^*_{\text{gas}} = \begin{cases} (1 - f_1)P_0 \cdot (\frac{V_0 T}{288.15 \cdot N\pi {r^{\text{g}}_{\text{eff}}}^2 \gamma_{\text{lv}}}) \ln(\frac{H_{\text{p}}}{H_{\text{p}} - h^{\text{eff}}_{\text{m}}}) \ (\pi/2 \le \alpha_1 \le \theta_{\text{adv}}) \\ (1 - f_1)P_0 \cdot (\frac{V_0 T}{288.15 \cdot N\pi {r^{\text{g}}_{\text{eff}}}^2 \gamma_{\text{lv}}}) \ln(\frac{H_{\text{p}}}{H_{\text{p}} - \left(h^{\text{eff}}_{\text{m}}\right)_{\max} - x}) \ (\alpha_1 = \theta_{\text{adv}}, 0 \le x \le H_{\text{p}} - h^{\text{eff}}_{\text{m}}) \end{cases}$$

(20)

According to the energy barriers during the wetting process, there are two conditions for the dewetting transitions. Firstly, the system energy of the wetted state (CB-W state in this work) should be higher than that of the dewetted state (CB-CB state) so that the CB-CB state is a more stable state. Secondly, the energy barrier during wetting process should be higher than the energy release of the system after wetting. If a surface meets the two conditions in the meantime, the superhydrophobicity of the surface will be monostable, and the reversible transition would occur even if the surface was wetted under some specific external conditions

For the first condition, it can be summarized by

$$\Delta G^*_1 = G^*_{\text{CB-W}} - G^*_{\text{CB-CB}} = -(1 - f_1) - (1 - f_1)(1 + \frac{2f_1 H_{\text{p}}}{(1-f_1)r_{\text{p}}})(f_2\cos\theta_0 + f_2 - 1) - G^*_{P_0} \ge 0 \quad (21)$$

While $\Delta G_1^* = \Delta G_1(1-f_1)/N\pi {r_{\mathrm{eff}}^{\mathrm{g}}}^2\gamma_{\mathrm{lv}}$

For the second condition, the energy release of the system can be attributed to the decrease of surface energy of the system. The total surface energy in the CB-CB state is

$$G_{\mathrm{CB-CB}}^{\mathrm{s}} = (1-f_1)2\pi(\frac{r_{\mathrm{eff}}^{\mathrm{g}}}{\cos\theta_{\mathrm{adv}}})^2\gamma_{\mathrm{lv}}[1-\cos(\theta_{\mathrm{adv}}-\frac{\pi}{2})] + (1-f_1)\gamma_{\mathrm{sv}}f_2\pi {r_{\mathrm{eff}}^{\mathrm{g}}}^2 \quad (22)$$

Considering that the trapped air dissolves into droplets as the decrease of the substrate temperature, therefore we take $\cos\theta_{\mathrm{adv}}$ instead of $\cos\theta$.

When the droplet penetrates into the micro-nanostructure, the total surface energy in the CB-W state is

$$G_{\mathrm{CB-W}}^{\mathrm{s}} = (1-f_1)f_2\gamma_{\mathrm{sl}}\pi {r_{\mathrm{eff}}^{\mathrm{g}}}^2 + (1-f_1)(1-f_2)\gamma_{\mathrm{lv}}\pi {r_{\mathrm{eff}}^{\mathrm{g}}}^2 \quad (23)$$

The energy release during the wetting process can be expressed as

$$\Delta G^{\mathrm{s}} = G_{\mathrm{CB-CB}}^{\mathrm{s}} - G_{\mathrm{CB-W}}^{\mathrm{s}} = (1-f_1)\pi {r_{\mathrm{eff}}^{\mathrm{g}}}^2\gamma_{\mathrm{lv}}\frac{2}{1+\sin\theta_{\mathrm{adv}}} + (1-f_1)\pi {r_{\mathrm{eff}}^{\mathrm{g}}}^2\gamma_{\mathrm{lv}}(f_2\cos\theta_0 + f_2 - 1) \quad (24)$$

The energy barriers for the wetting process consist of the capillary energy barrier and the air pockets energy barrier. The capillary energy barrier can be calculated by

$$G_{\mathrm{CB-CB}}^{\mathrm{capillary}} = -(1-f_1)\pi {r_{\mathrm{eff}}^{\mathrm{g}}}^2\gamma_{\mathrm{lv}}\frac{r_{\mathrm{eff}}^{\mathrm{g}}}{r_{\mathrm{eff}}^{\mathrm{c}}}\frac{2(\sin\theta_{\mathrm{adv}}-1)}{\cos\theta_{\mathrm{adv}}}(f_2\cos\theta_0 + f_2 - 1) \quad (25)$$

During the melting process, bubbles in the ice droplets would impact the bottom micro-nanovalleys to provide the vapor source for the recovery of the air pockets, therefore, the air pockets energy barrier exists to prompt the W2C transition. The air pockets energy barrier can be expressed as

$$G_{\mathrm{CB-CB}}^{\mathrm{v}} = P_0 \pi {r_{\mathrm{eff}}^{\mathrm{g}}}^3 [\frac{\sin\theta - 1}{\cos\theta} + \frac{(1-\sin\theta)^2(2+\sin\theta)}{3\cos^3\theta}] \tag{26}$$

Considering the contributions of bubbles for the recovery of the air pockets cannot be complete for the thorough recovery of the air pockets, we introduce the recovery factor ε_{r} to stand for the bubble contribution for the recovery of the bottom air pockets. The value of ε_{r} ranges from 0 to 1. When ε_{r}=0, no bubble impacts downwards to contribute for the air pockets recovery. When $\varepsilon_{\mathrm{r}} = 1$, the bubbles in the ice droplets can contribute to the complete recovery of the air pockets.

Therefore, the second condition equation can be expressed by

$$\Delta G_2 = -G^s + G_{\mathrm{CB-CB}}^{\mathrm{capillary}} + G_{\mathrm{CB-CB}}^{\mathrm{v}} \geq 0 \tag{27}$$

$$\begin{aligned} \Delta G_2 &= -G^{\mathrm{s}} + G_{\mathrm{CB-CB}}^{\mathrm{capillary}} + G_{\mathrm{CB-CB}}^{\mathrm{v}} \\ &= -(1-f_1) N\pi {r_{\mathrm{eff}}^{\mathrm{g}}}^2 \gamma_{\mathrm{lv}} \frac{2}{1+\sin\theta_{\mathrm{adv}}} + (1-f_1) N\pi {r_{\mathrm{eff}}^{\mathrm{g}}}^2 \gamma_{\mathrm{lv}} (f_2\cos\theta_0 + f_2 - 1) \\ &-(1-f_1) N\pi {r_{\mathrm{eff}}^{\mathrm{g}}}^2 \gamma_{\mathrm{lv}} \frac{r_{\mathrm{eff}}^{\mathrm{g}}}{r_{\mathrm{eff}}^{\mathrm{c}}} \frac{2(\sin\theta_{\mathrm{adv}} - 1)}{\cos\theta_{\mathrm{adv}}} (f_2\cos\theta_0 + f_2 - 1) \\ &+P_0 \varepsilon_{\mathrm{r}} N\pi {r_{\mathrm{eff}}^{\mathrm{g}}}^3 [\frac{\sin\theta - 1}{\cos\theta} + \frac{(1-\sin\theta)^2(2+\sin\theta)}{3\cos^3\theta}] \end{aligned} \tag{28}$$

The nondimensionalized total free energy of this stage can expressed as

$$\Delta G_2^* = \frac{\Delta G_2}{N\pi {r_{\mathrm{eff}}^{\mathrm{g}}}^2 \gamma_{\mathrm{lv}}} = -(1-f_1)\frac{2}{1+\sin\theta_{\mathrm{adv}}} + (1-f_1)(f_2\cos\theta_0 + f_2 - 1) - (1-f_1)\frac{r_{\mathrm{eff}}^{\mathrm{g}}}{r_{\mathrm{eff}}^{\mathrm{c}}}\frac{2(\sin\theta_{\mathrm{adv}}-1)}{\cos\theta_{\mathrm{adv}}}(f_2\cos\theta_0 + f_2 - 1) + P_0\varepsilon_{\mathrm{r}}\frac{r_{\mathrm{eff}}^{\mathrm{g}}}{\gamma_{\mathrm{lv}}}[\frac{\sin\theta_{\mathrm{adv}}-1}{\cos\theta_{\mathrm{adv}}} + \frac{(1-\sin\theta_{\mathrm{adv}})^2(2+\sin\theta_{\mathrm{adv}})}{3\cos^3\theta_{\mathrm{adv}}}] \tag{29}$$

Supplementary Figure 17 shows the Gibbs free energy phase diagram under different system energy states. In the Supplementary Figure 17b, when $\Delta G_1^* < 0$, the system is in the classical wetting state, where the Wenzel state is more stable than the CB state, and a huge energy barrier exits between the two wetting states (Supplementary Figure 17a). While for $\Delta G_1^* > 0$, the CB state is more stable than the Wenzel state, but it is still a bistable state. If $\Delta G_2^* > 0$ is met under the condition of $\Delta G_1^* > 0$ (Supplementary Figure 13c), the energy barrier between two wetting states will be enhanced, resulting in the occurrence of the monostable CB state. Droplets tend to transit to the CB state even if the state is destroyed by external conditions temporarily. The zone distributions of different energy states are shown in Fig. 7b.

Supplementary Figure 18 depicts the phase diagram of ΔG_2^* under different ε_{r}. It can be surprising observed that the monostable zone could be extended with the increase of ε_{r}. The micro-nanostructure of the MCNP surface in this work could be equivalent to the regular pillars. The equivalent result is marked in the Supplementary Figure 18. When $\varepsilon_{\mathrm{r}} = 0$ or $\varepsilon_{\mathrm{r}} = 0.01$, the MCNP surface is not in the monostable zone. As ε_{r} increases continuously until $\varepsilon_{\mathrm{r}} = 0.03$, our surface is transited to the monostable CB state zone under the effects of bubbles impact. To achieve the monostable state, the ice droplet on the MCNP surface only needs to provide the bubbles volume, which accounts for only 3% of the total air pockets volume. With the continuous increase of ε_{r}, the monostable zone extends significantly. When $\varepsilon_{\mathrm{r}} = 0.09$, the zone of $\Delta G_2^* > 0$ almost takes up most of the area.

Supplementary Figure 19 shows the phase diagram of the occurrence of the dewetting transitions. As shown in figure, the reversible zone expands significantly with the increase of ε_{r}.

The original bistable state could transit to the monostable state when the bubbles impact reaches a specific extent.

Supplementary Discussion 6. Optimal design zone for the superhydrophobic surfaces fabricated by ultrafast laser

Supplementary Figures 23 and 24 depict the effects of different microcones heights and pitches on the dewetting transitions during an icing & melting cycle. As shown in Supplementary Figure 23, the recovery of the Cassie-Baxter state shows a trend of increasing and then decreasing with the increase of microcones heights. When the microcones height increases from $5\mu m$ to $35\mu m$, the contact angles, CDRR and CARR gradually increase, and the sliding angles decrease by degrees due to the superhydrophobicity improvement of the surfaces. As the microcones height continues increasing, the superhydrophobicity of surfaces will not remarkedly improve but the surface resistances will increase to some extent, which blocks the successive retraction of droplets. Therefore, the recovery extents of the Cassie-Baxter state start to decrease. The optimal scopes of microcones heights range from $25\mu m$ to $55\mu m$. Similar to the effects of microcones heights on the dewetting transitions, the recovery of the Cassie-Baxter state also shows the trend of increasing and then decreasing as the microcones pitches increase (Supplementary Figure 24). As the microcones pitches increase from $25\mu m$ to $35\mu m$, the recovery extents gradually increase due to the small surface resistances. However, as the microcones pitches continue to increase, the surface superhydrophobicity starts to deteriorate, leading to the decrease of the recovery extents of the Cassie-Baxter state. When the microcones pitch is greater than $55\mu m$, the melted droplets

even loss the superhydrophobicity. The optimal scopes of microcones pitches range from 25μm to 45μm.

Supplementary Figure 25 depicts the effects of microcones heights and pitches on the bubble impact speed, icing delay time and the bubble movement. As shown in the Supplementary Figures 25a and b, the bubble impact speed presents an increasing and then decreasing trend with the increase of microcones heights and pitches, which are corresponded to the trends in Supplementary Figures 23 and 24. Supplementary Figures 25c and d indicate that the higher microcones heights and the narrower micrcocones pitches lead to more icing delay time. Meanwhile, with the icing delay time increases, the probability of bubbles moving downwards increases.

Supplementary Discussion 7. Icing & melting tests on the surfaces with different Cassie-Baxter stability

Pan et al. reported the chemical oxidation method to manufacture the Cu-substrate superhydrophobic surface with the ultrahigh Cassie-Baxter stability[7,8]. To examine the relationship between dewetting transition ability during icing & melting cycle and Cassie-Baxter stability, we adopted the same chemical oxidation method to fabricate five different micro-nanostructured surfaces: MNSF (Microcone arrays covered with dense nanosheets and dispersedly distributed microflowers), MNGF (Microcones covered with dense nanograsses and dispersedly distributed microflowers), MNS (Microcone arrays covered with dense nanosheets), MNR (Microcone arrays covered with dense nanorods) and MNG (Microcone arrays covered with dense nanograsses). Their SEM images are shown in Supplementary Figure 26.

The Cassie-Baxter stability of different micro-nanostructured surfaces is examined by evaporation experiments (Supplementary Figure 27). PCCB denotes the critical Laplace pressure, which is the critical moment for the loss of Cassie-Baxter stability. It is determined by two critical Laplace pressures: (i) the critical Laplace pressure of contact angle (P_{CCA}), and (ii) the Laplace pressure of the TPCL diameter (P_{CTD}). The value of P_{CCB} can be calculated by the following criteria:

$$\begin{cases} if\ P_{\mathrm{CCA}} \leq P_{\mathrm{CTD}},\ P_{\mathrm{CCB}} = P_{\mathrm{CCA}} \\ if\ P_{\mathrm{CCA}} > P_{\mathrm{CTD}},\ P_{\mathrm{CCB}} = P_{\mathrm{CTD}} \end{cases} \tag{30}$$

The critical Laplace pressures of different surfaces are listed in Supplementary Table 4. The MNSF surface has the highest critical Laplace pressure P_{CCB} of up to 1035Pa, which is almost in accord with the reported ultrahigh Laplace pressure. The MNG, MNGF and MCNP surfaces also have the high Laplace pressure of 954Pa, 912Pa and 897Pa, respectively. The MNS and MNR surfaces are tested as the comparison group of low critical Laplace pressures, which is 465Pa and 534Pa, respectively.

The icing & melting cycle tests were conducted on the six surfaces. Supplementary Figure 28 shows the icing & melting processes on different surfaces. It can be found that except MCNP surface, the high recovery of Cassie-Baxter state cannot be achieved on any of the other five surfaces. Supplementary Figure 29 shows the CA, SA, CDRR, CARR changes of droplets on the six surfaces after an icing & melting cycle. Obviously, only the droplets on the MCNP surfaces realize the dewetting transition during icing & melting cycle while the other five surfaces fail to achieve the dewetting transitions. The melted droplets on the other five surfaces are stuck in the micro-nanostructures to some extent due to the large surface resistances, and the sliding angles after melting are sharply deteriorated. The droplets on the MNG and MNR surfaces even transit to

the high-adhesion Wenzel state after melting. In terms of the experimental results, it is concluded that the dewetting transitions can occur on the surfaces with high Cassie-Baxter stability more easily, but the high Cassie-Baxter stability cannot guarantee the occurrence of dewetting transitions, which are greatly affected by the surface resistances.

Supplementary Discussion 8. Surface resistances for droplet retraction during the melting process

To evaluate the surface resistances for droplet retraction during the melting process, we adopt the sliding movement of droplet in the Wenzel state to analyze the surface resistances. To simplify the model, a virtual detachment of the receding contact line of the droplet with the fixed advancing contact line on the superhydrophobic surfaces is established (Supplementary Figure 30).

During the detaching movement, the subtracting area of solid-liquid interface is transited to the adding area of liquid-vapor interface. Assuming the detachment distance is Δx, the change of the total interfacial energy can be expressed as

$$\Delta G_{\mathrm{s}} = (\gamma_{\mathrm{sv}} + \gamma_{\mathrm{lv}} - \gamma_{\mathrm{sl}}) \cdot \Delta S = \gamma_{\mathrm{lv}}(1 + \cos\theta_0) \cdot \frac{\pi r_{\mathrm{d}} \sin\theta}{\Lambda^2} \cdot \Delta x \cdot A_{\mathrm{total}} \quad (31)$$

While ΔG_{s} denotes the total interfacial energy of this system; ΔS denotes the area change during the droplet sliding; Δx denotes the virtual displacement of the rear contact line; A_{total} denotes the total surface area of the micro-nanostructure in one cell; Λ is the pitch between the centers of two micropillars; θ represents the contact angle of the droplet in the Wenzel state.

The gravity center of the droplet has been shifted down by $\Delta x/2$ along the surface during the detaching movement, therefore the increase of total interfacial energy of the droplet is equal to the decrease of the gravity potential. The relation can be described as

$$\rho g V_{\mathrm{d}} \cdot \frac{\Delta x}{2} \sin\alpha = \gamma_{\mathrm{lv}}(1+\cos\theta_0) \cdot \frac{\pi r_{\mathrm{d}} \sin\theta}{\Lambda^2} \cdot \Delta x \cdot A_{\mathrm{total}} \tag{32}$$

While ρ is the water density, $\rho \approx 960\mathrm{kg/cm^3}$; g is the gravitational constant, $g \approx 9.8\mathrm{N/kg}$; V_{d} is the droplet volume; α is the tilt angle of the substrate.

When the system is in the mechanics equilibrium, the droplet gravity along the surface is balanced by the surface resistance F_{sr}, hence F_{sr} can be expressed as

$$F_{\mathrm{sr}} = 2\gamma_{\mathrm{lv}}(1+\cos\theta_0) \cdot \frac{\pi r_{\mathrm{d}} \sin\theta}{\Lambda^2} \cdot A_{\mathrm{total}} \tag{33}$$

From the above equation, it can be found that the surface resistance F_{sr} is proportional to the total surface area of the micro-nanostructure in one cell. It is well-known that more micro-nanostructures will improve the superhydrophobicity significantly. However, more micro-nanostructures will also lead to the larger surface resistance. To further investigate the relationship between superhydrophobicity and surface resistances as the change of the micro-nanostructure, we take the surface with the double-scale micro-nanostructure (micropillars with nanowires) as an example to analyze the evolution of hydrophobicity and surface resistance with the change of the micro-nanostructure size[9].

The contact angle of the droplet in the W-W state can be calculated by

$$\begin{cases} \cos\theta^{\mathrm{W-W}} = r_2 f_{\mathrm{top}} \cos\theta_0 + f_{\mathrm{top}} - 1 \\ r_2 = 1 + 4f_2 \cdot h_{\mathrm{n}}/d_{\mathrm{n}} \end{cases} \tag{34}$$

While $\theta^{\mathrm{W-W}}$ is the contact angle when droplets are in the Wenzel-Wenzel state, r_2 represents the roughness factor (the ratio of the total surface area to the planar surface area) of the nanostructures; h_n and d_n denote the height and diameter of nanowires, respectively.

Combining (33) and (34), the surface resistance can be further derived as

$$F_{\mathrm{sr}} = 2\gamma_{\mathrm{lv}}(1+\cos\theta_0)\cdot\frac{\pi r_{\mathrm{d}}\sin\arccos[(1+4f_2\varepsilon_{\mathrm{n}})f_1\cos\theta_0+f_1-1]}{\Lambda^2}\cdot[\Lambda^2+2\Lambda H\sqrt{\pi f_1}+2\Lambda f_2\sqrt{\pi f_1}(1+4\varepsilon_{\mathrm{n}})] \quad (35)$$

While ε_{n} denotes the ratio of h_n and d_n, $\varepsilon_{\mathrm{n}} = h_{\mathrm{n}}/d_{\mathrm{n}}$.

Supplementary Figure 31 shows the evolution of the contact angles and F_{sr} with ε_{n}. With the increase of ε_{n}, the micro-nanostructure become more and more abundant, which significantly improve the contact angles (Supplementary Figure 31b), but the surface resistances F_{sr} also increase dramatically (Supplementary Figure 31a). Supplementary Figure 31c depicts the evolution of the maximum surface resistance (when f_1=1, f_2=1) with nanostructures. It can be found that the maximum surface resistance markedly increases with the increase of ε_{n}. When the d_n is fixed at constant, the increase of ε_{n} is equal to the increase of h_n. Therefore, the longer or more nanostructure can lead to the larger surface resistance, which further makes the retraction of the droplet more difficult during the melting process. This conclusion also explains the reason why droplets on some surfaces with high Cassie-Baxter stability cannot realize the dewetting transitions during the icing & melting cycle.

Supplementary Discussion 9. Solar-assisted melting experiments

In the former experiments, the melting process was realized by natural melting. To further investigate whether the W2C transition still occurs under external assisted warming, solar-assisted melting experiments were conducted. The intensity of sunlight is 1kW/m^2 (one-sun light). As shown in Supplementary Figure 32, the solar-assisted melting process is similar to the natural melting processing, where bubbles move downwards continuously to prompt the recovery of the bottom air pockets. After complete melting, the Cassie-Baxter state nearly completely recovers to the original state. Supplementary Figure 33 shows the temperature curves with solar-assisted melting and natural melting. Under the solar assistance, the substrate temperature rises faster.

Supplementary Figure 34 summarizes the effects of three types of external conditions on the recovery of the Cassie-Baxter state. It is obviously found that although the compressing process results in the decrease of recovery extents, the droplets after melting can still greatly recover to the original Cassie-Baxter state.

Supplementary Discussion 10. Effects of different substrate temperature on the dewetting transitions

We conducted the icing & melting cycle experiments of different freezing temperatures (-13℃, -15℃ and -17℃). As shown in Supplementary Figure 35, the effects of different freezing temperatures on the dewetting transitions are not significant. All the melted droplets can recover to the Cassie-Baxter state well.

Supplementary Discussion 11. Effects of different room temperatures on the dewetting transitions

Supplementary Figure 36 shows the icing & melting tests at 20℃ room temperature. The whole icing and melting behaviors of droplets are similar to that at 15℃ room temperature. The droplets after melting also recover to the original CB state.

Supplementary Discussion 12. Effects of different environmental humidities on the dewetting transitions

Supplementary Figure 37 depicts the effects of environmental humidities on the dewetting transitions. The environmental humidity of 20%, 50% and 80% is set to conduct the icing & melting cycle experiments, respectively. It can be found that higher environment humidity results in the slight deterioration of the CB state recovery during the icing & melting cycle, but the overall deterioration extents are not significant. The contact angles and sliding angles of the melted droplets at 80% environment humidity can still reach $155.8° \pm 2.5°$ and $4.6° \pm 1.0°$, guaranteeing the droplets after melting can still be easily removed even at a higher environment humidity. Supplementary Figures 37d and e show the icing and melting processes of droplets at the environment humidity of 50% and 80%, respectively. With the increase of environment humidity, more ice whiskers grow on the ice droplets. During the melting process, the downwards moving bubbles are also captured, which is corresponded to the former experimental results and analyses.

Supplementary Discussion 13. Effect of droplet volumes on the dewetting transitions

Supplementary Figure 38 shows the changes of droplets with different volumes during the icing & melting cycle. It can be observed that the recovery extents of the Cassie-Baxter state gradually decrease with the decrease of droplet volume. When the droplet volume decreases to $1\mu L$, the contact angle after melting decrease to less than $150°$, and the sliding angle increases to $8.48°$.

Meanwhile, we conducted the icing & melting cycle tests for nL-scale droplets. Supplementary Figure 39 depicts the icing and melting processes of the droplet with the volume of $50nL$. The bubbles also move downwards during the melting process, and the air pockets are well recovered. Different with μL-scale droplets, the icing and melting processes of the $50nL$ droplet are quicker due to smaller volume.

Supplementary Discussion 14. Effects of different ice types on the dewetting transitions

The ice in the former experiments is the rime ice, consisting of a lot of bubbles. The glaze ice is obtained by the method of cooling followed by dripping. Supplementary Figure 40 depicts the glaze ice formed in $-10°C$ and $-15°C$. It can be clearly observed that the surface of the glaze ice is smoother and fewer bubbles exist in the glaze ice. During the melting processes, the bubbles moving downwards are captured in Supplementary Figure 40a. Due to the Cassie-frost is covered on the surface before dripping, the formed glaze ice is essentially the Cassie-ice. Therefore, the melted droplets can still keep the Cassie-Baxter state.

Supplementary Discussion 15. Droplet dynamic impacting on the MCNP surfaces

The temperature of water droplets is fixed at $T = 15℃$, which is equal to the ambient temperature. The MCNP surfaces are adhered on the cooling plate with the thermal conductive silicone. When the temperature of the cooling plate decreases to $-15℃$, the droplet is released from a syringe with a specific falling height. In this work, we take four different releasing heights to study the influence of droplet dynamic impacting on the icing & melting cycle. The Weber numbers of the droplets released from four different heights can be obtained by $We = \rho v^2 D_{\mathrm{d}}/\gamma$, where ρ, v, D_{d} and γ denote the mass density of water, impact velocity (i.e. the velocity when the drop touches the substrate), initial diameter of droplets and surface tension of water, respectively. The impact velocity can be calculated by $v^2 = 2gh$, where g is the gravity of acceleration, which is taken as 9.81m/s, and h denote the droplet releasing height. A CCD camera with 10 frames per second is used to record the impacting process. The four types of impacting parameters are displayed in Supplementary Table 5.

Supplementary Figure 41 shows the results of droplets dynamic impacting. When the impacting velocity is 0.5m/s and 1.0m/s, the droplets will bounce when it touches the substrate, and then stabilize on the substrate and freeze immediately. Due to the formation of Cassie-frost on the surface before droplets impacting, the impacting droplets are not essentially pinned in the micro-nanostructure. Therefore, during the melting processes, the semi-melting droplets rotate and slide spontaneously under the effects of moving bubbles in ice droplets. The melted droplets can be easily removed at the low tilt angles. With the increase of droplet impacting velocity, the rebounding process disappears, and the profiles of ice droplets gradually transit from the pear-shaped to the pie-shaped. The tip angles of ice droplets also increase gradually with the increase

of droplets impacting velocity (Supplementary Figure 42). While the pie-shaped profiles of ice droplets result in more contact area between ice and substrate, the spontaneous rotation and sliding are still remained, which even prompt the ice shedding during the melting process.

Supplementary References

1 Hou, Y. et al. Suppressing Ice Nucleation of Supercooled Condensate with Biphilic Topography. Physical Review Letters 120, 075902, doi:10.1103/PhysRevLett.120.075902 (2018).

2 Chu, F. et al. Bubble formation in freezing droplets. Physical Review Fluids 4, 071601(R), doi:10.1103/PhysRevFluids.4.071601 (2019).

3 Liu, W. et al. An integrative bioinspired venation network with ultra-contrasting wettability for large-scale strongly self-driven and efficient water collection. Nanoscale 11, 8940-8949, doi:10.1039/C8NR10003A (2019).

4 Lv, C., Yang, C., Hao, P., He, F. & Zheng, Q. Sliding of Water Droplets on Microstructured Hydrophobic Surfaces. *Langmuir* **26**, 8704-8708, doi:10.1021/la9044495 (2010).

5 Xue, Y., Chu, S., Lv, P. & Duan, H. Importance of Hierarchical Structures in Wetting Stability on Submersed Superhydrophobic Surfaces. *Langmuir : the ACS journal of surfaces and colloids* **28**, 9440-9450, doi:10.1021/la300331e (2012).

6 Boreyko, J. B., Baker, C. H., Poley, C. R. & Chen, C.-H. Wetting and Dewetting Transitions on Hierarchical Superhydrophobic Surfaces. *Langmuir* **27**, 7502-7509, doi:10.1021/la201587u (2011).

7 Pan, R. *et al.* Extremely high Cassie–Baxter state stability of superhydrophobic surfaces via precisely tunable dual-scale and triple-scale micro–nano structures. *Journal of Materials Chemistry A* **7**, 18050-18062, doi:10.1039/C9TA04484A (2019).

8 Pan, R., Zhang, H. & Zhong, M. Ultrafast Laser Hybrid Fabrication and Ice-Resistance Performance of a Triple-Scale Micro/Nano Superhydrophobic Surface. Chinese Journal of Lasers 48 (2021).

9 Pan, R., Zhang, H. & Zhong, M. Ultrafast Laser Hybrid Fabrication and Ice-Resistance Performance of a Triple-Scale Micro/Nano Superhydrophobic Surface. Chinese Journal of Lasers 48 (2021).
