# Supplementary material for: Spontaneous dewetting transitions of droplets during icing & melting cycle
Source: Nat Commun. 2022 Jan 19;13:378. doi: 10.1038/s41467-022-28036-x (PMC8770474; doi:10.1038/s41467-022-28036-x)
Supplement: Supplementary file 2 — Description of Additional Supplementary Files [file 41467_2022_28036_MOESM2_ESM.pdf]

Description of Additional Supplementary Files

File Name: Supplementary Movie 1
Description: Icing & melting cycle of a droplet on the IMN surface

File Name: Supplementary Movie 2
Description: Icing & melting cycle of a droplet on the IMN surface

File Name: Supplementary Movie 3
Description: Icing & melting cycle of a droplet on the IMN surface

File Name: Supplementary Movie 4
Description: Icing & melting cycle of a droplet on the IMN surface

File Name: Supplementary Movie 5
Description: Icing & melting cycle of a droplet on the IMN surface
