# Supplementary material for: Spontaneous dewetting transitions of droplets during icing & melting cycle
Source: Nat Commun. 2022 Jan 19;13:378. doi: 10.1038/s41467-022-28036-x (PMC8770474; doi:10.1038/s41467-022-28036-x)
Supplement: Supplementary file 8 — Lasing Reporting Summary [file 41467_2022_28036_MOESM8_ESM.pdf]

nature research

Corresponding author(s): Minlin Zhong

Lasing Reporting Summary

Nature Research wishes to improve the reproducibility of the work that we publish. This form is intended for publication with all accepted papers reporting claims of lasing and provides structure for consistency and transparency in reporting. Some list items might not apply to an individual manuscript, but all fields must be completed for clarity.

For further information on Nature Research policies, including our data availability policy, see Authors & Referees.

Experimental design

Please check: are the following details reported in the manuscript?

1. Threshold

Plots of device output power versus pump power over a wide range of values indicating a clear threshold

☒ Yes ☐ No

It is shown in Supplementary Figure 45.

2. Linewidth narrowing

Plots of spectral power density for the emission at pump powers below, around, and above the lasing threshold, indicating a clear linewidth narrowing at threshold

☐ Yes ☒ No

Laser company has no relevant data, but the spot energy at full power can be found in Supplementary Figures 46-48.

Resolution of the spectrometer used to make spectral measurements

☐ Yes ☒ No

The data are not available due to the rules of laser company, and this information is not related to our work.

3. Coherent emission

Measurements of the coherence and/or polarization of the emission

☐ Yes ☒ No

The information is not relevant since our work focuses on the icing & melting phenomena on superhydrophobic surfaces, which are not limited to the methods of surface fabrication.

4. Beam spatial profile

Image and/or measurement of the spatial shape and profile of the emission, showing a well-defined beam above threshold

☒ Yes ☐ No

The profile can be found in Supplementary Figures 47 and 48.

5. Operating conditions

Description of the laser and pumping conditions
Continuous-wave, pulsed, temperature of operation

☒ Yes ☐ No

The information is described in Supplementary Method 3.

Threshold values provided as density values (e.g. W cm^{-2} or J cm^{-2}) taking into account the area of the device

☒ Yes ☐ No

The information can be found in Supplementary Table 3.

6. Alternative explanations

Reasoning as to why alternative explanations have been ruled out as responsible for the emission characteristics
e.g. amplified spontaneous, directional scattering; modification of fluorescence spectrum by the cavity

☐ Yes ☒ No

This information is not relevant to our research. Icing and melting phenomena on superhydrophobic surfaces are seldom affected by the emission characteristics.

7. Theoretical analysis

Theoretical analysis that ensures that the experimental values measured are realistic and reasonable
e.g. laser threshold, linewidth, cavity gain-loss, efficiency

☐ Yes ☒ No

This information is not relevant to our research. Our research mainly focuses on the icing and melting phenomena, which is not restricted to other fabricating methods.

8. Statistics

Number of devices fabricated and tested

☐ Yes ☒ No

We don't fabricate or test devices but focus on the icing and melting phenomena of droplets.

Statistical analysis of the device performance and lifetime (time to failure)

☒ Yes ☐ No

The information is shown in Supplementary Table 3.
